# Attractive internuclear force drives the collective behavior of nuclear arrays in *Drosophila* embryos

**DOI:** 10.1371/journal.pcbi.1009605

**Published:** 2021-11-19

**Authors:** Xiaoxuan Wu, Kakit Kong, Wenlei Xiao, Feng Liu

**Affiliations:** 1 Center for Quantitative Biology, Peking University, Beijing, China; 2 State Key Laboratory of Nuclear Physics and Technology, School of Physics, Peking University, Beijing, China; 3 School of Mechanical Engineering and Automation, Beihang University, Beijing, China; 4 Key Laboratory of Hebei Province for Molecular Biophysics, Institute of Biophysics, School of Health Science & Biomedical Engineering, Hebei University of Technology, Tianjin, China; National Institutes of Health, UNITED STATES

## Abstract

The collective behavior of the nuclear array in *Drosophila* embryos during nuclear cycle (NC) 11 to NC14 is crucial in controlling cell size, establishing developmental patterns, and coordinating morphogenesis. After live imaging on *Drosophila* embryos with light sheet microscopy, we extract the nuclear trajectory, speed, and internuclear distance with an automatic nuclear tracing method. We find that the nuclear speed shows a period of standing waves along the anterior-posterior (AP) axis after each metaphase as the nuclei collectively migrate towards the embryo poles and partially move back. And the maximum nuclear speed dampens by 28-45% in the second half of the standing wave. Moreover, the nuclear density is 22–42% lower in the pole region than the middle of the embryo during the interphase of NC12-14. To find mechanical rules controlling the collective motion and packing patterns of the nuclear array, we use a deep neural network (DNN) to learn the underlying force field from data. We apply the learned spatiotemporal attractive force field in the simulations with a particle-based model. And the simulations recapitulate nearly all the observed characteristic collective behaviors of nuclear arrays in *Drosophila* embryos.

## Author summary

The emerging collective behaviors during embryogenesis play an important role in precise and reproducible morphogenesis. An important question in the study of collective behaviors is what rule underlies the emerging pattern. Here we use the *Drosophila* embryo as a test tube and focus on the nuclear array without membrane separation on the embryo periphery from NC11 to NC14. We quantify the collective behaviors of the nuclear array from NC11 to NC14 in intact embryos with light-sheet microscopy and automatic image processing. We discover that during NC11-14 the collective motion of the nuclear array after the mitotic wave shows a damped standing wave, and the stabilized nuclear density after the collective motion is higher in the middle of the embryo. With a DNN, we successfully extract a spatiotemporal attractive internuclear force field from the experimental data. This force field outperforms previously proposed force fields, recapitulating all the known characteristic features of the nuclear array in 3D simulations. The DNN-based method demonstrated in our study could be a tool in extracting the force field underlies the other complex collective behavior.

## Introduction

The emerging collective behaviors during embryogenesis are key to understand the origin of the precise and reproducible morphogenesis [1–3]. In general, complex collective behaviors (such as cytoskeleton filament arrangement, morphogenesis, and bird flocks) arise from interactions between many similar units (such as cytoskeleton macromolecules, cells, and birds) [4]. To understand the mechanism underlying the collective patterns, it is essential to uncover the interaction rules between individual units [5–9]. However, it has been challenging to reversely infer the interaction rule from the collective motion data.

The nuclear array in the early embryo of *Drosophila melanogaster* is an excellent model system to address this question. During the first 13 nuclear cycles (NCs), the whole embryo is a synplasm, in which nuclei share a common cytoplasm and the corresponding molecular environment [10,11]. From NC10 to NC14, the nuclei distribute near the embryo periphery to form a two-dimensional (2D) nuclear array [10,11]. After each nuclear cycle, the density of the nuclear array doubles, and the internuclear distance decreases. The spatial and orientation orders increase from early to later nuclear cycles [12], but the radial distribution functions overlap if they are rescaled with the nuclear density [13]. At the end of each nuclear cycle, a pseudo-synchronous mitotic wave usually starts from the two embryo poles, moves towards the middle of the embryo along the anterior-posterior (AP) axis. A collective “yo-yo”-like nuclear motion follows the onset of anaphase, i.e., the nuclei move towards the two poles then back nearly to the original position [3].

However, the mechanism controlling these patterns remains to be elucidated. An essential reason is the underlying complicated molecular dynamics [14–17]. During the interphase, each nucleus is surrounded by a microtubule basket and has an Arp2/3 nucleated actin cap attached to the membrane. In the internuclear region, myosin II and linear actin filaments form an actomyosin complex adhering to the membrane. During the mitotic (M) phase, the actin cap region enlarges, and the internuclear membrane invaginates to form mitotic furrows surrounding the spindles. These two states alternate over the repeated nuclear cycles.

These nucleus-centered and surrounding cytoskeletal elements generate the internuclear interaction force, which could be either attractive or repulsive. On the one hand, the active force generated by overlapping microtubule baskets and the attached motors (e.g., kinesin-5) could be repulsive [12,18,19]. The repulsive force could also originate from the passive response of the elastic nuclear-embedding cortex [20]. On the other hand, actomyosin borders could generate attractive forces [1,2,17,21–23]. Microtubules attached with the motor dynein may also provide attractive forces [18]. The actin cap restricts the nuclear movement [24] and microtubules interact with actin caps via dynein-dynactin complexes [24–26]. In the absence of actin caps, adjacent nuclei collide [24].

Several particle-based models have been proposed with different formulas of the pair-wise force between nuclei [3,12,13]. And nearly all of these models assume that the predominant internuclear force is repulsive. Through parameter fitting, each model appears to recapitulate some specific feature of interest. However, a coherent force field has yet to be tested to account for all the observed characteristic features of the nuclear array.

Inspiring by the idea “from data to rules”, we use a deep neural network (DNN) to directly learn internuclear interaction rules from the experimental data. This new approach has several advantages. First, without any arbitrary assumption of the specific formula, the learned rules could be unbiased to show the integrated effect of all of the related molecules. Second, since the DNN is a universal approximation to any Borel measurable function with the desired degree of accuracy [27,28], the potential complex rules can be accurately represented. Last, benefitting from the development of TensorFlow [29], the fitting process is really efficient. With the input of the nuclear age and nuclear density, the network outputs the internuclear force. The training error is calculated according to the resultant force and nuclear speed.

Through learning, we find an attractive force field, which is strongly temporal-dependent and positively correlated with the internuclear distance. To account for the nucleus size in the simulations with the particle-based model, we add a repulsive force in the short internuclear distance. We confirm that this force field outperforms the previously proposed repulsive force field, recapitulating nearly all the observed characteristic collective behaviors of the nuclear array in *Drosophila* embryos. In particular, following the pseudo-synchronous mitotic wave initiating from the low-density embryo poles, this attractive internuclear force drives the collective nuclear motion with a damped standing wave of the nuclear speed, and gradually reduces the non-uniformity of the nuclear density from NC11 to NC14.

## Results

### Nuclear array shows a stereotypic packing pattern and a collective motion pattern from NC11 to NC14

**Fig 1:**
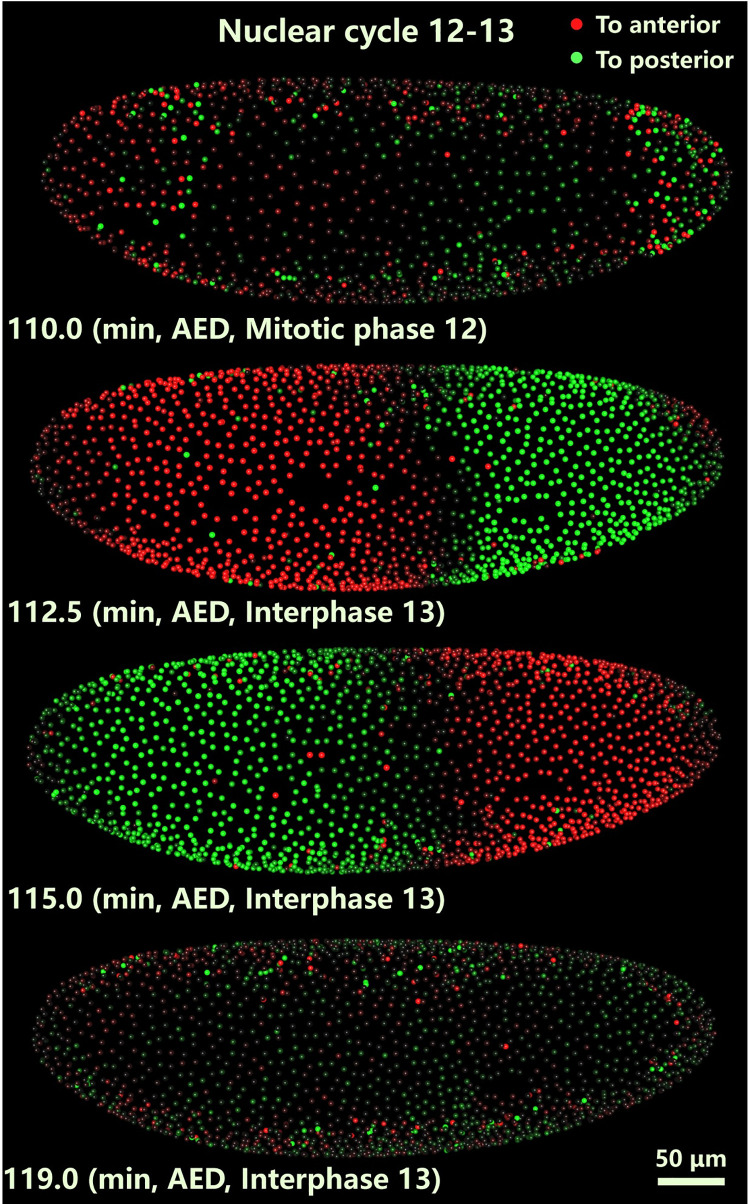
Representative images of the collective motion of the nuclear array of a *Drosophila* embryo (snapshots from S1 Movie). These images are the projections from the 3D images taken with light sheet microscopy. Each nucleus is segmented and marked with a different color to show the direction of the nuclear velocity along the AP axis (red and green represent the left (anterior) and right (posterior) direction, respectively. And the intensity indicates the magnitude of the nuclear velocity).

**Fig 2:**
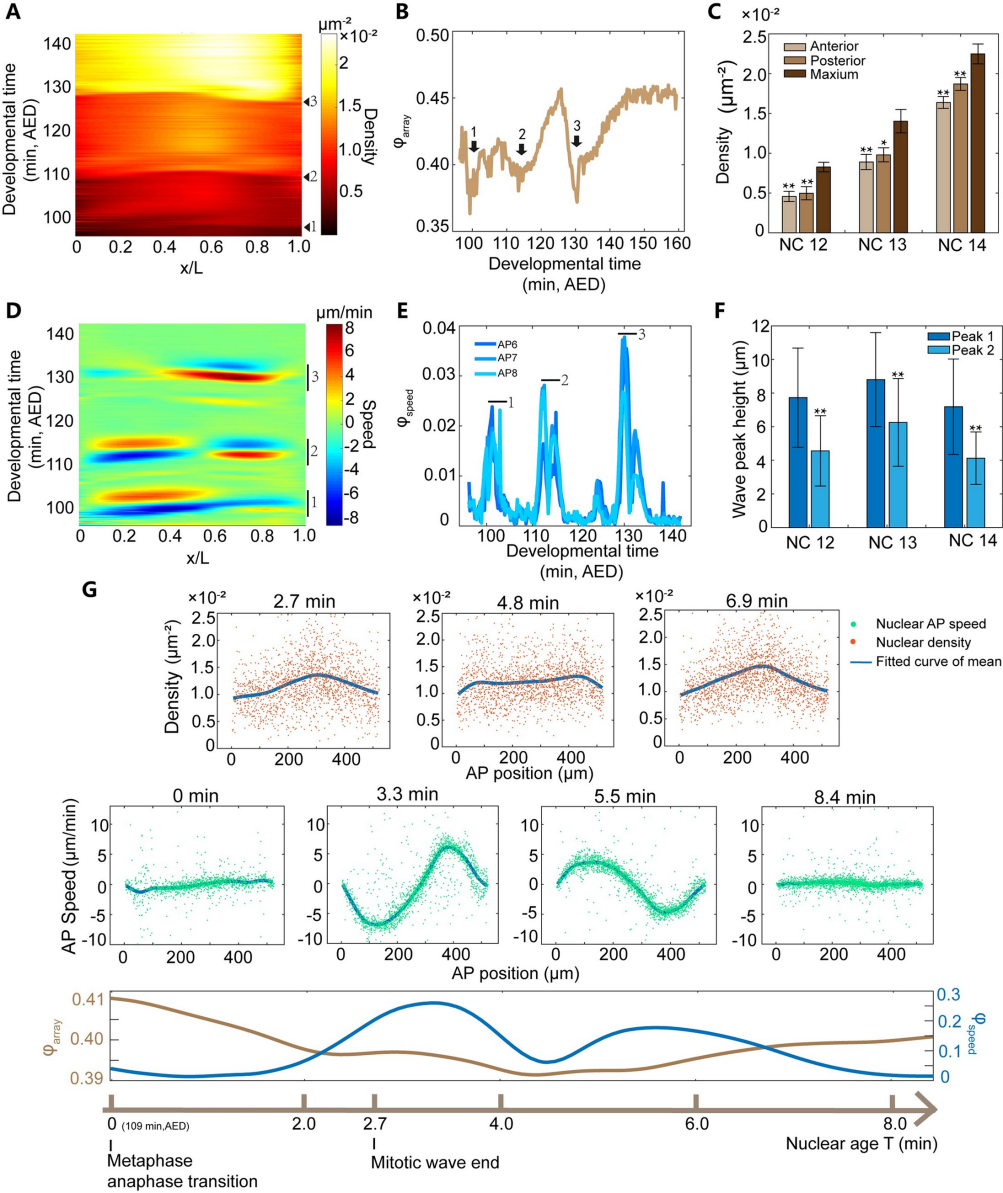
Characteristic features of the packing pattern and the collective motion pattern of the nuclear array in a representative *Drosophila* embryo from NC11 to NC14. (A) A representative heat map of the projected nuclear density along the AP axis of the embryo from NC11 to NC14, i.e., rescaled developmental time from 90 min to 150 min after embryo deposition (AED) at 25°C (see S1 Text). Black triangles label the start time point of chromosome segregation during mitotic phase (M phase) 11, 12 and 13. (B) The dynamics of hexatic bond-orientational order parameter *φ*_*array*_ (see Materials and methods). The black arrows 1, 2 and 3 label three time intervals around M phase 11, 12 and 13 showing the minimum order parameter. (C) Bar graph (mean ± standard deviation (SD)) comparing anterior (~5–15% EL) or posterior (~85–95% EL) density and the maximal density in the middle of the embryo during interphase. Student’s *t*-test results: interphase 12 (anterior, *p*<0.01; posterior, *p*<0.01; embryo number *n* = 4); interphase 13 (anterior, *p*<0.01; posterior, *p* = 0.015; *n* = 4); interphase 14 (anterior, *p*<0.01; posterior, *p*<0.01; *n* = 4). (D) A representative heat map of the nuclear speed projected along the AP axis of the embryo from NC11 to NC14. Positive and negative direction point to the posterior and anterior pole, respectively. (E) Dynamics of the order parameter of the collective motion (*φ*_*speed*_, see Materials and methods) from NC11 to NC14. APi (*i* = 6–8) corresponds to the *i*^*th*^ bin with the width of 10% EL from the anterior pole (for the other bins, see S6 Fig). The markers 1, 2 and 3 label three time windows that show high motion collectivity in (D) and (E). (F) Bar graph comparing the maximal wave crest of the first half period and the second half period of the AP speed standing wave. Student’s t test results: NC12 (p<0.01; *n* = 4); NC13 (p<0.01; *n* = 4); NC14 (p<0.01; *n* = 4). (G) The corresponding dynamics of the nuclear density, speed, smoothed *φ*_*array*_ and smoothed *φ*_*speed*_ of the nuclear array after the onset of anaphase 12 shown in (A-D).

To quantify the packing and collective motion patterns of the nuclear array in *Drosophila* embryos, we image H2Av-GFP expressing embryos from NC10 to NC14 with light sheet microscopy, which has low phototoxicity and high temporal resolution (Materials and methods). With an automatic segmentation and tracking algorithm based on Tracking with Gaussian Mixture Models (TGMM) [30,31], we obtain the nuclear position and speed (Fig 1 and S1 Movie). We discover that the nuclear array shows a stereotypic packing pattern and collective motion pattern during the early developmental process, and these patterns are conservative across embryos (Figs 2 and S1–S7).

Intuitively, one might think that these nuclei are uniform-sized and arranged as hexagonal close packing, which is the closest packing on a plane. However, the nuclear density shows a non-uniform distribution along the AP axis (Fig 2A). During the interphase from NC12 to NC14, both the nuclear density of the anterior (~5–15% embryo length (EL)) and posterior (~85–95% EL, to avoid the potential influence of pole cells on calculating the nuclear density) poles are significantly less than the maximal density in the middle of the embryo (Figs 2C, S1 and S2), i.e., the nuclear density is relatively 22–42% lower in the pole region than that in the middle of the embryo (Fig 2C). Note that as the nuclear number doubles, the nuclear distribution becomes more uniform. Previous results measured with fixed embryos also show a higher nuclear density in the middle along the AP axis in NC14 [32,33]. And the anterior nuclear density is less than the one in the middle region for NC11-14 in both live and fixed embryo measurements [34]. Moreover, in the interphase from NC11 to early NC14 before cellularization, the hexatic bond-orientational order parameter [24,35–37] increases from 0.35 to 0.45 (Figs 2B and S3), significantly less than 1 (the order parameter of a perfect hexagonal array). This result is consistent with the order parameter calculation in NC13-14 in a previous study showing that the proportion of the nuclei with six neighbors is ~56% [24].

The density distribution and the regularity of the nuclear array are not always stable, they dynamically change as a function of the nuclear age *T* (defined as the time elapse after the onset of anaphase, i.e., the start time point of chromosome segregation). For instance, the higher nuclear density distribution in the middle of the embryo established after the mitotic wave at *T* = ~2.7 min unifies along the AP axis temporally when *T* = ~4.8 min and is restored at *T* = ~6.9 min (Fig 2G). Meanwhile, during interphase 13, the packing order of the nuclear array drops dramatically, then is restored in a biphasic manner (a fast phase followed by a low one) (Figs 2B and S3).

As the nuclear packing pattern dynamically changes, a collective nuclear motion pattern emerges following the mitotic wave (Fig 2D, 2E, and 2G). Just after *T* = 0 min, the nuclei move towards the embryo poles, then after *T* = ~8.8 min (S4 Fig), they partially move back to the original position based on the nuclear trajectory plot (S5 Fig). This phenomenon is consistent with the recently reported “yo-yo”-like movement [3]. The motion collectivity (calculated with the order parameter *φ*_*speed*_, see Materials and methods) peaks during this collective motion process (Figs 2E and S6). And the nuclear speed of each collective motion projected to the AP axis shows a standing wave (Fig 2G middle and S2 Movie). This standing wave only lasts for one period of approximately 8.8 min (S4 Fig). It has 3 nodes locating at the two embryo poles and the embryo middle. The maximum nuclear displacement decreases from the positions of the crest to the node of the standing wave (S5 Fig). Besides, the amplitude of this standing wave damps. For instance, during interphase 13 the maximum average nuclear speed reaches 8 μm/min at *T* = ~3.3 min in the first half period, then decreases by ~30% to 5 μm/min at *T* = ~5.5 min in the second half (Fig 2G middle). If we calculate the wave peak ratio, which is defined as the ratio between the maximal wave crest of the second half period and the first half period of the AP speed standing wave, it is ~0.63 on average from NC12-14 (Fig 2F). As a result, the nuclei do not fully recover to the original AP position (S5 Fig).

Although the chromosome segregation process is isotropical in orientation [3], the collective nuclear motion along the AP axis is significantly more pronounced than the one along the dorsal-ventral (DV) axis. Compared with the AP speed, the DV speed is very low (S7 Fig). Just after *T* = 0 min, the nuclei slowly move to the dorsal side and then move back (S7 Fig). The maximum average DV speed is just 0.5 μm/min, much smaller than the maximum AP speed of 8 μm/min (Figs 2D and S7). And the DV speed is noisy. The absolute DV speed of each nucleus varies from 0 μm/min to 1.3 μm/min (S7A Fig and S3 Movie). The standard deviation of the DV speed doubles during the collective motion process.

### An attractive force field learned by DNN shows a pulse-shape dependence on nuclear age and a linear dependence on internuclear distance

To understand the origin of the observed nuclear packing and the collective motion pattern, it is important to know the interaction rule between nuclei. The nuclei share a common cytoplasm from NC11 to early NC14 [10,11] and the surrounding cytoskeleton dynamically changes during each nuclear cycle [14–17], indicating that the spatiotemporal interaction rule between each pair of the nearest neighboring nuclei is identical. Here the spatiotemporal interaction rule is the internuclear force varying with space and time.

The internuclear force can be derived from the motion equation. As an empirical assumption, the overdamped equation has been extensively used for nuclear packing and motion in *Drosophila* embryos [3,12,13], nuclear positioning in muscle cells [18], and cell positioning in *C*. *elegans* embryos [38–40]. Hence, we adopt the same approach for the sake of comparison with previous work [3,12,13], although the overdamped equation could be oversimplified to describe the viscoelastic property of *Drosophila* embryos [20]. Moreover, considering the collective motion and packing pattern of the nuclear array are predominant along the AP axis (Figs 2 and S7), to eliminate the potential noise of single nuclear data and captures the main features of the dataset, 3D single nuclear data is converted to a 1D dataset consisting of a list of nuclear array units (for more details, see Materials and methods). For convenience of the subsequent deep learning method to learn the internuclear force from this 1D dataset, we implement a 1D overdamped equation to describe the nuclear motion: $\sum_{j\in n(i)}{\bar{\rho }}_{i,j}{\vec{F}}_{i,j}=\tilde{\gamma }{\vec{V}}_{i}$ (Eq (2) in the Materials and methods), here ${\bar{\rho }}_{i,j}$ and ${\vec{F}}_{i,j}$ are the average nuclear density and the average pairwise internuclear force of the neighboring *i*^*th*^ and *j*^*th*^ nuclear array unit, ${\vec{V}}_{i}$ is the average velocity of the *i*^*th*^ nuclear array unit, and $\tilde{\gamma }$ is the effective friction coefficient. We assume that the magnitude of ${\vec{F}}_{i,j}$ is the function of nuclear density and the nuclear age *T*.

Notably, we focus on only the overall function form of ${\vec{F}}_{i,j}$, but not the specific form of its active or passive force components contributed from individual cytoskeletal elements such as myosin, F-actin, or microtubules. Hence ${\vec{F}}_{i,j}$ could be either attractive or repulsive (S8A Fig right panel), we call the attractive force field as F^a^ and the repulsive force field as F^r^. The magnitude of internuclear force can be learned via a classical multilayer feedforward network (MLFNN) (for the details of the deep learning methods, see Materials and methods).

**Fig 3:**
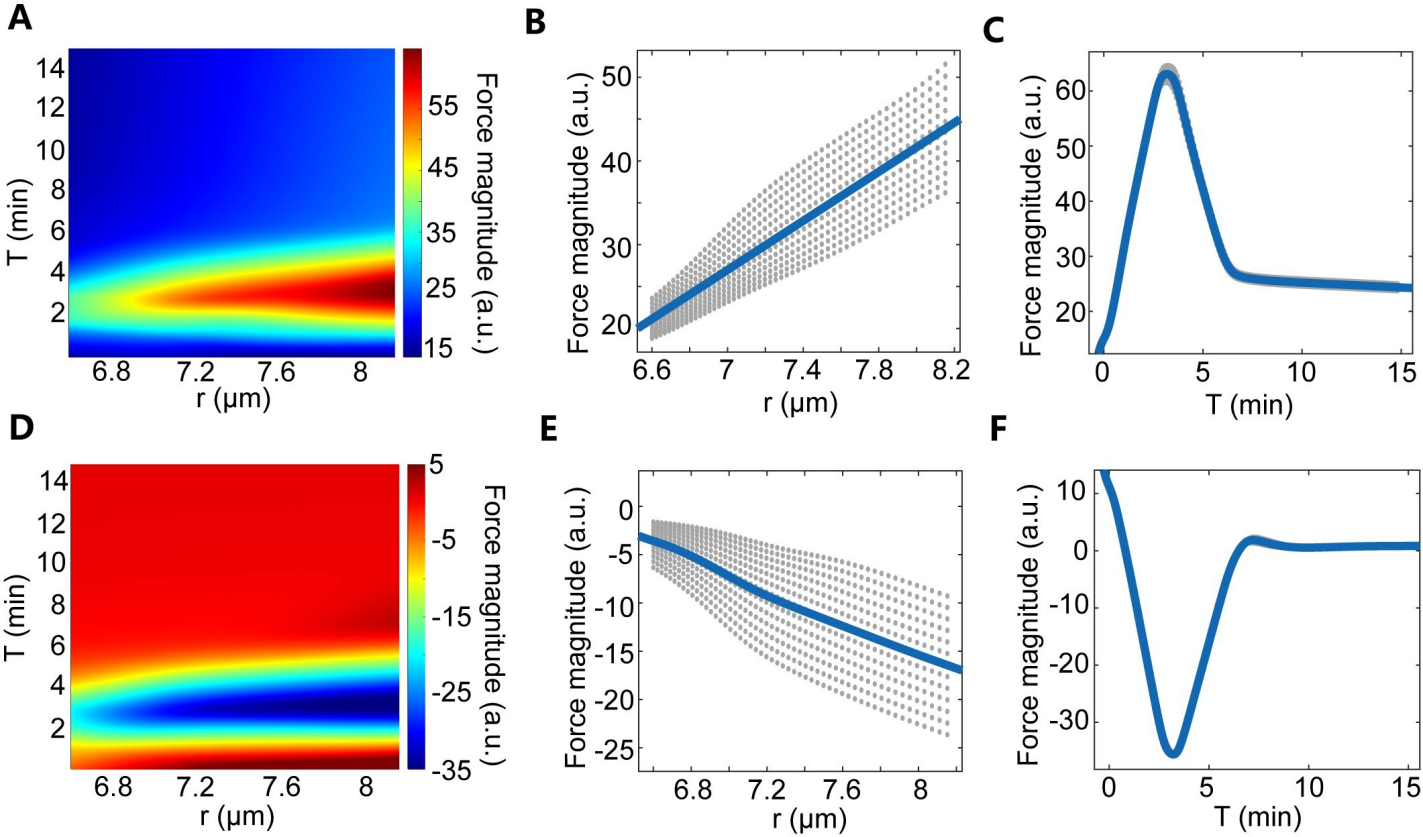
Force field functions learned from 1D data via the MLFNN model. (A-C) The DNN learning results based on the F^a^ assumption. The data from M phase 13 to interphase 14 in one embryo (3078 data points in total) is used while training the DNN. (A) A representative heat map of the function *F*(*T*,*r*). Here *F* is the magnitude of the internuclear force ($F_{i,j}^{learned}$), *T* is the nuclear age after the onset of anaphase, and *r* is the internuclear distance. Note that, $r=\sqrt{s}$ and $\rho =\frac{1}{s}$, here *s* is the Voronoi area of each nucleus. (B) *F* has a positive correlation with *r* as *T* = 4.4–5.6 min. (C) *F* has a pulsatile relationship with *T* as *r* = 7.9–8.2 μm. (D-F) The DNN learning results based on the F^r^ assumption as in A-C.

The trained MLFNN models show that valid solutions could only be found based on the F^a^ assumption (Fig 3A). Under the F^a^ assumption, the magnitude of ${\vec{F}}_{i,j}$ (*F*_*i*,*j*_) is positive as expected. It shows pulsatile dependence on the age of the nuclear array unit (${\bar{\tau }}_{i,j}$), increasing along with ${\bar{\tau }}_{i,j}$ from *T* = 0 to a maximum value then decreases to a constant low level (Figs 3A, 3C and S10). *F*_*i*,*j*_ also increases as the nuclear density decreases, i.e., it increases as the internuclear distance *r* increases (Figs 3A and 3B and S10). And the force field forms from the MLFNN models in many different training trials are conservative (S11 Fig). However, the resultant *F*_*i*,*j*_ based on the F^r^ assumption is negative. Actually, it is approximately the opposite number of the corresponding *F*_*i*,*j*_ based on the F^a^ assumption (Fig 3D–3F). And its effect on nuclear motion is equivalent to the attractive force field. Even if we force the DNN to find a positive solution by changing the activation function, it either could not converge or converge with solutions requiring a repulsive force increasing with distance, which is an unstable force field (S12 Fig). For more details to rule out the F^r^ force field see S4 Text. To avoid any potential bias of the DNN for the attractive force field, we also run a control DNN training based on the data input from the 3D particle-based simulation using a repulsive force filed which decreases with distance (as described in the next section). We confirm that the DNN can successfully recover the characteristic features of the ground-truth force field function just as the attractive force field (for more details see S5 Text and S13 Fig).

Notably, if we ignore the nuclear density difference along the AP axis in the training, i.e., removing ${\bar{\rho }}_{i,j}$ from Eq (4) in the Materials and methods part, $F_{i,j}^{learned}$ no longer represents the magnitude of the average pairwise internuclear force between neighboring nuclear array units but the magnitude of the resultant force acting on the nuclear array from one direction. It remains nearly a constant as the internuclear distance varies, but still shows very similar age-dependence (S14 Fig). Moreover, we confirm that the age–dependence of the internuclear force form learned from MLFNN models is consistent with a deterministic 1D mean-field physical model without density correction. Based on the overdamped momentum equation, we mathematically derive the force from the data (S6 Text). We find that the internuclear force predominantly depends on the nuclear age and obeys a similar pulsatile curve as the learning results from DNN training (S15C and S15D Fig).

### A particle-based model using the F^a^ force assumption regenerates the packing pattern and the collective motion pattern of the nuclear array

To test whether the force field learned from MLFNN models captures the observed characteristic collective behavior of the nuclear array, we run discrete particle dynamics simulations using the force field derived from the MLFNN models (see Materials and methods). To mimic the physiological features of the embryos, we run 3D simulations on a prolate spheroid surface (see Materials and methods and S7 Text) and take the mitosis process into consideration.

Because the training data for the MLFNN model ranges in the internuclear region, the learned attractive force field does not contain the intranuclear region. To account for the nuclear size in the particle-based model, we add a repulsive force in the region with short internuclear distance to complement the learned attractive force field, and call it F^a^ force field afterwards (S16A Fig). Previous studies show that the distance-dependent force field has a “core region” and a “border region” [13]. Usually, the core region consists of the actin caps enforcing the repulsive force. And the actomyosin border (border region) acts like a spring to provide attractive force [14,15], hence the attractive force increases along with distance as we learned from the MLFNN models (S16A Fig). We confirm that if we get rid of the repulsive core region, the F^a^ force field cannot maintain a regular nuclear array during interphase in the simulation (S17 Fig). Although no valid solution is learned from MLFNN based on the F^r^ assumption, we still consider an F^r^ force field with only the repulsive force in the simulation for comparison. Based on the previous models, we assume the repulsive force decreases along with distance in both the core region and the border region (S16B Fig) [3,12,13].

For simplification, we assume that the force fields are the multiplication of the age-dependent force *A(T)* and the distance dependent force *B(r*) (S16A and S16B Fig). For F^a^ force field, *A(T)* is represented as a trigonometric function to mimic the age-dependent force field in Fig 3C and *B(r*) is represented as a linear function to mimic the distance-dependent force field in Fig 3B (see Materials and methods). The simplified F^a^ force field has a similar form with the F^a^ force field derived from the MLFNN models except for the “core region” (Figs 3A and S16A).

**Fig 4:**
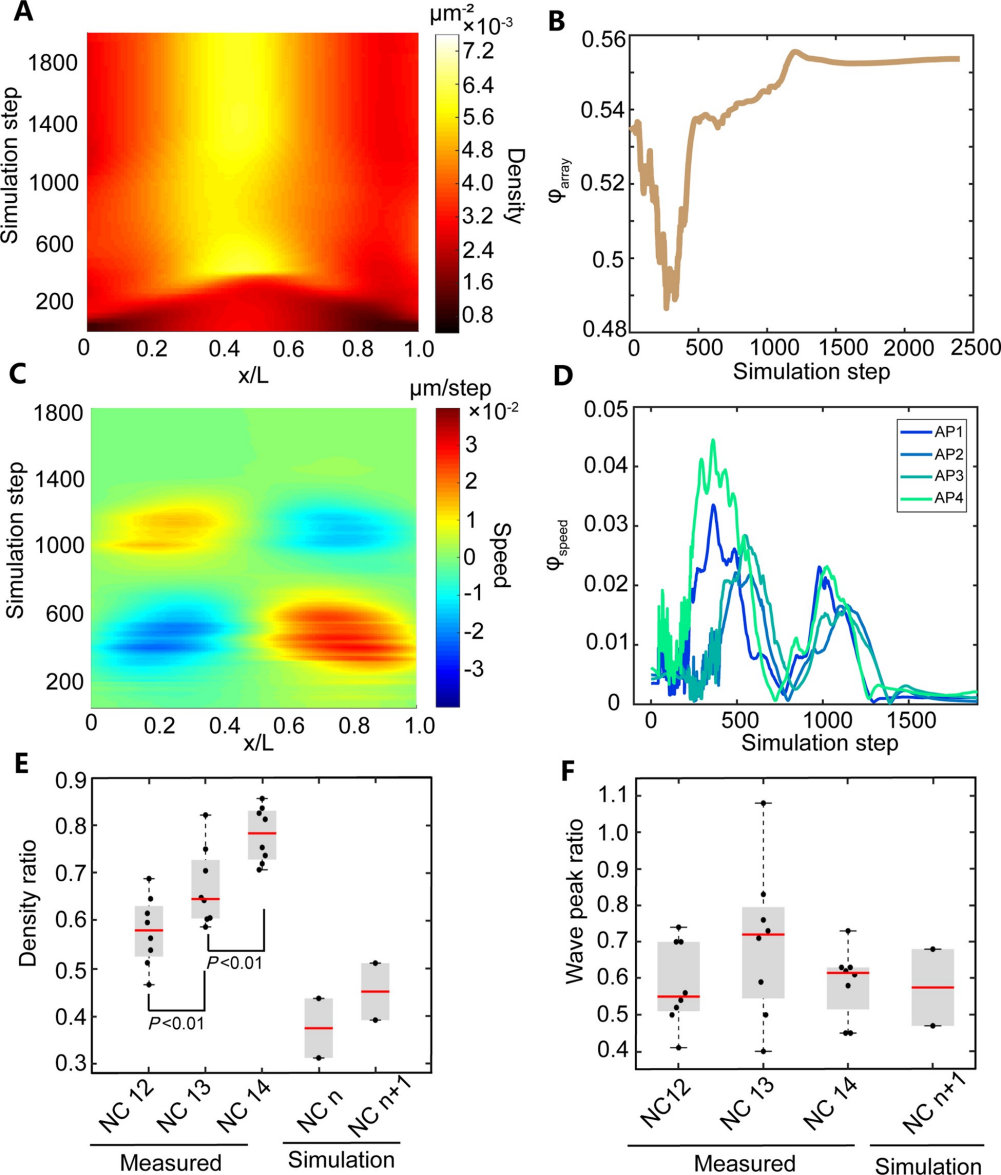
3D simulation results of the packing pattern and the collective motion pattern of the wild type embryo based on the F^a^ force assumption (for the simulation movie, see S4 Movie). (A) Heat map of the nuclear density projected along the AP axis. (B) The dynamics of hexatic bond-orientational order parameter ***φ***_***array***_ (see Materials and methods). (C) Heat map of the nuclear speed projected along the AP axis. (D) Dynamics of the order parameter of the collective motion ***φ***_***speed***_ (see Materials and methods). APi (*i* = 1–4) corresponds to the *i*^*th*^ bin with the width of 25% EL from the anterior pole (E) Boxplots (whisker, min/max values, boxes, 25/75 percentiles). The medians (red line) of measured and simulated density ratio are 0.58, 0.65, 0.78, 0.37 and 0.45, respectively. Density ratio is defined as the ratio between the anterior (~5–15% EL) or posterior (~85–95% EL) density and the maximal density in the middle of the embryo during interphase. (F) Boxplots (whisker, min/max values, boxes, 25/75 percentiles). The medians (red line) of measured and simulated wave peak ratio are 0.55, 0.72, 0.62 and 0.58, respectively. Wave peak ratio is defined as the ratio between the maximal wave crest of the second half period and the first half period of the AP speed standing wave. The force field used in this simulation is shown in S16A Fig.

The simulation results confirm that only the simulation based on the F^a^ force field recapitulates the characteristic features of the nuclear array. The nuclear density stabilizes at a distribution that is higher in the middle and lower in the poles during interphase (Fig 4A). To quantify the non-uniformity, we define the density ratio as the ratio between the anterior (~5–15% EL) or posterior (~85–95% EL) density and the maximal density in the middle of the embryo during interphase when the nuclear array is stable. The density ratio of the simulation data increases after mitosis as observed in the experiment (Fig 4E). However, the simulated density ratio is slightly less than the measured value, and this discrepancy can be removed if we double the nuclear density in the simulation (S18 Fig). Moreover, just after *T* = 0 min, the nuclei migrate towards the embryo poles and then partially move back collectively (Fig 4C and S4 Movie). The simulated AP trajectory recapitulates the experimental features (S19 Fig). The maximum nuclear displacement decreases from the positions of the crest to the node of the standing wave and the nuclei are not fully recovery to the original positions (S19A and S19B Fig). Consistent with the previous experimental result [3], the simulation shows that faster mitotic waves associate with smaller maximum displacement (S19C Fig). And the wave peak ratio in the simulation agrees with our experimental data (Fig 4F). The simulated order parameters (Fig 4B and 4D) of the nuclear array are also comparable with the experimental value during interphase (Fig 2B and 2D). In contrast, based on the F^r^ force field, the nuclear array stabilizes at a nearly uniform density distribution during interphase (S20A Fig), the order parameters of the nuclear array are much higher than the experimental value during interphase (S20B Fig), and two extra collective motion processes show up before and after the experimentally observed motion along the AP axis (S20C and S20D Fig and S6 Movie). Hence the attractive force field outperforms the repulsive force field in the 3D simulations. We further confirm that this result holds for the F^a^ force field with different formula of the attractive force as long as it is positive-pulse shaped with time and positively correlated with distance, e.g., the function *B(r)* with a higher order dependence on *r* as frequently been utilized in previous work (see Materials and methods, S21 Fig and S5 Movie) [3,12].

### The prediction based on the F^a^ force field function is confirmed with the observed nuclear motion patterns

To further test the F^a^ force field learned from MLFNN, we apply it in three extra conditions, in which the experimental observation has not been explicitly utilized in the target function in the model training.

**Fig 5:**
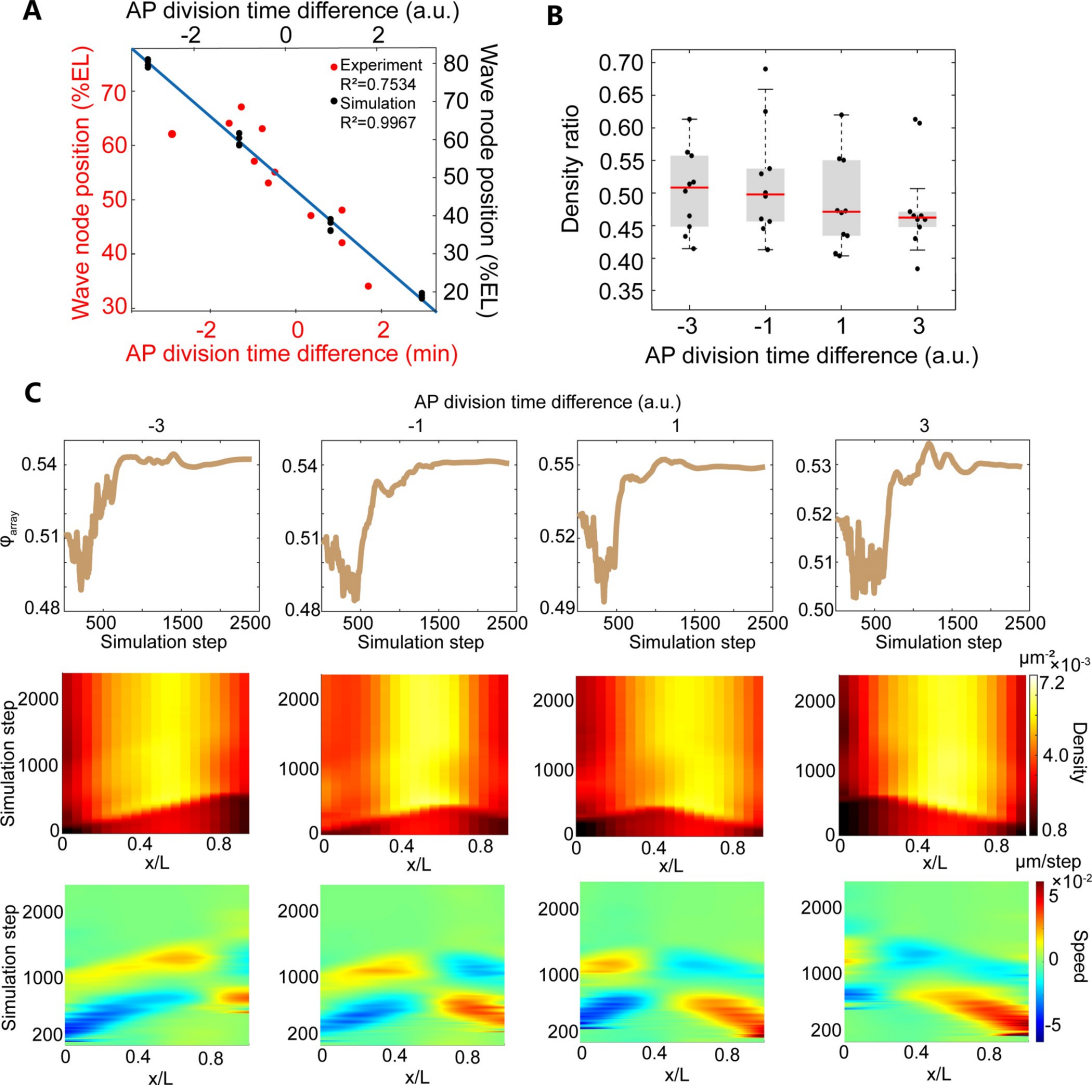
3D simulation results of the embryos with varied start time of metaphase at two poles (F^a^ force assumption). (A) The linear relation between the position of the second node of the standing wave of AP nuclear speed and the division time difference between the anterior and posterior poles of experimental (red) and simulation (black) data. (B) The density ratio of the simulation data with different AP division time difference. Boxplots (whisker, min/max values, boxes, 25/75 percentiles). The medians of the four group data in the panel are 0.51, 0.50, 0.47 and 0.46, respectively. (C) The representative characteristic features of the collective motion pattern and packing pattern of the nuclear array in the simulations with different AP division time difference. The force field used in this simulation is shown in S16A Fig.

In the training dataset shown as the collective motion after the M phase 12 in Fig 2D, the nuclear position with the maximum speed, i.e., the anti-node position of the standing wave of the AP speed, is at 25% EL from the embryo pole. This appears to be a coincidence as it only shows up when the two mitosis waves from the two poles nearly synchronize. As we vary the start time of the two mitosis waves, the anti-node position in the middle also shifts linearly away from the pole where the leading wave initiates (Fig 5A and 5C). Indeed, in the experiment, we also observe a linear change of the middle anti-node position from 30% EL to 70% EL as the start time of the mitosis wave from the two poles differs from -3 min to 3 min (Fig 5A). And the density ratio has no significant change compared to the synchronized division case (Figs 4E and 5B).

In an extreme case, the mitotic wave could reach the other pole before the initiation of the opposite mitotic wave. We simulate this condition and find that the standing wave of the AP speed changes to be two-node (S22A Fig). But the final density distribution after mitosis and the dynamics of the pack order are still similar to the one with two mitotic waves (S22A Fig). This condition actually exists in a small number of wild type embryos. And our simulation results are consistent with the experimental results observed in these embryos [41].

The standing wave of the AP speed could further be adjusted to be 5-node if fastening the embryo in the middle. Our simulation is also consistent with the experiment [42]. Again this does not change the density distribution during interphase and the level of nuclear array regularity compared with wild type embryos (S22B Fig).

## Discussion

Light sheet microcopy helps to reveal the collective packing and motion pattern of the nuclear array in *Drosophila* embryos. As an excellent model system of collective motion, the nuclear array in *Drosophila* embryos has already been reported in several studies [2,3]. Nearly all the previous nuclear array imaging data were obtained with confocal microscopy [2,3,12,13,24]. Due to the limited imaging speed, the embryos were often softly pressed to generate a flat surface filled with nuclei in a shallow depth. It has been reported that the press on *C*. *elegans* embryos could induce profound change in cell movement during embryogenesis [43,44]. The movement of the nuclear array in *Drosophila* embryos could also be affected by the mechanical stress in pressing embryos. Here we use a light sheet microscope for 3D imaging. Instead of imaging only several micrometers in depth below the flattened embryo surface with confocal microscopy, we could image nearly the whole embryo with the comparable overall time resolution. Moreover, phototoxicity is much lower for longer live imaging time. Most importantly, benefitting from the sample preparation method (embedding the embryo in agarose), the embryo has no deformation and the natural membrane curvature is maintained. This leads us to find a stereotypical density distribution along the AP axis: the nuclear density is relatively 22–42% lower in the pole region than that in the middle region of the embryos during the interphase of NC11-14 (Fig 4E). We also discover that the collective motion of the nuclear array shows a 3-node standing wave in the AP speed for the first time. This standing wave only lasts for one period, and the AP speed damps by 28–45% in the second half of the period. Besides these new discoveries, we also confirm several collective behaviors reported in previous publications. For example, the order parameter is significantly less than the perfect value and slowly increases in late NC. It decreases upon the onset of anaphase and recovers lightly above the original value in a biphasic manner [13,24]. And the collective motion shows a high transient collectivity order. These features of the collective behaviors of the nuclear array provide stringent constraints to validate the force field functions used in the simulations.

Reversal engineering with DNNs successfully extracts the net force field governing the collective behavior of the nuclear array in *Drosophila* embryos. Although several particle-based models for the nuclear array have been reported, none of them can account for the nuclear collective motion pattern discovered in our study. For example, the model in ref. [13] cannot generate any collective motion at all. The model in ref. [45] generates a collective motion with the opposite direction. The model in ref. [3] shows similar results as our repulsive force simulation results, i.e., extra motion appearing before and after the normal motion process. Actually our model recapitulates nearly all the reported nuclear packing and motion pattern with only 5 free parameters, much less than the 11 free parameters in ref. [3]. Hence the better agreement between the model simulation and the experimental results is unlikely from overfitting using extra parameters. Instead of giving an ad hoc formula of the force field, we reversely extract the net force field using the MLFNN model. We find that the resultant F^a^ force field learned from the MLFNN model shows strong nuclear age dependence and increases as the internuclear distance increases. This is different from the common arbitrary assumption that the internuclear force is repulsive and decreasing with distance [3,12,13]. It also does not need a computational expensive screen on a variety of potential internuclear interaction types [18]. Hence, the DNN-based method demonstrated in our study could be a powerful tool in extracting the force field underlies the other complex collective behavior.

To account for the “core-border” structure of the nuclei in the particle-based model, we further add the learned attractive force field with a distance-dependent repulsive force in the short internuclear distance range. Instead of using a simple sphere surface, we run a 3D simulation on a prolate spheroid surface, which mimics the embryo shape better. The simulation results are consistent with all the observation in the experiment. Hence, this force field outperforms all the previous force fields [3,13,45], and it is the most likely force field implemented in the embryo. To understand the reason, we analyze the underlying connection between the force field and four characteristic collective behaviors of the nuclear array.

**Fig 6:**
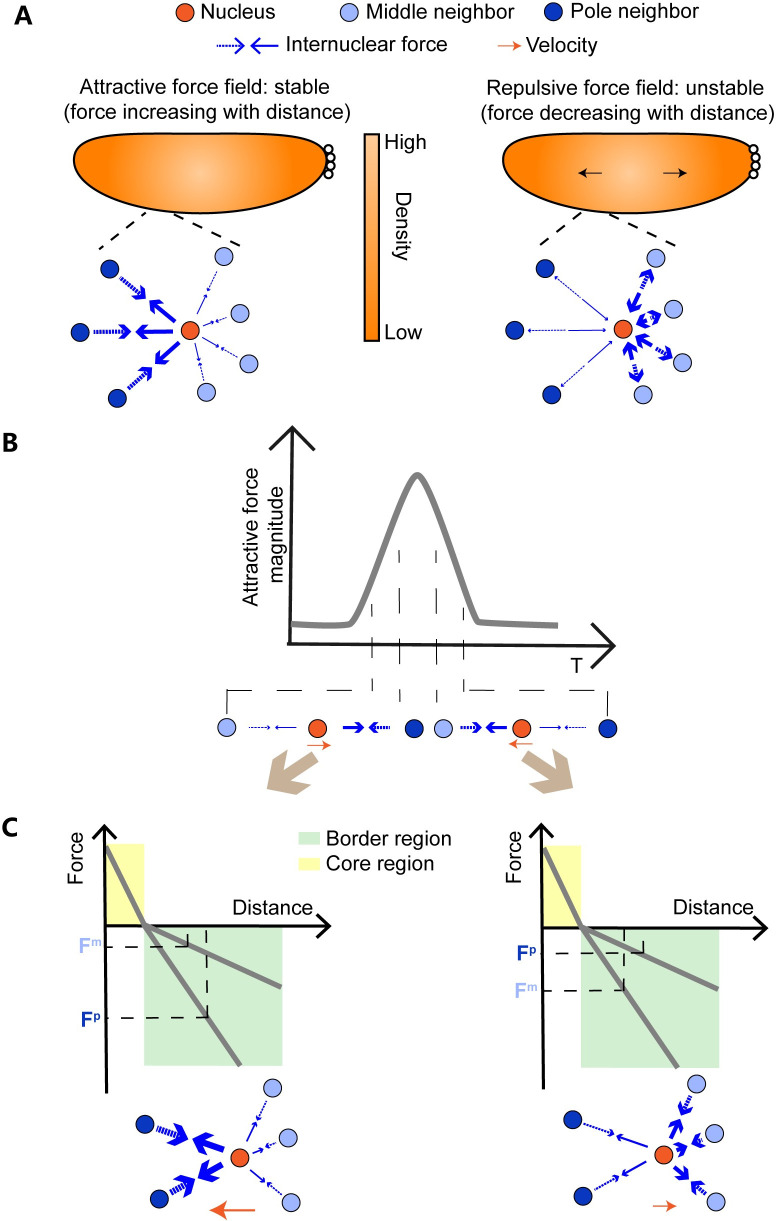
The intrinsic properties of the force fields generate the characteristic features of the collective behaviors of the nuclear array. (A) Asymmetric distribution of the nuclear density is stabilized by the attractive but not repulsive force field. As the nuclear density is high in the middle of the embryo, for a nucleus (orange circle), its nearest neighboring nuclei on the pole side (dark blue circles) are fewer in number but larger in the internuclear distance than those on the middle side (light blue circles) of the embryo. Since the attractive force increases with distance, the pair-wise force is stronger on the pole side (thicker arrows) than that on the middle side (thin arrows). Hence the net force on a given nucleus is balanced. In contrast, the repulsive force decreases with distance, the pair-wise force is stronger on the middle side, and the net force cannot be balanced. (B) Collective motion is driven by the age-dependent force field. The nuclei on the pole side (dark blue circle) have a greater nuclear age *T* than those in the middle (light blue circle). This age difference leads to a greater (weaker) attractive force from the pole side in the first (second) half period of the standing wave, hence the nuclei (orange circle) collectively move towards (away from) the pole. (C) The dampening standing wave of the nuclear speed is generated due to the greater net force in the first half period compared with the second half period. In the first half period, the amplitude of the attractive force from the pole side (F^p^) is much greater than the force from the middle side (F^m^). But in the second half period, the amplitude difference between F^p^ and F^m^ is much smaller.

Firstly, only the distance-dependent attractive force is sufficient in maintaining the non-uniform density distribution. The nuclear density is higher in the middle than in the pole region. Hence the internuclear distance *r* is smaller in the middle. For the attractive force, it increases as *r* increases. For a nucleus under the nuclear density gradient (Fig 6A), the individual pair-wise internuclear force is stronger from the embryo pole side, but the number of the interaction nuclei is more from the embryo middle side, the two factors could compensate each other to achieve a balance. In contrast, the repulsive force, which decreases as *r* increases, is always weaker from the pole side. As the nuclei close to the middle region have more neighboring nuclei with stronger repulsive force, they can push those close to the pole region (which have less neighboring nuclei with weaker repulsive force), hence the nuclei in the middle tend to move to the pole region. And we confirm that the nuclear array cannot be stabilized in the 3D simulations under the F^r^ force field in S12A Fig.

Secondly, to generate the asymmetric force driving the directional collective motion of the nuclear array along the AP axis, two factors are sufficient: the strong age dependence of the internuclear force and the age difference along the AP axis originated from the mitotic wave. Consider a nucleus in the first half period of the standing wave, the amplitude of the attractive force enforced from the embryo pole side is greater than that from the embryo middle side due to the age difference (Fig 6B), the resultant net force is towards the pole direction, hence the nuclei collectively move towards the pole. In the second half period, the amplitude of the attractive force enforced from pole side is smaller than that from the middle side, this results in a collective movement away from the pole.

Thirdly, though potentially a repulsive force field can also generate the directional collective motion (S20 Fig), only the attractive force is able to replicate one period of standing waves of the nuclear speed. In the simulation with the F^a^ force field, the internuclear force acts as an attractive force, so the newly divided nuclear regions tend to contract to pull metaphase nuclei to the poles of the embryo after *T* = 0 min. And because the attractive force can stabilize the nuclear array with a higher density distribution in the middle of the embryo, no extra motion emerges (Fig 4A and 4C). By contrast, in the simulation with the F^r^ force field, the internuclear force acts as a repulsive force, so the newly divided nuclear regions (near poles) with smaller *r* tend to expand after metaphase. This tissue expansion pushes metaphase nuclei to the middle of the embryo, which forms the extra motion process over-compressing nuclei in the middle during metaphase. And after a period of the standing wave, the higher density distribution in the middle of the embryo cannot be stabilized. The second extra motion process appears to reduce the high density in the middle region (S20A and S20C Fig).

Last, the distance-dependence of the attractive force field implemented in 3D simulations is the key to recapitulate the experimental wave peak ratio (i.e., the dampening standing wave) and partial position recovery in the simulation. Based on the F^a^ force field (Figs 3A–3C and S16A), the age-dependent attractive force increases at larger internuclear distance *r*. Since the embryo shows an initial higher nuclear density and younger nuclear age in the middle region, the resultant net effective force is asymmetric in two half periods. In the first half period, the amplitude of the attractive force from the pole side is much greater than that from the middle side as the former has greater *A(T)* and *B(r*) than the latter (Fig 6C). But in the second half period, the amplitude difference between the pole side and the middle side is smaller as the former has smaller *A(T)* but larger *B(r)* and the latter has larger *A(T)* but smaller *B(r)*. Hence the resultant force in the first half period is greater than the one in the second half.

**Fig 7:**
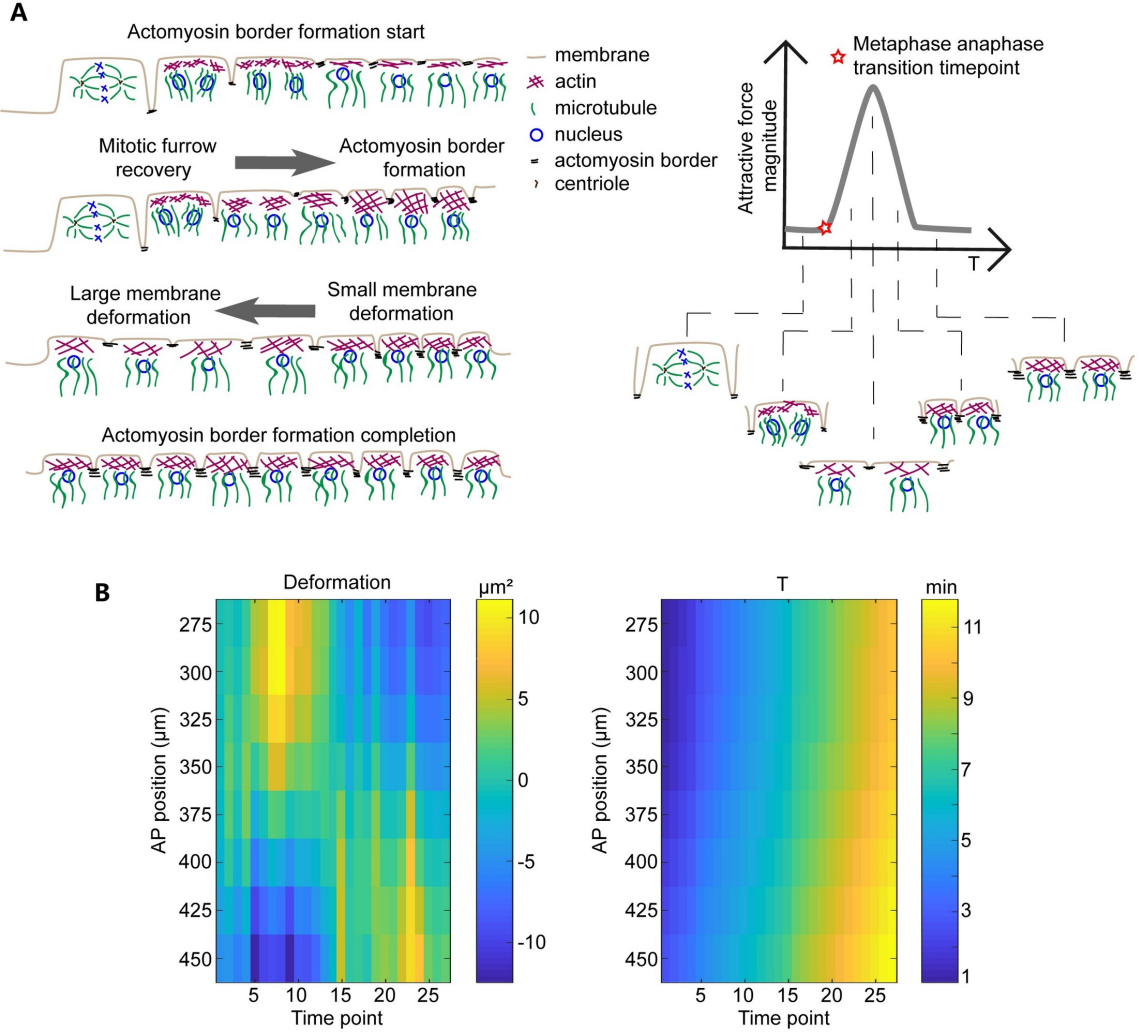
Molecular basis of the F^a^ force field from the MFLNN model. (A) The relationship between biological molecular dynamics and the F^a^ force. Each nucleus dynamically changes between five typical states during each nuclear cycle: “mitotic furrow state”, “mitotic furrow recovery state”, “flat membrane with lager membrane deformation and less myosin II state”, “actomyosin border formation with small membrane deformation state”, and “actomyosin border formation completion state”. The relationship between the states and the F^a^ force field is shown in the right panel. The corresponding nuclear motion process is shown in the left panel. (B) Heat maps of the calculated average cell area (the Voronoi area of each nucleus) variation relative to the original cell area (left) and the nuclear age (right) after the onset of anaphase 12.

However, what biological mechanism generates this force field remains to be investigated. It is clear that the force field driving the collective behavior could not be the direct effect of the mitotic spindle forces during mitosis, but the reorganization of the cytoskeleton initiated by mitosis. As the mitotic spindle force could only act for less than a minute from metaphase to anaphase, but the collective motion starts after the onset of the anaphase and lasts for ~8.8 min. Moreover, the mitotic spindle forces are isotropically oriented, whereas the collective motion is predominating along the AP axis. Based on our results and previous studies [14,22], we propose a mechanism as following. Right after metaphase, the mitotic furrow recovers and the older nuclei (close to the pole regions) forms actomyosin borders between each nucleus. The attractive force provided by the newly formed actomyosin borders pulls the tissue away from the mitotic wavefront. Note that, the metaphase region is being pulled by the anaphase region (myosin enrichment region), which is consistent with the previous study [14]. And when the M phase is finished, the younger nuclei group has a larger internuclear distance (membrane deformation) than the older nuclei group because of the previous pulling process. As a result, the tension is larger between the younger nuclei than the older nuclei. Hence, the nuclear array moves back because of the tension difference (Fig 7A). Several lines of experimental results seem to support this mechanism. For example, the computed cell deformation is larger in the middle of the embryo than the pole region during the first half period of the standing wave of the AP speed (Fig 7B). And the nuclear collective motion pattern nearly disappears after injecting myosin II inhibitor into the embryo [3], indicating that myosin II may be a core upstream factor to generate the collective motion. Further experiments such as ablation with high-spatial resolution can help to directly test this hypothesis.

The precise and reproducible packing of the nuclear array should be crucial in controlling the cell size, establishing developmental patterns, and coordinating morphogenesis in fly embryos. Previous studies [13,24] and our work show that the nuclear array packing is deviated from the closest packing on a plan, i.e., hexagonal close packing. During interphase, based on the radial distribution function data, the nuclear array has no long-range positional order, nor is amorphous [13]. And based on the KTHNY theory [35–37], during interphase the nuclear array is in the liquid state because of the topological defects (5 nuclear neighbors or 6 nuclear neighbors) [24] and exponential decay of the hexatic correlation function [12]. Moreover, the nuclear density distribution remains high in the middle of the embryo from NC11 to NC14. On the one hand, such a distribution is robust and it could be maintained as long as the collective nuclear motion is generated based on the F^a^ assumption. On the other hand, such a density distribution may also help to maintain the collective motion. Assuming the mitotic wave is triggered at the locations with a low nuclear density [46], the mitotic wave will always start from the two poles and move to the middle of the embryo. The nuclear age difference along the AP axis leads to the asymmetric force driving the collective nuclear motion along the AP axis. This density distribution could be generated by the self-organized nuclear spreading from the middle to the whole embryo during the pre-blastoderm [2]. Hence the nuclear packing pattern and the collective motion pattern consist a self-sustainable feedback loop. The biological significance of these collective behaviors of the nuclear array remains to be explored in future studies.

## Materials and methods

### 4D live imaging of early *Drosophila* embryos

#### *Drosophila* embryo sample preparation

The fly stock was maintained at 25°C on cornmeal medium. H2Av-GFP fly line (from Thomas Gregor Lab at Princeton University) embryos were collected in 1.5 hours on the grape juice plate, then dechorionated in 4% NaClO bleach buffer for 2 min. After being washed in ddH_2_O for several times, the embryos were mounted in 1% low melting agarose in the capillary. For the convenience of imaging, the embryos were carefully mounted along the AP axis and the light sheet can transmit from the dorsal or ventral side of the embryo.

#### Light sheet microscope imaging

Imaging experiments were performed with a Zeiss Light Sheet Z.1 Microscope. The illumination objective is LSFM 10×/0.2, and the detection objective is W Plan-Apochromat 20x/1.0 Corr DIC M27 75 mm. Under DIC imaging, the pole cell formation process can be clearly identified, which is a marker for the start of NC10. Starting from NC10, the imaging lasts for about 2 hours to cover four mitotic phases from NC10 to NC14. H2Av-GFP is excited by a 488 nm laser with 1~2% laser intensity and the exposure time of 30 ms to avoid high phototoxicity which may cause nuclei falling to the yolk [24]. The emission light from 505 nm to 545 nm was collected. To study the collective motion pattern, a relatively high imaging speed is needed. The *z* stack images (1920×1920 pixels, pixel size 0.286 μm) were acquired in 1 μm steps at 20 s time intervals. To achieve high temporal resolution, the embryos were only imaged from one angle, so that three quarters of the embryos were captured. “Dual Side when Experiment” mode and “Pivot Scan Settings” mode were selected while imaging to achieve dual side illumination and reduce shadows which might be cast by optically dense structures within the sample.

The protocols from the ref. [30] was applied in the nuclear segmentation process.

### Data analysis

#### Projected nuclear speed, trajectory and density along the AP axis

After nucleus segmentation process, the 4D nuclear position information was obtained. At each time point, the nuclear coordinates are a collection of data points on the embryo surface, i.e., a point cloud. The middle points of scattered nuclear point cloud in the anterior end and posterior end were manually picked as the anterior pole and posterior pole.

The nuclear position *x*(*t*) was projected to the AP axis to obtain the AP projected trajectory. The nuclear speed was calculated according to the formula *v*(*t*) = (*x*(*t*)−*x*(*t*−Δ*t*))/Δ*t*. Here Δ*t* is the imaging time interval, i.e., 20 s. For plotting the heatmap of the nuclear speed, in each time point the nuclear speed was averaged in each bin with the width of ~5% EL along the AP axis.

The embryo surface was reconstructed from the scattered cell nuclear point cloud with the function MyCrustOpen.m (from MathWorks File Exchange website: https://ww2.mathworks.cn/matlabcentral/fileexchange/63731-surface-reconstruction-from-scattered-points-cloud-open-surfaces). After reconstruction, the point cloud was triangulated. Using Voronoi diagram, each triangular facet can be divided into three quadrangles. Each quadrangle area contains a triangle vertex (a nucleus). The local nuclear density was obtained by taking the inverse of all the quadrangle area. For plotting the heat map of the nuclear density, in each time point the nuclear density was averaged in each bin with the width of 10% EL along the AP axis.

#### Order parameters

The most efficient and the closest packing on the plane is hexagonal close-packing. To evaluate the regularity of the nuclear array in the *Drosophila* embryo, the hexatic bond-orientational order parameter is calculated with the formula $\varphi_{array}=\frac{1}{N}\sum_{j=1}^{N}\left|\frac{1}{n(j)}\sum_{i=1}^{n(j)}exp\;(i6{\hat{\theta }}_{i})\right|$ [24]. *N* is the total number of the nuclei, *n* is the neighbor number of each nucleus and $\hat{\theta }$ is the angle between vertical vector and the vector between each nucleus (*i*^th^ nucleus) and its neighbor nucleus (*j*^th^ nucleus) [24]. The value of *ψ*_*array*_ varies from 0 to 1. The larger the value, the closer the nuclear array to the hexagonal closed packing array.

To evaluate the degree of nuclear array motion collectivity during the developmental process in the *Drosophila* embryo, the order parameter in the field of collective motion defined by the equation $\varphi_{speed}^{\prime }=\frac{1}{Nv_{0}}|\sum_{i=1}^{N}{\vec{v}}_{i}|$ is used [4]. *N* is the total nuclear number, *v*_0_ is the average absolute nuclear velocity and ${\vec{v}}_{i}$ is the velocity of each nucleus. The value of $\varphi_{speed}^{\prime }$ also varies from 0 to 1. If the motion is disordered, the velocities of each nucleus point to random directions and average out to give a small magnitude vector, whereas for ordered motion the velocities add up to a vector of absolute velocity close to *Nv*_0_. To account for the potential effect of the velocity difference, the equation is amended to $\varphi_{speed}=\frac{1}{Nv_{0}}|\sum_{i=1}^{N}{\vec{v}}_{i}|*\frac{v_{0}}{v_{max}}$. Here *v*_*max*_ is the maximal velocity of all the nuclei in all time points.

### Learn the internuclear force field function from data via a multi-layer feedforward neural network

#### One dimensional nuclear array motion dataset

In the 1D condition, a nuclear array unit instead of a single nucleus is used as a motion unit. Each nuclear array unit represents all the nuclei within a bin of ~5% EL at a given AP position. Its corresponding density, age and speed are obtained by smooth spline fitting to the average value of all the nuclei inside the bin (S8C Fig). Note that, the two data points in the embryo poles in each time point are considered to be fixed and not included in the dataset.

All the input data including nuclear density data and nuclear age data are normalized by the equation: $\frac{x_{i}*n}{x_{max}-x_{min}}$. Here, *n* equals 100. After normalization, the density and age datasets have similar data range, which ensures that the age dataset and density dataset have similar contribution for training the DNN and helps to accelerate the speed for searching the optimal solution via a gradient descent method.

#### Nuclear age assignment

To account for the mitotic wave starting from the two poles, each nucleus is assigned with a nuclear age *T*. *T* = 0 for all the nuclei at the mitotic wavefront, where they reach the transition time between the metaphase and anaphase. The nuclei away from the mitotic wave starting points approach *T* = 0 at a later time than those close to the starting points. The different time points at which the nuclei at different AP positions approach *T* = 0 can be identified from the density gradient data as shown in S23 Fig. As the new mitotic wave moves from the two poles to the embryo middle, the nuclei before and after the mitotic wavefront are at different nuclear cycles transiently. To avoid miscalculating the average nuclear age of the neighboring nuclear array units, the nuclear age of the nuclei at the AP position before the mitotic wave reaches is temporarily converted to negative values (time before reach *T* = 0) in the new nuclear cycle, instead of the accumulated nuclear age from the former nuclear cycle.

#### Nuclear motion model

Following the same approach of previous work based on an empirical assumption [3,12,13,18,38–40], we implement the overdamped equation as the nuclear motion model to extract the internuclear force from the nuclear movement at the single nucleus level. As the Reynolds number (given the typical value for the speed of nuclear motion is 0.13 μm/s, the minor-axis of the embryo is 150 μm, and the kinematic viscosity of water is 3∙10^6^ μm^2^/s, the Reynolds number is approximately 6.5∙10^−6^.) is really small inside the embryo, the inertia force is omitted, nuclear motion is dominated by viscous forces, and the nuclear velocity times the friction coefficient *γ* should equal to the resultant internuclear force. Hence, the discretization version of the overdamped equation in three dimensions is,

$$\sum_{j\in n(i)}{\vec{f}}_{i,j}=\gamma {\vec{v}}_{i},$$ (1)

where ${\vec{f}}_{i,j}$ is net effective force between nucleus i and j (only the neighboring nuclei of nucleus i are summered up), and ${\vec{v}}_{i}$ is the velocity of nucleus i. Based on Stokes’ law, *γ* = 6*πηa*, and *η* is the viscosity coefficient.

Because the collective motion pattern and packing pattern of the nuclear array are predominant along the AP axis (Figs 2 and S7), the 3D single nuclear data is converted to a 1D dataset consisting of a list of nuclear array units, in which the nuclear speed, density, and age are averaged in a bin of 5% EL along the AP axis (S8A and S8C Fig). This data preprocessing eliminates the single nuclear data noise and captures the main features of the dataset, which is helpful for the subsequent deep learning process. Correspondingly, the overdamped equation in 3D (Eq (1)) is further reduced to 1D (Eq (2)). Here, a nuclear array unit instead of a single nuclear is considered as a unit of the system (S8A Fig). Because the pairwise internuclear interaction number between two adjacent nuclear array units is positively correlated with the average nuclear density (S8A Fig), we multiply the average pairwise internuclear force ${\vec{F}}_{i,j}$ with the average nuclear density ${\bar{\rho }}_{i,j}$ of the neighboring i^th^ and j^th^ nuclear array unit (i.e., ${\bar{\rho }}_{i,j}=(\rho_{i}+\rho_{j})/2$) and sum them up in the 1D case to approximate the resultant force in the 3D case:

$$\sum_{j\in n(i)}{\bar{\rho }}_{i,j}{\vec{F}}_{i,j}=\tilde{\gamma }{\vec{V}}_{i},$$ (2)

where $\tilde{\gamma }$ is the effective friction coefficient and ${\vec{V}}_{i}$ is the average velocity of the nuclei in the *i*^*th*^ nuclear array unit. Note that, *ρ*_*i*_ describes the average nuclear density of all nuclei in the *i*^*th*^ nucleus array unit. And the nuclear density *ρ* for a single nucleus is defined as the reciprocal of the Voronoi area (*s*) of the nucleus: $\rho =\frac{1}{s}$.

We assume that the magnitude of ${\vec{F}}_{i,j}$ depends on the nuclear density *ρ* and the nuclear age *T*. It is convenient to learn the function form of *F*_*i*,*j*_ (the magnitude of ${\vec{F}}_{ij}$) from the 1D nuclear array motion dataset via a classical multilayer feedforward network (MLFNN) [27,28] model following the equation (S8B Fig):

$$F_{i,j}^{learned}(\tau_{i},\tau_{j},\rho_{i},\rho_{j})=\underset{m\in \{\varphi :R^{2}\to R\}}{m({\bar{\rho }}_{i,j},{\bar{\tau }}_{i,j})},$$ (3)

where *m* is an arbitrary function (*R* is the set of real number and *φ* is a map from *R*^2^ to *R*), which has two independent variables ${\bar{\rho }}_{i,j}$ and ${\bar{\tau }}_{i,j}$ (S8B Fig). The function *m* can be approximated by a MLFNN with two input nodes and one output node using the softplus function (*f*(*x*) = *log*(*e*^*x*^+1)) as the activation function except for the output layer (S8B Fig, for the architecture of the MLFNN, see S2 Text). ${\bar{\tau }}_{i,j}$ are the average age of the *i*^*th*^ and *j*^*th*^ nuclear array unit respectively (i.e., ${\bar{\tau }}_{i,j}=\tau_{i}+\tau_{j})/2$). Using ${\bar{\rho }}_{i,j}$ and ${\bar{\tau }}_{i,j}$ as the input of the MLFNN can guarantee the interaction force between the two units is equal in the force magnitude and opposite in direction (S8A Fig left panel, for more details of the internuclear force formula, see S3 Text). The output of the MLFNN is the magnitude of the average pairwise internuclear force between the *i*^*th*^ and *j*^*th*^ nuclear array unit ($F_{i,j}^{learned}$).

Since *F*_*i*,*j*_ could be either attractive or repulsive, we take both possibilities into consideration. In brief, we call the attractive force field as F^a^ and the repulsive force field as F^r^. We define the direction towards the posterior pole to be the positive direction of the force. Based on the F^a^ assumption, for a given nuclear array unit *i*, the unit vectors of the force orientation from the anterior and posterior nearest neighbors are ${\vec{e}}_{i,i-1}=[-1]\;and\;{\vec{e}}_{i,i+1}=[1]$, respectively (S8A Fig). By definition, ${\vec{e}}_{i,j}=\frac{{\vec{x}}_{j}-{\vec{x}}_{i}}{\left|{\vec{x}}_{j}-{\vec{x}}_{i}\right|}$ (*j* = *i*−1, *i*+1), and *x* is the mass center position of the nuclear array unit along the AP axis. As for the F^r^ assumption, ${\vec{e}}_{i,j}=\frac{{\vec{x}}_{i}-{\vec{x}}_{j}}{\left|{\vec{x}}_{i}-{\vec{x}}_{j}\right|}$, hence ${\vec{e}}_{i,i-1}=[1]\;\mathrm{and}\;{\vec{e}}_{i,i+1}=[-1]$ (S8A Fig). Assume the internuclear force is additive, so the resultant force learned from DNNs is (S8B Fig):

$${\vec{F}}_{i}^{learned}=\sum_{j\in n(i)}F_{i,j}^{learned}{\bar{\rho }}_{i,j}{\vec{e}}_{i,j}$$ (4)

Based on the overdamped assumption, the ground truth of the resultant force is proportional to the motion speed (S8B Fig):

$${\overset{⃑}{F}}_{i}^{data}=\tilde{\gamma }{\overset{⃑}{V}}_{i}^{data}$$ (5)

We search the best MLFNN model (m*) that fits the training data set (S8B Fig):

$$loss=\sum_{i=1}^{n}{\left\|{\vec{F}}_{i}^{learned}-{\vec{F}}_{i}^{data}\right\|}_{2}$$ (6)

$$m^{*}=\underset{m\in \{\varphi :R^{2}\to R\}}{\mathrm{argmin}}\left(loss\right)$$ (7)

The training converges very quickly via the conventional back-propagation algorithm within 2000 training steps (S9 Fig). For the details of DNN architecture, loss function and training see S2 Text.

### The particle-based model to simulate nucleus array collective motion pattern on the prolate spheroid surface

#### The basic setup

The embryo is modeled as a prolate spheroid. Consistent with the embryo size, the ratio of the ellipsoid’s major axis and minor axis is 5:1.5 [32]. Each nucleus is represented as a particle on the prolate spheroid surface. While simulation, the initial nuclear number is 400. After mitosis, the nuclear number doubles. The nuclear density of the simulation is comparable with the nuclear density in the interphase from NC10 to NC11. The neighbors of a nucleus are identified via the Voronoi tessellation.

In the prolate-spheroidal coordinate system, the initial nuclear coordinates (*ν*,*θ*,*φ*) are set as the following: *ν* is a constant, *θ* is sampled from Gaussian distribution, and *φ* is sampled from the Uniform distribution. Because after nuclei migrating from yolk to the cortex region during the interphase of NC10, the initial nuclear density is higher in the middle than the pole regions in the embryo [2], it is reasonable to use Gaussian distribution of *θ* to imitate this process. Then the random nuclear coordinates are rearranged under the distance dependent force fields to form a new nuclear array as the initial state. For the equations of motion in polar coordinates on the prolate spheroid surface, see S7 Text.

#### The force fields

To approximate the force field from the MLFNN model, we simply assume the magnitude of the force field is the multiplication of the age-dependent force *A(T)* and the distance dependent force *B(r*). To calculate the pairwise internuclar force in simulations, *T* is the average age of the adjacent nuclei (${\bar{\tau }}_{i,j}=(\tau_{i}+\tau_{j})/2$) and *r* is the internuclear distance between the adjacent nuclei (${\bar{r}}_{i,j}=|{\vec{r}}_{i}-{\vec{r}}_{i}|$).

Under the F^a^ assumption, the attractive force field is defined as:

$$F_{i,j}(\tau_{i,}\tau_{j,}{\vec{r}}_{i,}{\vec{r}}_{j})=\left\{ \begin{array}{c} A({\bar{\tau }}_{i,j})B({\bar{r}}_{i,j})\;{\bar{r}}_{i,j}\geq r_{0}\\ B({\bar{r}}_{i,j})\;{\bar{r}}_{i,j}<r_{0} \end{array}\right.$$ (8)

$$A({\bar{\tau }}_{i,j})=\left\{ \begin{array}{c} F_{1}-(\cos(\frac{{\bar{\tau }}_{i,j}}{t_{1}}\pi )+1)(\frac{F_{1}-F_{2}}{2})\;(0\leq {\bar{\tau }}_{i,j}<2t_{1})\\ F_{2}\;({\bar{\tau }}_{i,j}\geq 2t_{1}) \end{array}\right.$$ (9)

$$B({\bar{r}}_{i,j})=F_{0}\left(1-\frac{{\bar{r}}_{i,j}}{r_{0}}\right)$$ (10)

The *F*^*a*^ force field is shown in S16A Fig, and the five free parameters used are listed on S1 Table: *t*_1_ = 0.23; *F*_1_ = 1; *F*_2_ = 0.2; *r*_0_ = 8.5; *F*_0_ = 15. The nuclear cycle time is set as 1. For other force fields, see S8 Text.

#### Mitosis

In wild type embryos, mitotic waves usually start from the anterior pole and posterior pole [42,47]. Each nucleus is assigned with a corresponding nuclear age according to its AP position. The initial nuclear age distribution and the subsequent evolution along the AP axis are shown in S16C Fig. As the nuclear age approaches the total nuclear cycle time, a mother nucleus is divided to be two daughter nuclei and the age of new nuclei is set to zero.

Take the similar approach in previous studies [3,45], the two daughter nuclei are separated with a randomly selected small internuclear distance upon division, and their mass center is set at the original position of the mother nucleus. More specifically, in the prolate-spheroidal coordinate system, the angle variation of each daughter nucleus relative to the mother nucleus (∆θ or ∆φ) is randomly chosen from -5×10^−4^ to 5×10^−4^ (S1 Table). With this method, we can assure a random nuclear division orientation [3], and keep the internuclear distance between daughter cells less than 0.1 μm. This distance is within the core region of the distance-dependent force field, so the subsequent separation of daughter nuclei is driven by the repulsive force in the force field (no additional “nuclear division repulsive force” is need).

## Supporting information

S1 Text Temperature-dependent scaling of the embryo developmental time. (DOCX)

S2 Text DNN architecture, loss function and Training. (DOCX)

S3 Text The choice of the internuclear force formula. (DOCX)

S4 Text Ruling out the repulsive force field from the DNN learning results. (DOCX)

S5 Text Evaluate the ground truth recovery by the DNN. (DOCX)

S6 Text Deterministic mean-field physical model. (DOCX)

S7 Text Equations of motion in polar coordinates on the prolate spheroid surface. (DOCX)

S8 Text The force fields used in the simulations. (DOCX)

S1 Table Parameters used in the simulations. (DOCX)

S1 Code The code used for the DNN learning. (RAR)

S2 Code The code used for 3D simulations using the particle-based model. (RAR)

S1 Data The original data associated with figures. (ZIP)

S1 Fig Illustration of the nuclear density at interphase 13. (A) The maximum intensity projection of a z stack of the light sheet images. The embryo is expressing H2Av-GFP and the imaging time is a time point at interphase 13 when the nuclear array is stable. The three equal sized red squares mark three regions in the anterior, middle and posterior of the embryo. (B) Trisurf plot of the point cloud data of the nuclear position in A. The color of each triangle vertex illustrates the density of each nucleus. Note that, the nuclear density is calculated by the reciprocal of Voronoi area of each nucleus.
(TIF)

S2 Fig Nuclear density distribution along the AP axis. Heat maps of the nuclear density projected along the AP axis in an embryo imaged at 19.1°C (A), 20.6°C (B), 24.4°C (C), and 21.3°C (D)). The AED time was rescaled at 25°C (see S1 Text).
(TIF)

S3 Fig Dynamics of hexatic bond-orientational order parameter of four embryos. The black arrows 1, 2 and 3 label the time points of the minimum order around M phase 11, 12, and 13, respectively. The arrow 4 labels the starting time point of gastrulation, after which the nuclear array order drops dramatically. The imaging temperature of the four embryos is estimated to be 19.1°C, 20.6°C, 24.4°C, and 21.3°C respectively, and the AED time was rescaled at 25°C (see S1 Text).
(TIF)

S4 Fig The relationship between the time of the density changing and the collective motion with standing waves of the AP speed. Boxplots (whisker, min/max values, boxes, 25/75 percentiles). The median of the density changing time and wave time is 4.9 min and 8.8 min, respectively, in four embryos. The density changing time is during the middle period of the wave time, in which the nuclear speed is relatively high.
(TIF)

S5 Fig The nuclear AP trajectory of four embryos. The nuclear trajectories were projected to the AP axis of the embryo. Yellow lines highlight the typical trajectories among all nuclear trajectories in each embryo. The imaging temperature of the four embryos was estimated to be 19.1°C, 20.6°C, 24.4°C, and 21.3°C, respectively. The AED time was rescaled at 25°C (see S1 Text).
(TIF)

S6 Fig Dynamics of the order parameter of collective motion of the nuclear array. The order parameter of all the nuclei in each bin (for more details, see Materials and methods). APi corresponds to the *i*^*th*^ bin with the width of 10% EL from the anterior pole. The markers 1, 2 and 3 label three time intervals that show high motion collectivity in (A) and (B). The AED time was rescaled at 25°C (see S1 Text).
(TIF)

S7 Fig Dynamics of the nuclear speed projected along the DV axis. (A) The mean (line) and SD (shadow) of the nuclear speed projected along the DV axis as a function of the developmental time AED. (B) Heat map of the nuclear speed projected along the DV axis. The AED time was rescaled at 25°C (see S1 Text).
(TIF)

S8 FigLearning the force field functions from 1D data via the MLFNN model.(A) The *i*^*th*^ nuclear array unit only interacts with its nearest neighbors (the *j*^*th*^ units, where *j* = *i*-1 and *i*+1). Based on F^a^ (or F^r^) assumption, the orientation of the pairwise internuclear force ${\vec{e}}_{i,j}$ (black arrow) are different. The average nuclear density (${\bar{\rho }}_{i,j}$) and average nuclear age (${\bar{\tau }}_{i,j}$) are the mean of the values of the adjacent nuclear array units. The pairwise internuclear interaction number (dashed line) is positively correlated with ${\bar{\rho }}_{i,j}$. (B) The pairwise internuclear force ($F_{i,j}^{learned}$) is the function of ${\bar{\rho }}_{i,j}$ and ${\bar{\tau }}_{i,j}$. And $F_{i,j}^{learned}({\bar{\rho }}_{i,j},{\bar{\tau }}_{i,j})$ can be approximated by a three-layer MLFNN. The resultant force of a nuclear array unit ${\vec{F}}_{i}^{learned}$ is calculated by adding the pairwise internuclear force $F_{i,j}^{learned}{\vec{e}}_{i,j}$ from the adjacent units of the *i*^*th*^ nuclear array unit (${\vec{F}}_{i}^{learned}=\sum_{j\in n(i)}F_{i,j}^{learned}{\bar{\rho }}_{i,j}{\vec{e}}_{i,j}$). Then the learned resultant force (${\vec{F}}_{i}^{learned}$) is compared to the ground truth (${\overset{⃑}{F}}_{i}^{data}=\tilde{\gamma }{\overset{⃑}{V}}_{i}^{data}$) to define the loss function ($loss=\sum_{i=1}^{n}||{\vec{F}}_{i}^{learned}-{\vec{F}}_{i}^{data}|{|}_{2}$) for training. Here only the first item of the loss function is shown. For more details of the training, see S2 Text. (C) Snapshots of the training dataset. All the data are discretized along the AP axis (see Materials and methods).(TIF)

S9 Fig Loss function. The loss functions while training the DNN. The data from M phase 13 to interphase 14 in one embryo (3078 data points in total) is used while training. The training is based on two interaction assumptions: net effective repulsive force (F^r^) (A) and net effective attractive force (F^a^) (B).
(TIF)

S10 FigForce field functions learned from 1D data via the MLFNN model.The DNN learning results are based on the F^a^ assumption. (A-C) The data from M phase 11 to interphase 12 in one embryo (2394 data points in total) is used while training the DNN. (A) A representative heat map of the function *F*(*T*,*r*). Here *F* is the magnitude of the internuclear force ($F_{i,j}^{learned}$), *T* is the nuclear age after the onset of anaphase and *r* is the internuclear distance. Note that, $r=\sqrt{s}$ and $\rho =\frac{1}{s}$, here *s* is the Voronoi area of the nuclei. (B) *F* has a positive correlation with *r* as *T* = 1.9–3.8 min. (C) *F* has a conservative pulsatile relationship with *T* as *r* = 18.3–20.8 μm. (D-F) The DNN learning results as in A-C. The data from M phase 12 to interphase 13 in one embryo (3002 data points in total) is used while training the DNN. (E) *T* = 1.9–3.2 min. (F) *r* = 10–10.5 μm.(TIF)

S11 Fig The force field function is conservative in different training trials under the F^a^ assumption. The data from M phase 13 to interphase 14 in one embryo (3078 data points in total) is used while training the DNN. Three randomly selected DNN learning results are shown in A-C.
(TIF)

S12 Fig Nuclear array distribution during interphase on the ellipsoid surface in 3D simulation. (A) If the magnitude of the repulsive internuclear force (*F*) linearly increases with the internuclear distance (*r*), no stable nuclear array can be generated during interphase. The nuclei form aggregations on the ellipsoid surface. (B) If the magnitude of the repulsive force *F* linearly decreases with *r*, a stable nuclear array can be generated during interphase. For simulation details, see Materials and methods.
(TIF)

S13 Fig Force field functions learned from simulation data via the MLFNN model is consistent with the ground truth force field functions used in the simulation. (A-B) Comparison between the learned force field (A) and the ground truth force field used in the simulation in Fig 4 (B) for the attractive force. (C-D) Comparison between the learned force field (C) and the ground truth force field used in the simulation in S20 Fig (D) for the repulsive force.
(TIF)

S14 FigForce field functions learned from 1D data via the MLFNN model without density correction.The DNN learning results are based on the F^a^ assumption. But the resultant force ${\vec{F}}_{i}^{learned}$ is calculated by adding the resultant force $F_{i,j}^{learned}{\vec{e}}_{i,j}$ from the neighboring units of the *i*^th^ nuclear array unit (${\vec{F}}_{i}^{learned}=\sum_{j\in n(i)}F_{i,j}^{learned}{\vec{e}}_{i,j}$). (A-C) The data from M phase 11 to interphase 12 in one embryo (2394 data points in total) is used while training the DNN. (A) A representative heat map of the function *F*(*T*,*r*). (B) *F* is independent on *r* as *T* = 1.9–3.8 min. (C) *F* has a conservative pulsatile relationship with *T* as *r* = 18.3–20.8 μm. (D-F) The DNN learning results as in A-C. The data from M phase 12 to interphase 13 in one embryo (3002 data points in total) is used while training the DNN. (E) *T* = 1.9–3.2 min. (F) *r* = 10–10.5 μm. (G-I) The data from M phase 13 to interphase 14 in one embryo (3078 data points in total) is used while training the DNN. (H) *T* = 4.4–5.6 min. (I) *r* = 7.9–8.2 μm.(TIF)

S15 FigThe calculation of the equation of state *p* = *p*(*ρ*,*τ*).(A) Integration of the momentum equation $\gamma v(x,t)=-\frac{\partial }{\partial x}p(x,t)$ in two different time points results in two range in the *ρ*−*τ* plane, *R*_0_ and *R*_1_. (B) By integrating $\gamma v(x,t)=-\frac{\partial }{\partial x}p(x,t)$ at different time points, the equation of state emerged from the resulted range in the *ρ*−*τ* plane. (C) Formulating the “negative pulse shape” *p*−*τ* curve. Error bar is the binned standard deviation of the result. (D) The effective pressure is mainly determined by the age while the effect of density is subtle.(TIF)

S16 Fig The force field and nuclear age distribution along the AP axis used in simulations on the prolate spheroid surface (see Materials and methods and S5 Text). (A, B) The green squares show the border regions in which the distance dependent force function multiplies with the time dependent force function. The yellow squares show the core regions of the distance dependent force. The attractive force field and repulsive force field are shown in the heat maps. Note that, attractive direction is the positive direction and repulsive direction is the negative direction. (C) Mitotic waves start from the anterior pole and posterior pole of the embryo (gray stars indicate the mitotic wave start point), so that the nuclear age has phase difference along the AP axis. Heat map shows the average nuclear age along the AP axis in each simulation step.
(TIF)

S17 Fig Nuclear array distribution during the interphase in 3D simulations using the F^a^ force assumption without the core region. (A) The force field used in the simulation. (B) The nuclear array distribution during the interphase with a very high nuclear density in the middle and a very low nuclear density in the poles.
(TIF)

S18 Fig 3D simulation results based on the F^a^ force assumption (the nuclear number doubling from 800 to 1600). (A) Heat map of the nuclear density projected along the AP axis. (E) Boxplots (whisker, min/max values, boxes, 25/75 percentiles). The medians (red line) of measured and simulated density ratio are 0.58, 0.65, 0.78, 0.53 and 0.64, respectively. Density ratio is defined as the ratio between the anterior (~5–15% EL) or posterior (~85–95% EL) density and the maximal density in the middle of the embryo during interphase. The force field used in this simulation is shown in S16A Fig.
(TIF)

S19 Fig Nuclear trajectories along the AP axis in 3D simulations are consistent with the experimental data. (A) Nuclear trajectories of 3D simulations in Fig 4. (B) Nuclear trajectories of experimental data in Fig 2 during interphase 13. (C) The maximum nuclear displacement along the AP axis in 3D simulations with different mitotic wave durations. The labels “*%NC” indicate the mitotic wave duration, e.g., 10%NC indicate the mitotic wave duration is 10% nuclear cycle time. The mitotic wave speed ratio of the three mitotic wave durations is 6(10%NC): 4(15%NC): 3(20%NC). Dashed lines are the original data and full lines are the fitted curves. The maximum displacement simulation data is consistent with the maximum displacement experimental data in ref [3]. The force field used in these simulations is shown in S16A Fig.
(TIF)

S20 Fig 3D simulation results based on the F^r^ force field (for the nuclear movement in the whole embryo see S5 Movie). The characteristic features of the collective motion pattern and packing pattern of the nuclear array plots as in Fig 4A–4D. The force field used in this simulation is shown in S16B Fig. Black triangles in (C) mark two extra motion processes along the AP axis during simulation comparing to the experimental data.
(TIF)

S21 Fig 3D simulation results based on the F^a^ force field with a different *B(r)* function (for the nuclear movement in the whole embryo see S6 Movie). The characteristic features of the collective motion pattern and packing pattern of the nuclear array plots as in Fig 4A–4D. The internuclear force (*F*) has quadratic function relationship with the internuclear distance (*r*) instead of the linear relationship in Fig 4 (see S5 Text).
(TIF)

S22 Fig Simulation results of the collective motion pattern of the extreme cases. The force field used in this simulation is shown in S16A Fig. (A) The mitotic wave starts from one pole of the embryo. (B) The mitotic wave starts from a quarter point and three quarters point of the embryo. Gray stars indicate the mitotic wave start time point. The figures below are the corresponding heat maps of the AP speed, the AP nuclear density and the dynamics of hexatic bond-orientational order parameter.
(TIF)

S23 Fig Find start points of the nuclear cycle. The density gradients (B) were calculated from one dimensional density data (A). The maximum density gradient values around the mitotic phase were identified (red lines in C) and fitted with a smooth spline (blue line in C). The corresponding time points indicating the metaphase anaphase transition time points in each nuclear cycle are labeled in A as white dots.
(TIF)

S1 Movie Nuclear segmentation and tracking based on TGMM algorithm. (MP4)

S2 Movie The standing wave of nuclear speed projected along the AP axis. (MP4)

S3 Movie The nuclear speed projected along the DV axis. (MP4)

S4 Movie The particle-based model simulation based on the F^a^ assumption (the internuclear force (*F*) has a linear relationship with the internuclear distance (*r*)). Red and green represent the left (anterior) and right (posterior) direction, respectively. And the intensity indicates the magnitude of the nuclear velocity.
(MP4)

S5 Movie The particle-based model simulation based on the F^a^ assumption (The internuclear force (*F*) has a quadratic relationship with the internuclear distance (*r*)). Red and green represent the left (anterior) and right (posterior) direction, respectively. And the intensity indicates the magnitude of the nuclear velocity.
(MP4)

S6 Movie The particle-based model simulation based on the F^r^ assumption. Red and green represent the left (anterior) and right (posterior) direction, respectively. And the intensity indicates the magnitude of the nuclear velocity.
(MP4)
