# Supplementary material for: Attractive internuclear force drives the collective behavior of nuclear arrays in Drosophila embryos
Source: PLoS Comput Biol. 2021 Nov 19;17(11):e1009605. doi: 10.1371/journal.pcbi.1009605 (PMC8641897; doi:10.1371/journal.pcbi.1009605)
Supplement: S1 Text — (DOCX) [file pcbi.1009605.s001.docx]

**S1 Text. Temperature-dependent scaling of the embryo developmental time**

*Drosophila* embryos follow reproducible developmental time ($t_{dev}$) under specific temperature (*T*). At 25℃, $t_{dev}$of NC10, NC11, NC12, and NC13 are 9 min, 10 min, 12 min, and 21 min, respectively, according to the somatic bud formation and collapse process at the anterior tip [1].

The developmental time from AED (after embryo deposition) to the end of NC13 is 130 min and to the end of cellularization is 180 min. And the developmental time from AED to the end of NC11 is 97 min and to the end of NC12 is 109 min.

Besides, the proportional developmental time scales uniformly across temperature (from 17.5℃ to 32.5℃) in diverse *Drosophila* species. Between 17.5℃ and 27.5℃, the relationship between $t_{dev}$ (total developmental time) and *T* can be fitted by an exponential regression $t_{dev}=e^{\frac{\alpha}{T}}$. As for *Drosophila melanogaster*, the function is $t_{Dmel}=4.02e^{37.31/T}$ [2].

According to our data, the nuclear speed increases dramatically during metaphase. Hence the nuclear cycle starting time point is identified as the time point at which the standard deviation of the AP speed is maximal during the mitotic phase at the anterior tip. The nuclear cycle time was calculated by the difference of two nuclear cycle starting time points. To estimate the developmental time (AED) of the embryos ($t_{AED}^{exp}$), one can use the equations:

$$\begin{aligned} {t_{nc1nc14}^{exp}=t}_{nc12}^{exp}\div\frac{t_{nc12}^{25}}{t_{nc1nc14}^{25}}\#\left( AUTONUM \backslash* Arabic \right) \end{aligned}$$

$$\begin{aligned} {t_{nc1nc12}^{exp}=t}_{nc1nc14}^{exp}\times\frac{t_{nc1nc12}^{25}}{t_{nc1nc14}^{25}}\#\left( AUTONUM \backslash* Arabic \right) \end{aligned}$$

$$\begin{aligned} t_{AED}^{exp}=t^{exp}-t_{nc12start}^{exp}+t_{nc1nc12}^{exp}\#\left( AUTONUM \backslash* Arabic \right) \end{aligned}$$

The developmental time of the embryos at the other temperatures can be scaled to 25℃,

$$\begin{aligned} t_{AED}^{exp25}=t_{AED}^{exp}\times\frac{t_{nc1nc14}^{25}}{t_{nc1nc14}^{exp}}\#\left( AUTONUM \backslash* Arabic \right) \end{aligned}$$

To estimate the developmental temperature ($T_{exp}$) of the embyos, one can follow the equations:

$$\begin{aligned} t_{Dmel}^{25}=4.02e^{\frac{37.31}{25}}\#\left( AUTONUM \backslash* Arabic \right) \end{aligned}$$

$$\begin{aligned} t_{Dmel}^{exp}=t_{nc1nc14}^{exp}\div\frac{t_{nc1nc14}^{25}}{t_{Dmel}^{25}}\#\left( AUTONUM \backslash* Arabic \right) \end{aligned}$$

$$\begin{aligned} t_{Dmel}^{exp}=4.02e^{\frac{37.31}{T_{exp}}}\#\left( AUTONUM \backslash* Arabic \right) \end{aligned}$$

Here the symbols $t_{A}^{B}$ represent the development time. “A” represents the development duration (“nc12” represents the nuclear cycle time of nc12, “nc1nc14” represents the nuclear cycle time from nc1 to nc14, “AED” represents the AED time, “nc12start” represents the start time of nuclear cycle 12 and “Dmel” represents *Drosophila melanogaster*.). “B” represents the developmental temperature (“25” represents 25℃, “exp” represents the experimental temperature and “exp25” represents scaling the developmental time to 25℃).

**References**

1. Foe VE, Alberts BM. Studies of nuclear and cytoplasmic behaviour during the five mitotic cycles that precede gastrulation in Drosophila embryogenesis. J Cell Sci. 1983;61(1):31-70.

2. Kuntz SG, Eisen MB. Drosophila embryogenesis scales uniformly across temperature in developmentally diverse species. PLoS Genet. 2014;10(4):e1004293.
