# Supplementary material for: Attractive internuclear force drives the collective behavior of nuclear arrays in Drosophila embryos
Source: PLoS Comput Biol. 2021 Nov 19;17(11):e1009605. doi: 10.1371/journal.pcbi.1009605 (PMC8641897; doi:10.1371/journal.pcbi.1009605)
Supplement: S2 Text — (DOCX) [file pcbi.1009605.s002.docx]

**S2 Text. DNN architecture, loss function and training.**

The multi-layer feedforward neural network used here has 3 hidden layers, each with 100 nodes. The input layer has 2 nodes and the output layer has only 1 node. Softplus function ($f\left( x \right)=log\left( e^{x}+1 \right)$) is used as the activation function except for the output layer.

The loss function is defined as the Euclidean distance between the experimental data $\vec{F}_{i}^{data}$ and the training results $\vec{F}_{i}^{learned}$ of all cell nuclear array units and L2 regularization item ($\lambda\left\| w \right\|_{2}^{2}$) is added to avoid overfitting. Here $\left\| w \right\|_{2}^{2}$=$\sum_{i} |w_{i}^{2}|$ determines the complexity of the neural network and $w$ is the weight of the neural network. $\lambda$ determines the contribution of the L2 regularization item and is set as 0.01:

$$\begin{aligned} loss=\sum_{i=1}^{n} {||\vec{F}}_{i}^{learned}-\vec{F}_{i}^{data}||_{2}+\lambda\left\| w \right\|_{2}^{2}\#\left( 8 \right) \end{aligned}$$

We search the best MLFNN model ($m^{*}$) that fits the training data set (Fig 3A):

$$\begin{aligned} m^{*}=\underset{m\in\varphi:R^{2}\to R}{argmin} \left( loss \right)\#\left( 9 \right) \end{aligned}$$

The DNN is trained by Adam optimizer in tensorflow. The learning rate is set by the function tf.train.exponential_decay. The initial learning rate is 0.05 and exponentially decay in each 500 steps with the decay rate 0.96:

$$decayed\_learning\_rate=learning\_rate*decay\_rate^(global\_step/decay\_steps)$$

1D nuclear array motion dataset is used to train the DNN firstly. Note that, the data in the training dataset is sparse and discrete but the trained DNN can be used to explore a much larger parametric space. To get a continuous internuclear force field function from the trained DNN, a new input whose data range is the same with the training dataset but has a really small data interval (only 0.1) is feed to the DNN as the test set. The new input and the corresponding DNN outputs are plotted in a three dimensional space as the internuclear force field function.

The training results are not sensitive to the width of hidden layer and depth of the neural network. The DNNs have two or more hidden layers each with ten or more nodes can give out similar force fields using the same training data set.
