# Supplementary material for: Attractive internuclear force drives the collective behavior of nuclear arrays in Drosophila embryos
Source: PLoS Comput Biol. 2021 Nov 19;17(11):e1009605. doi: 10.1371/journal.pcbi.1009605 (PMC8641897; doi:10.1371/journal.pcbi.1009605)
Supplement: S3 Text — (DOCX) [file pcbi.1009605.s003.docx]

**S3 Text. The choice of the internuclear force formula**

The internuclear force can be modeled at either the molecular level or the nuclear level. At the detailed molecular level [1,2], the nuclei interact with their neighbors through a filament-motor network. The internuclear force $F_{i,j}$can be calculated by an integration as follows:

$$F_{i,j}=\iint_{D2} \left( \iint_{D1} f\left( \vec{r},\vec{r^{'}},s,s^{'} \right)d\vec{r^{'}} \right)d\vec{r} \left( \vec{r^{'}}\in D1, \vec{r}\in D2 \right), (10)$$

where *D1* is the interaction region between nucleus *i* and *j*, *D2* is the region within the nucleus *i*, and $f(\vec{r},\vec{r^{'}},s,s^{'})$ is the force acting on the molecule at the position $\vec{r}$ with the molecular state *s* by the molecule at the position $\vec{r^{'}}$ with the molecular state $s^{'}$. Here the molecular state could be the filament orientation, filament length, etc. Based on the established general framework [1,2], it is important to keep the formula of $f(\vec{r},\vec{r^{'}},s,s^{'})$ satisfying the symmetry requirements such as covariant under translations, rotations and reflections, as well as the principle of action-reaction. However, in our case the form of $f(\vec{r},\vec{r^{'}},s,s^{'})$ is very difficult to derive as the molecular environment consisting of actin filaments, microtubules, motors and membrane is very complicated and dynamic. Hence, we coarse grain the system at the nuclear level following the same approach as the previous work [3-9]. We assume that $F_{i,j}$ is the function of nuclear age (time) and nuclear density (space), i.e., $F_{i,j}=m(\bar{\tau}_{i,j},\bar{\rho}_{i,j})$, where $\bar{\tau}_{i,j} \mathrm{and}\bar{\rho}_{i,j}$describe the spatial-temporal state of the molecular environment between the two adjacent nuclei. Although coarse grained, this approach does not violate the essential physical laws or biological facts. For instance, the force formula we used still satisfies the essential symmetric requirement for the internuclear force: $\vec{F}_{i,j}=\vec{e}_{i,j}m\left( \bar{\rho}_{i,j},\bar{\tau}_{i,j} \right)=\vec{e}_{j,i}m\left( \bar{\rho}_{j,i},\bar{\tau}_{j,i} \right)=-\vec{F}_{j,i}$ (note that, $\bar{\rho}_{i,j}$=$\bar{\rho}_{j,i}$=$(\rho_{i}+\rho_{j})/2$ and $\bar{\tau}_{i,j}$=$\bar{\tau}_{j,i}$=$(\tau_{i}+\tau_{j})/2$). And we assume that all the nuclei only interact with their surrounding neighbors as previous nuclear level models [3-9], which is consistent with the molecular-level model that nearer motors have more probability to bind with cytoskeleton filaments [2]. Other different ad-hoc formulas have also been used in previous studies, for example, in the study [10] $\bar{\tau}_{i,j}=min(\tau_{i},\tau_{j})$; in the study [5], $F_{i,j}=A(\tau_{i})A(\tau_{j})\frac{1}{{r_{i,j}}^{4}}$, but the form of $A(\tau)$ is very complicated.

**References**

1. Kruse K, Jülicher F. Dynamics and mechanics of motor-filament systems. Eur Phys J E. 2006;20(4):459-65.

2. Doubrovinski K, Polyakov O, Kaschube M. A mesoscopic description of contractile cytoskeletal meshworks. Eur Phys J E. 2010;33(2):105-10.

3. Manhart A, Windner S, Baylies M, Mogilner A. Mechanical positioning of multiple nuclei in muscle cells. PLoS Comput Biol. 2018;14(6):e1006208.

4. Dutta S, Djabrayan NJ-V, Torquato S, Shvartsman SY, Krajnc M. Self-similar dynamics of nuclear packing in the early Drosophila embryo. Biophys J. 2019;117(4):743-50.

5. Lv Z, Rosenbaum J, Mohr S, Zhang X, Kong D, Preiß H, et al. The Emergent Yo-yo Movement of Nuclei Driven by Cytoskeletal Remodeling in Pseudo-synchronous Mitotic Cycles. Curr Biol. 2020, 30(13): 2564-2573. e5.

6. Kaiser F, Lv Z, Rodrigues DM, Rosenbaum J, Aspelmeier T, Großhans J, et al. Mechanical model of nuclei ordering in Drosophila embryos reveals dilution of stochastic forces. Biophys J. 2018;114(7):1730-40.

7. Tian B, Guan G, Tang L-H, Tang C. Why and how the nematode’s early embryogenesis can be precise and robust: a mechanical perspective. Phys Biol. 2020;17(2):026001.

8. Fickentscher R, Struntz P, Weiss M. Mechanical cues in the early embryogenesis of Caenorhabditis elegans. Biophys J. 2013;105(8):1805-11.

9. Yamamoto K, Kimura A. An asymmetric attraction model for the diversity and robustness of cell arrangement in nematodes. Development. 2017;144(23):4437-49.

10. Koke C, Kanesaki T, Grosshans J, Schwarz US, Dunlop CM. A computational model of nuclear self-organisation in syncytial embryos. J Theor Biol. 2014;359:92-100.
