# Supplementary material for: Attractive internuclear force drives the collective behavior of nuclear arrays in Drosophila embryos
Source: PLoS Comput Biol. 2021 Nov 19;17(11):e1009605. doi: 10.1371/journal.pcbi.1009605 (PMC8641897; doi:10.1371/journal.pcbi.1009605)
Supplement: S4 Text — (DOCX) [file pcbi.1009605.s004.docx]

**S4 Text. Ruling out the repulsive force field from the DNN learning results**

For the repulsive force field increasing with distance learned by DNN under the F^r^ assumption, it cannot maintain a stable nuclear array in the 3D simulations (S12 Fig), hence we rule out the repulsive force field.

We also discover that, the force field learned from DNNs with 1D dataset should have a general solution with an offset as Eq. (11), but it is still not a valid solution for the 3D simulations:

$$\begin{aligned} F_{i,j}^{learned}=\hat{F}_{i,j}^{learned}+\frac{C}{\rho_{i,j}}\#\left( 11 \right) \end{aligned}$$

Here $F_{i,j}^{learned}$ is a scalar and shows the magnitude of the internuclear force, and $\hat{F}_{i,j}^{learned}$ is a particular solution with an offset $C$. To calculate the resultant force in the 1D case, we can get:

$$\begin{aligned} \vec{F}_{i}^{learned}=\sum_{j\in n\left( i \right)} \hat{F}_{i,j}^{learned}\bar{\rho}_{i,j}\vec{e}_{i,j}+\sum_{j\in n\left( i \right)} C\vec{e}_{i,j}\#\left( 12 \right) \end{aligned}$$

No matter what the value of *C* is, the second term $\sum_{j\in n(i)} C\vec{e}_{i,j}$ is always equal to zero.

But when we apply the general solution in the 1D case to the 3D case, the approximate resultant force is:

$$\begin{aligned} \vec{F}_{i}^{learned}=\sum_{j\in n\left( i \right)} \hat{F}_{i,j}^{learned}\vec{e}_{i,j}+\sum_{j\in n\left( i \right)} \frac{C}{\rho_{i,j}}\vec{e}_{i,j}\#\left( 13 \right) \end{aligned}$$

Unless *C* equals to zero, in general the second term $\sum_{j\in n(i)} \frac{C}{\rho_{i,j}}\vec{e}_{i,j}$ is not equal to zero. Because of that, the general solution in the 1D case cannot be applied to the 3D case.

Under the F^r^ assumption, the particular solution $\hat{F}_{i,j}^{learned}$ learned from DNNs is approximately the opposite value of the particular solution under the F^a^ assumption (Fig 3F-H). Although we could tune the value of *C* to make the general solution $F_{i,j}^{learned}$ to be positive, it cannot be applied to the 3D case. Hence we confirm that we only obtain a particular solution under the F^r^ assumption that is equivalent to the solution under the F^a^ assumption.

Moreover, we could not find a molecular mechanism supporting the force field learned from DNN under the F^r^ assumption. On the one hand, the repulsive force from microtubules crosslinking with motor protein kinesin-5 is unlikely to increase with distance. The magnitude of the repulsive force scales with microtubule overlap length and motor number [1]. But, there is no evidence showing that in the *Drosophila* embryo internuclear distance scales with microtubule overlap length and motor number. And if the number of kinesin-5 is limited, the repulsive force provided by microtubules should decrease along with the internuclear distance or be independent of internuclear distance [2]. On the other hand, the depolymerization of spindle microtubules cannot account for the dramatic decrease of the repulsive force after the onset of anaphase. The nuclear motion pattern is significantly reduced after injecting the Rok inhibitor Y-27623 to inhibit the activation of myosin II [3]. But the inhibitor Y-27623 does not influence the function of microtubules, so that the spindle microtubule depolymerization process should be maintained. This result indicates that the spindle microtubule depolymerization process cannot be the driving force for the nuclear motion pattern and is not the reason for the decrease of repulsive internuclear force after the onset of anaphase.

**References**

1. Shimamoto Y, Forth S, Kapoor TM. Measuring pushing and braking forces generated by ensembles of kinesin-5 crosslinking two microtubules. Dev Cell. 2015;34(6):669-81.

2. Manhart A, Windner S, Baylies M, Mogilner A. Mechanical positioning of multiple nuclei in muscle cells. PLoS Comput Biol. 2018;14(6):e1006208.

3. Lv Z, Rosenbaum J, Mohr S, Zhang X, Kong D, Preiß H, et al. The Emergent Yo-yo Movement of Nuclei Driven by Cytoskeletal Remodeling in Pseudo-synchronous Mitotic Cycles. Curr Biol. 2020, 30(13): 2564-2573. e5.
