# Supplementary material for: Attractive internuclear force drives the collective behavior of nuclear arrays in Drosophila embryos
Source: PLoS Comput Biol. 2021 Nov 19;17(11):e1009605. doi: 10.1371/journal.pcbi.1009605 (PMC8641897; doi:10.1371/journal.pcbi.1009605)
Supplement: S5 Text — (DOCX) [file pcbi.1009605.s005.docx]

**S5 Text. Evaluate the ground truth recovery by the DNN**

To evaluate whether DNN can recover the ground truth force field, we performed the control DNN training by feeding the simulation data generated by the 3D surface simulations and comparing the learned force fields with the ground truth force fields. The 3D simulation data are used to create the 1D dataset as shown in the Materials and methods. For both attractive force field (Fig 4) and repulsive force field (S20 Fig), the learned force fields are consistent with the ground truth: *F* shows consistent dependence with *r* and *T* (S13 Fig). However, the magnitude of *F* deviates from ground truth. This discrepancy may result from the difference of the 3D data and the 1D dataset. But we confirm that the 3D simulation is insensitive on the force magnitude, it recapitulates the characteristic features of the collective motion and packing patterns of the nuclear array as long as the characteristic features (the correlation between *F*, *r* and *T* as shown in S16A-B Fig) of the force field are maintained. Hence our DNN methods can extract the main features of the real internuclear force field.
