# Supplementary material for: Attractive internuclear force drives the collective behavior of nuclear arrays in Drosophila embryos
Source: PLoS Comput Biol. 2021 Nov 19;17(11):e1009605. doi: 10.1371/journal.pcbi.1009605 (PMC8641897; doi:10.1371/journal.pcbi.1009605)
Supplement: S6 Text — (DOCX) [file pcbi.1009605.s006.docx]

**S6 Text. Deterministic mean-field physical model**

In this deterministic mean-field physical model, we assume the nuclear motion is a passive indicator of its surrounding fluidic medium. The general momentum equation of fluid can be described with Eq. (14).

$$\begin{aligned} \rho\frac{d\vec{v}}{dt}=\vec{f}-\nabla p+\nabla\cdot\vec{T}\#\left( \text{14} \right) \end{aligned}$$

, where $\rho$ is density, $\vec{v}$ is the velocity field, $\vec{f}$ is the mean body force, $p$ is the effective pressure representing the effect of nuclear interaction from the mean-field perspective, and $\vec{T}$ is the shear stress.

For simplicity, considering a psudo-1D (as the nuclear movement is dominant along the AP axis), overdamped cortical environment omitting the shear stress, we have the following momentum equation describing the connection between the effective pressure $p\left( x,t \right)$and velocity field$v\left( x,t \right) (as shown in S15A Fig)$,

$$\begin{aligned} \tilde{\gamma}v\left( x,t \right)=-\frac{\partial}{\partial x}p\left( x,t \right)\#\left( 15 \right) \end{aligned}$$

, where the mean body force is contributed only by viscosity, $\tilde{\gamma}$ is the effective friction coefficient. This equation is consistent with the particle-based moment equation of the nucleus in the main text. Moreover, we assume that the effective pressure $p\left( x,t \right)$ is determined by the physical state, i.e., surface density $\rho\left( x,t \right)$ (S15B Fig) and nuclear age $\tau\left( x,t \right)$ (S15C Fig). We calculated this dependency (i.e., the equation of state $p=p(\rho,\tau)$) as follows.

First, we integrated Eq. (15) at a reference time point $t_{0}$ yielding

$$\begin{aligned} p\left( x,t_{0} \right)=I\left( x,t_{0} \right)+C_{0}\#\left( 16 \right) \end{aligned}$$

, where $I\left( x,t_{0} \right)=-\tilde{\gamma}\int_{x_{0}}^{x} v\left( x,t_{0} \right)dx$ is the special integration of the velocity field (S15A Fig) at time $t_{0}$, $C_{0}$ is the constant of integration.

Since the effective pressure is determined by the physical state, we obtain the following relation from Eq (16).

$$\begin{aligned} p\left( \rho\left( x,t_{0} \right),\tau\left( x,t_{0} \right) \right)=p\left( x,t_{0} \right)=I\left( x,t_{0} \right)+C_{0}\#\left( 17 \right) \end{aligned}$$

, which means the equation of state $p=p(\rho,\tau)$ is obtained in a restricted range of the variable space (the ρ-τ plane). This variable range can be described as

$$\begin{aligned} \left( \rho,\tau\right)\in R_{0}=\left\{ \left( \rho,\tau\right) | \exists x, s.t. \rho=\rho\left( x,t_{0} \right), \tau=\tau\left( x,t_{0} \right) \right\}\#\left( 18 \right) \end{aligned}$$

Then we integrate Eq.(14) at the next time point $t_{1}$ yielding

$$\begin{aligned} p\left( \rho\left( x,t_{1} \right),\tau\left( x,t_{1} \right) \right)=p\left( x,t_{1} \right)=I\left( x,t_{1} \right)+C_{1}\#\left( 19 \right) \end{aligned}$$

, which contains another constant of integration $C_{1}$, and yielding the equation of state $p=p\left( \rho,\tau\right)$ at another variable range

$$\begin{aligned} \left( \rho,\tau\right)\in R_{1}=\left\{ \left( \rho,\tau\right) | \exists x, s.t. \rho=\rho\left( x,t_{1} \right), \tau=\tau\left( x,t_{1} \right) \right\}\#\left( 20 \right) \end{aligned}$$

The intersection points of these two range ($R_{0}\bigcap R_{1}$) share the same physical state, hence, the effective pressure given by Eq. (17) and Eq. (19) at these points should be identical, based on which the uncertainty of $C_{1}$ is eliminated (S15A Fig).

By combining the integration results of Eq. (15) at time point $t_{0}$ and $t_{1}$, the equation of state $p=p(\rho,\tau)$ is derived at a variable range $R_{0}\bigcup R_{1}$. Repeating this calculation at different time points results in a widened variable range on which the equation of state emerges (S15B Fig).

Surprisingly, this derived equation of state $p=p(\rho,\tau)$ shows a strong dependency on the nuclear age $\tau$ as a “negative-pulse-shape” curve (S15D Fig), and no dependency on the surface density $\rho$ (S15B Fig). Notably, as an attractive force, the negative-pulse actually represents the increase of the force magnitude, consistent with the result from the DNN training (Fig 3E).

This age dependency can be formulated as

$$\begin{aligned} p\left( \rho,\tau\right)=p\left( \tau\right)=p_{0}+p_{m}\left\{ \frac{\tanh\left[ k\left( \tau-\tau_{0} \right) \right]-B}{1+B} \right\}^{2}\#\left( 21 \right) \end{aligned}$$

, where $p_{0}$ is the offset of the curve resulted from the integration constant $C_{0}$, $p_{m}$ is the scale factor resulted from the uncertainty of $\tilde{\gamma}$ in Eq. (20), $k$, $\tau_{0}$ and $B$ together depict the shape of the curve (S15C Fig).
