# Supplementary material for: Attractive internuclear force drives the collective behavior of nuclear arrays in Drosophila embryos
Source: PLoS Comput Biol. 2021 Nov 19;17(11):e1009605. doi: 10.1371/journal.pcbi.1009605 (PMC8641897; doi:10.1371/journal.pcbi.1009605)
Supplement: S7 Text — (DOCX) [file pcbi.1009605.s007.docx]

**S7 Text. Equations of motion in polar coordinates on the prolate spheroid surface.**

We use the prolate-spheroidal coordinate system $\left( \nu,\theta,\varphi\right)$, which can be transformed into Cartesian coordinates $\left( x,y,z \right)$ by:

$$\begin{aligned} x=a\sinh\nu\sin\theta\cos\varphi\#\left( 22 \right) \end{aligned}$$

$$\begin{aligned} y=a\sinh\nu\sin\theta\sin\varphi\#\left( 23 \right) \end{aligned}$$

$$\begin{aligned} z=a\cosh\nu\cos\theta\#\left( 24 \right) \end{aligned}$$

Now let

$$\begin{aligned} R_{1}=a\sinh\nu\#\left( 25 \right) \end{aligned}$$

$$\begin{aligned} R_{2}=a\cosh\nu\#\left( 26 \right) \end{aligned}$$

, which are semi-minor and the semi-major axes of the spheroid.

In Cartesian coordinates $\left( x,y,z \right)$, consider line element $d\vec{s}=d\vec{x}+d\vec{y}+d\vec{z}$, we have:

$$\begin{aligned} ({d\vec{s})}^{2}={(d\vec{x})}^{2}+{(d\vec{y})}^{2}+{(d\vec{z})}^{2}\#\left( 27 \right) \end{aligned}$$

In prolate-spheroidal coordinates $\left( \nu,\theta,\varphi\right)$, assume that ${(d\vec{s})}^{2}$ has the form:

$$\begin{aligned} {(d\vec{s})}^{2}={A(d\vec{\nu})}^{2}+{B(d\vec{\theta})}^{2}+{C(d\vec{\varphi})}^{2}\#\left( 28 \right) \end{aligned}$$

Consider $x(\nu,\theta,\varphi)$,

$$\begin{aligned} d\vec{x}=\frac{\partial x}{\partial\nu}d\vec{\nu}+\frac{\partial x}{\partial\theta}d\vec{\theta}+\frac{\partial x}{\partial\varphi}d\vec{\varphi}\#\left( 29 \right) \end{aligned}$$

similar with $d\vec{y}, d\vec{z}$.

Substituting $d\vec{x},d\vec{y}, and d\vec{z}$into Eq. (27), ${(d\vec{s})}^{2}$ can be expressed as:

$$\begin{aligned} {(d\vec{s})}^{2}=\left( \frac{\partial x}{\partial\nu}d\vec{\nu}+\frac{\partial x}{\partial\theta}d\vec{\theta}+\frac{\partial x}{\partial\psi}d\vec{\varphi} \right)^{2}+\left( \frac{\partial y}{\partial\nu}d\vec{\nu}+\frac{\partial y}{\partial\theta}d\vec{\theta}+\frac{\partial y}{\partial\varphi}d\vec{\varphi} \right)^{2}+ \\ \left( \frac{\partial z}{\partial\nu}d\vec{\nu}+\frac{\partial z}{\partial\theta}d\vec{\theta}+\frac{\partial z}{\partial\varphi}d\vec{\varphi} \right)^{2}\#\left( 30 \right) \end{aligned}$$

Because $\left( \nu,\theta,\varphi\right)$is an orthogonal coordinate system, the cross term is removed, therefore we have:

$${(d\vec{s})}^{2}=\left[ \left( \frac{\partial x}{\partial\nu} \right)^{2}+\left( \frac{\partial y}{\partial\nu} \right)^{2}+\left( \frac{\partial z}{\partial\nu} \right)^{2} \right]{(d\vec{\nu})}^{2}+\left[ \left( \frac{\partial x}{\partial\theta} \right)^{2}+\left( \frac{\partial y}{\partial\theta} \right)^{2}+\left( \frac{\partial z}{\partial\theta} \right)^{2} \right]{(d\vec{\theta})}^{2}+[\left( \frac{\partial x}{\partial\varphi} \right)^{2}+\left( \frac{\partial y}{\partial\varphi} \right)^{2}+\left( \frac{\partial z}{\partial\varphi} \right)^{2}]{(d\vec{\varphi})}^{2} (31)$$

Thus,

$$\begin{aligned} A=\left( \frac{\partial x}{\partial\nu} \right)^{2}+\left( \frac{\partial y}{\partial\nu} \right)^{2}+\left( \frac{\partial z}{\partial\nu} \right)^{2}=R_{1}^{2}\left( 1+b\sin^{2}\theta\right)\#\left( 32 \right) \end{aligned}$$

$$\begin{aligned} B=\left( \frac{\partial x}{\partial\theta} \right)^{2}+\left( \frac{\partial y}{\partial\theta} \right)^{2}+\left( \frac{\partial z}{\partial\theta} \right)^{2}=R_{1}^{2}\left( 1+b\sin^{2}\theta\right)\#\left( 33 \right) \end{aligned}$$

$$\begin{aligned} C=\left( \frac{\partial x}{\partial\varphi} \right)^{2}+\left( \frac{\partial y}{\partial\varphi} \right)^{2}+\left( \frac{\partial z}{\partial\varphi} \right)^{2}=R_{1}^{2}\sin^{2}\theta\#\left( 34 \right) \end{aligned}$$

, where $b={(\frac{R_{2}}{R_{1}})}^{2}-1$.

So

$$\begin{aligned} \gamma\frac{d\vec{s}}{dt}=\gamma\left[ R_{1}\sqrt{1+b\sin^{2}\theta}\frac{d\vec{\nu}}{dt}+R_{1}\sqrt{1+b\sin^{2}\theta}\frac{d\vec{\theta}}{dt}+R_{1}\sin\theta\frac{d\vec{\varphi}}{dt} \right]\#\left( 35 \right) \end{aligned}$$

Now consider force *F* is the gradient of a potential *U*, then in the coordinates $\left( \nu,\theta,\varphi\right)$

$$\begin{aligned} \vec{F}=-\nabla U=\left( -\frac{\partial U}{\partial s_{\nu}},-\frac{\partial U}{\partial s_{\theta}},-\frac{\partial U}{\partial s_{\varphi}} \right)=\left( -\frac{\partial U}{\sqrt{A}\partial\nu},-\frac{\partial U}{\sqrt{B}\partial\theta},-\frac{\partial U}{\sqrt{C}\partial\varphi} \right)\#\left( 36 \right) \end{aligned}$$

Finally, we have

$$\begin{aligned} \vec{F}=-\left[ \frac{1}{R_{1}\sqrt{1+b\sin^{2}\theta}}\frac{\partial U}{\partial\vec{\nu}}+\frac{1}{R_{1}\sqrt{1+b\sin^{2}\theta}}\frac{\partial U}{\partial\vec{\theta}}+\frac{1}{R_{1}\sin\theta}\frac{\partial U}{\partial\vec{\varphi}} \right]\#\left( 37 \right) \end{aligned}$$

On the other hand, by overdamped equation of motion

$$\begin{aligned} \vec{F}= \gamma\frac{d\vec{s}}{dt}\#\left( 38 \right) \end{aligned}$$

Compare Eq. (35) with Eq. (37), we get

$$\begin{aligned} \mu R_{1}\sqrt{1+b\sin^{2}\theta}\frac{dv}{dt}=\frac{-1}{R_{1}\sqrt{1+b\sin^{2}\theta}}\frac{\partial U}{\partial v}\#\left( 39 \right) \end{aligned}$$

$$\begin{aligned} {\mu R}_{1}\sqrt{1+b\sin^{2}\theta}\frac{d\theta}{dt}=\frac{-1}{R_{1}\sqrt{1+b\sin^{2}\theta}}\frac{\partial U}{\partial\theta}\#\left( 40 \right) \end{aligned}$$

$$\begin{aligned} {\mu R}_{1}\sin\theta\frac{d\varphi}{dt}=\frac{-1}{R_{1}\sin\theta}\frac{\partial U}{\partial\varphi}\#\left( 41 \right) \end{aligned}$$

As $\nu$ is a constant, from Eq. (40) and Eq. (41), we have

$$\begin{aligned} \frac{d\theta}{dt}=\frac{-1}{{\mu R}_{1}^{2}\left( 1+b\sin^{2}\theta\right)}\frac{\partial U}{\partial\theta}\#\left( 42 \right) \end{aligned}$$

$$\begin{aligned} \frac{d\varphi}{dt}=\frac{-1}{{\mu R}_{1}^{2}\sin^{2}\theta}\frac{\partial U}{\partial\varphi}\#\left( 43 \right) \end{aligned}$$

where

$$\begin{aligned} \frac{\partial U}{\partial\theta}=\frac{\partial U\left( r,T \right)}{\partial r}\frac{\partial r}{\partial\theta}\#\left( 44 \right) \end{aligned}$$

$$\begin{aligned} \frac{\partial U}{\partial\varphi}=\frac{\partial U\left( r,T \right)}{\partial r}\frac{\partial r}{\partial\varphi}\#\left( 45 \right) \end{aligned}$$

, which is consistent with ref [1].The Partial derivative $\frac{\partial U}{\partial r}$ can be defined by internuclear force $F(r,T)$, which is the function of internuclear distance *r* and nuclear age *T*. The curvature difference on the ellipsoid surface may produce errors for internuclear Euclidean distance. Instead, we calculate the geodesic on the ellipsoid as the internuclear distance. The distance calculation of *r* (geodesic on the ellipsoid) and its derivatives with respect to $\theta$ and $\varphi$ are quoted from the methods part from ref [1].

**References**

1. Dutta S, Djabrayan NJ-V, Torquato S, Shvartsman SY, Krajnc M. Self-similar dynamics of nuclear packing in the early Drosophila embryo. Biophys J. 2019;117(4):743-50.
