# Supplementary material for: Attractive internuclear force drives the collective behavior of nuclear arrays in Drosophila embryos
Source: PLoS Comput Biol. 2021 Nov 19;17(11):e1009605. doi: 10.1371/journal.pcbi.1009605 (PMC8641897; doi:10.1371/journal.pcbi.1009605)
Supplement: S8 Text — (DOCX) [file pcbi.1009605.s008.docx]

**S8 Text. The force fields and the initial nuclear age used in the simulations.**

Consider about the potential nonlinear relationship between *F* and *r*, the distance dependent force *B(r*) under F^a^ assumption is amended to:

$$\begin{aligned} B\left( \bar{r}_{i,j} \right)=\frac{F_{0}}{2r_{0}^{2}}\bar{r}_{i,j}^{2}-\frac{F_{0}}{2}\#\left( 46 \right) \end{aligned}$$

Here, the parameters used are listed in S1 Table.

Under the F^r^ force field base on previous models is defined as:

$$\begin{aligned} F_{i,j}\left( \tau_{i,}\tau_{j,}\vec{r}_{i,}\vec{r}_{j} \right)=\left\{ \begin{aligned} A\left( \bar{\tau}_{i,j} \right)B\left( \bar{r}_{i,j} \right) \bar{r}_{i,j}\geq r_{0} \\ B\left( \bar{r}_{i,j} \right) \bar{r}_{i,j}<r_{0} \end{aligned} \right.\#\left( 47 \right) \end{aligned}$$

$$\begin{aligned} A\left( \bar{\tau}_{i,j} \right)=\left\{ \begin{aligned} F_{2}+\left( \cos(\frac{\bar{\tau}_{i,j}}{t_{1}}\pi)+1 \right)\left( \frac{F_{1}-F_{2}}{2} \right) \left( 0\leq\bar{\tau}_{i,j}<2t_{1} \right) \\ F_{1} \left( \bar{\tau}_{i,j}\geq2t_{1} \right) \end{aligned} \right.\#\left( 48 \right) \end{aligned}$$

$$\begin{aligned} B\left( \bar{r}_{i,j} \right)=\left\{ \begin{aligned} F_{0}\left( 1-\frac{\bar{r}_{i,j}}{r_{1}} \right) \left( \bar{r}_{i,j}<r_{1} \right) \\ 0 \left( \bar{r}_{i,j}\geq r_{1} \right) \end{aligned} \right.\#\left( 49 \right) \end{aligned}$$

The $F^{r}$ force field is shown in S16B Fig, and the parameters used are listed in S1 Table.

The initial nuclear age of the nuclei along the AP axis is defined as:

$$\begin{aligned} T_{ini}=a\cdot\frac{\left| z \right|}{R_{2}}+b\#\left( 50 \right) \end{aligned}$$

The initial age is shown in S16C Fig, and the parameters used are listed in S1 Table.

To adjust the spatial origin of the mitotic wave, the initial nuclear age is amended to:

(1) Mitotic waves start at different time from the anterior and posterior pole:

$$\begin{aligned} T_{ini}=a\cdot\frac{\left| z-z_{dif} \right|}{R_{2}}+b\#\left( 51 \right) \end{aligned}$$

The parameters used are listed in S1 Table.

(2) Two-node standing wave of the AP speed:

$$\begin{aligned} T_{ini}=a\cdot\frac{z+R_{2}}{R_{2}}+b\#\left( 52 \right) \end{aligned}$$

The parameters used are listed in S1 Table.

(3) Five-node standing wave of the AP speed:

$$\begin{aligned} T_{ini}=a\cdot\left| \frac{\left| z \right|}{R_{2}}-c \right|+b\#\left( 53 \right) \end{aligned}$$

The parameters used are listed in S1 Table.
