# Supplementary material for: Attractive internuclear force drives the collective behavior of nuclear arrays in Drosophila embryos
Source: PLoS Comput Biol. 2021 Nov 19;17(11):e1009605. doi: 10.1371/journal.pcbi.1009605 (PMC8641897; doi:10.1371/journal.pcbi.1009605)
Supplement: S1 Table — (DOCX) [file pcbi.1009605.s009.docx]

**S1 Table. Parameters used in the simulations.**

| **Parameter** | **Comment** | **Value** | **Source** |
| --- | --- | --- | --- |
| $t_{1}$ | half period time of the AP speed standing wave | 0.23 | measurement  (Fig 2D, nuclear cycle time is set as 1) |
| $F_{1}$ | the maximum magnitude of the age dependent force function *A(T)* | 1 | fitting  (Fig 4E and 4F) |
| $F_{2}$ | the minimum magnitude of the age dependent force function *A(T)* | 0.2 | fitting  (Fig 4E and 4F) |
| $F_{0}$ | the maximum magnitude of the repulsive force of the distance dependent force function *B(r)* | 15 (Eq. (9) in the main text)  250 (Eq. (47) in the SI) | fitting  (Fig 4E and 4F) |
| $r_{0}$ | the range of the core region of the distance dependent force function *B(r)* | 8.5 μm | fitting  (Fig 4E and 4F) |
| $r_{1}$ | the valid range of the distance dependent force function *B(r)* | 18.5 μm | fitting  (Fig 4E and 4F) |
| $R_{1}$ | semi-minor axis length of the prolate spheroid | 75 μm | measurement (the average embryo size, Fig 1) |
| $R_{2}$ | semi-major axis length of the prolate spheroid | 250 μm | measurement (the average embryo size, Fig 1) |
| $z$ | nuclear position along the AP axis | [-250,250] | fixed based on $R_{2}$ |
| $c$ | the start position control parameter of mitotic waves | 0.5 | fixed |
| $z_{dif}$ | the control parameter of the start time difference of the mitotic wave from the two poles | -3, -1, 1, 3  (Fig 5C) | fixed |
| $n_{1}$ | initial nuclear number | 400 | measurement (the nuclear number in NC10) |
| $n_{2}$ | final nuclear number after nuclear division | 800 | measurement (the nuclear number in NC11) |
| $\Delta\theta$ or $\Delta\varphi$ | angle variation range of the two daughter nuclei relative to the mother nucleus after metaphase | [-5×10^-4^,5×10^-4^] | fixed |
| $\delta\theta$ or $\delta\varphi$ | angle update value per simulation step | 2.5×10^-3^ | fixed |
| $\delta t$ | nuclear age update value per simulation step | 4×10^-4^ | fixed |
