# Supplementary figures and images for: Attractive internuclear force drives the collective behavior of nuclear arrays in Drosophila embryos
Source: PLoS Comput Biol. 2021 Nov 19;17(11):e1009605. doi: 10.1371/journal.pcbi.1009605 (PMC8641897; doi:10.1371/journal.pcbi.1009605)

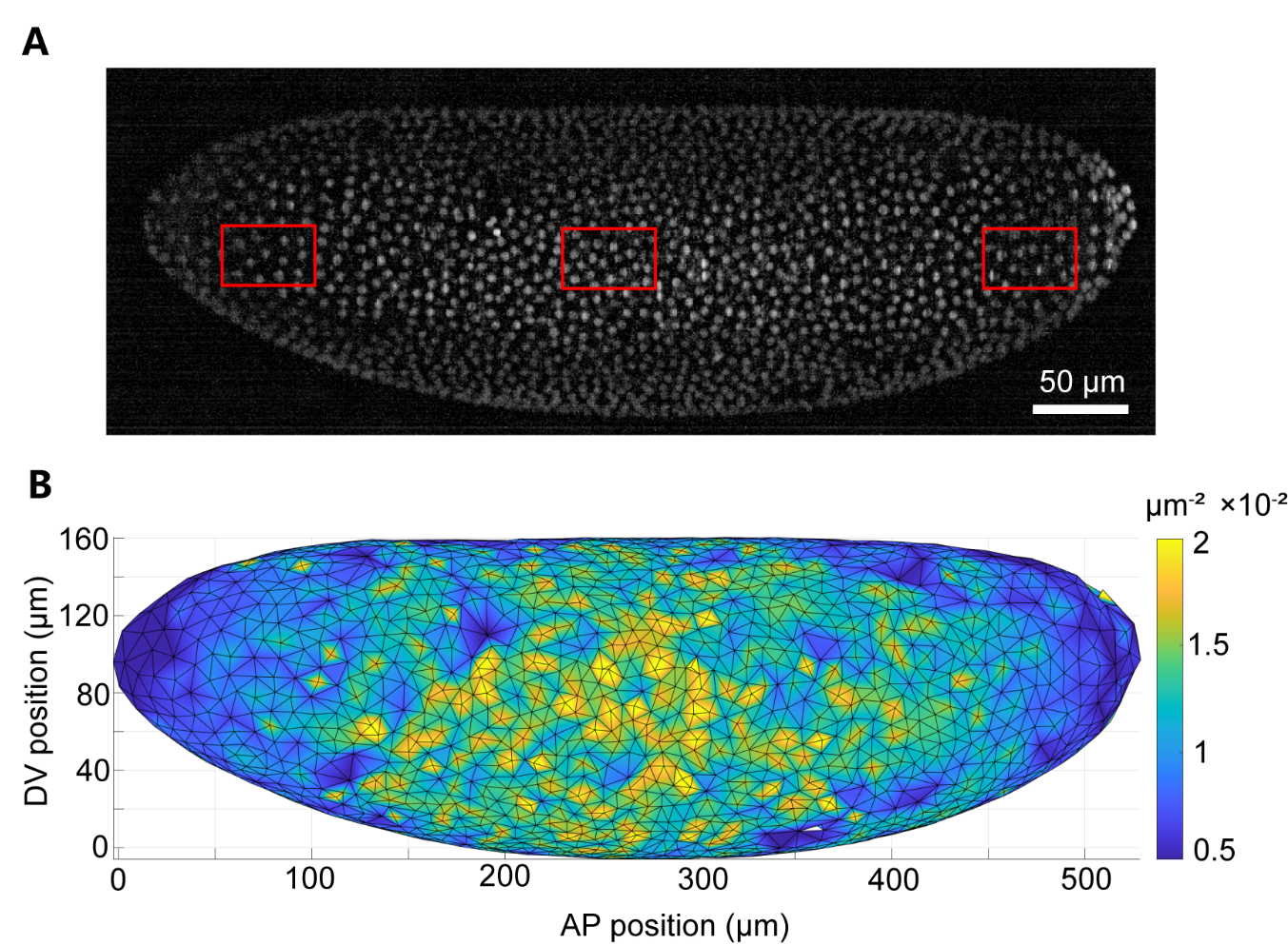

Supplement: S1 Fig — (A) The maximum intensity projection of a z stack of the light sheet images. The embryo is expressing H2Av-GFP and the imaging time is a time point at interphase 13 when the nuclear array is stable. The three equal sized red squares mark three regions in the anterior, middle and posterior of the embryo. (B) Trisurf plot of the point cloud data of the nuclear position in A. The color of each triangle vertex illustrates the density of each nucleus. Note that, the nuclear density is calculated by the reciprocal of Voronoi area of each nucleus. (TIF) [file pcbi.1009605.s013.tif]

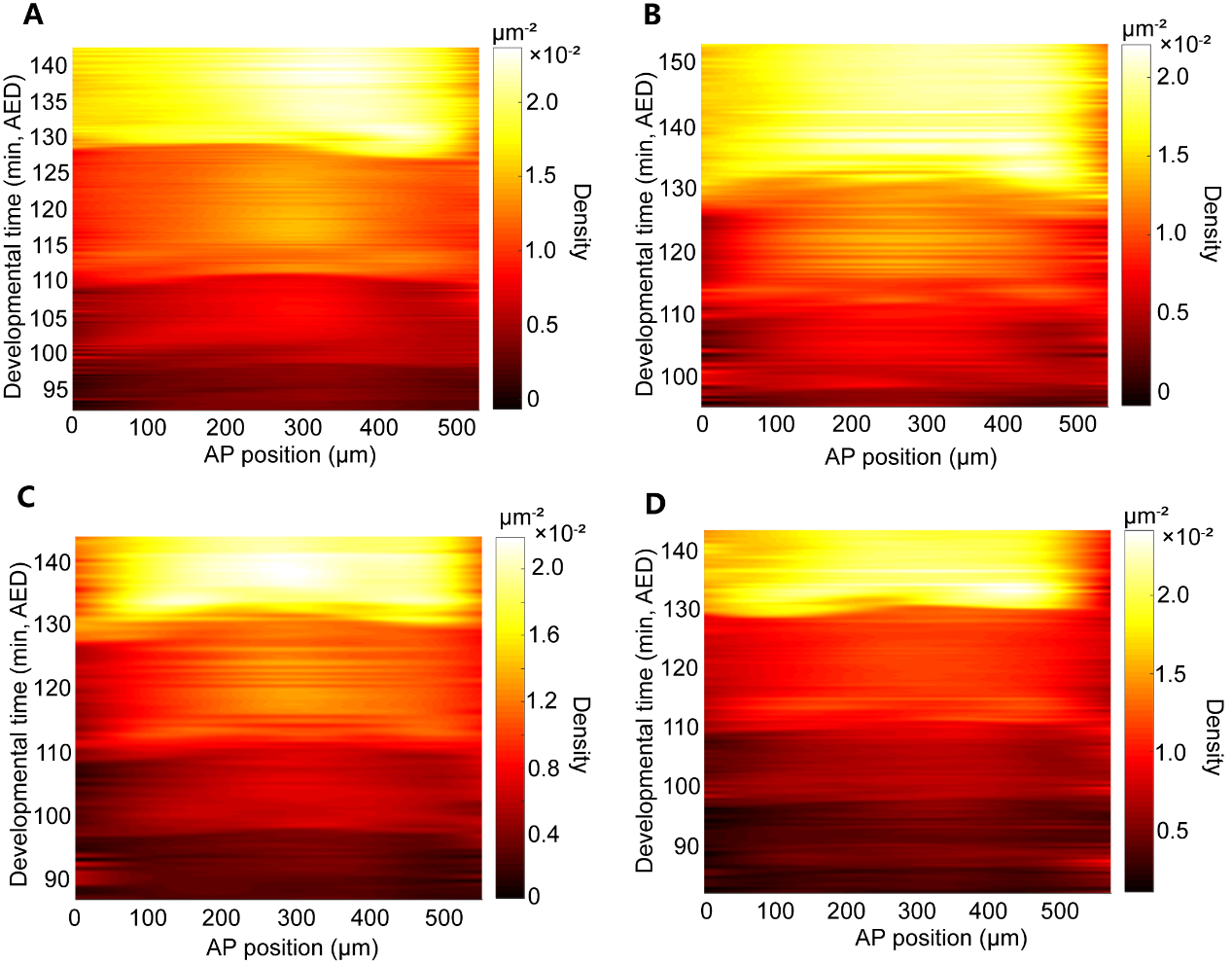

Supplement: S2 Fig — Heat maps of the nuclear density projected along the AP axis in an embryo imaged at 19.1°C (A), 20.6°C (B), 24.4°C (C), and 21.3°C (D)). The AED time was rescaled at 25°C (see S1 Text). (TIF) [file pcbi.1009605.s014.tif]

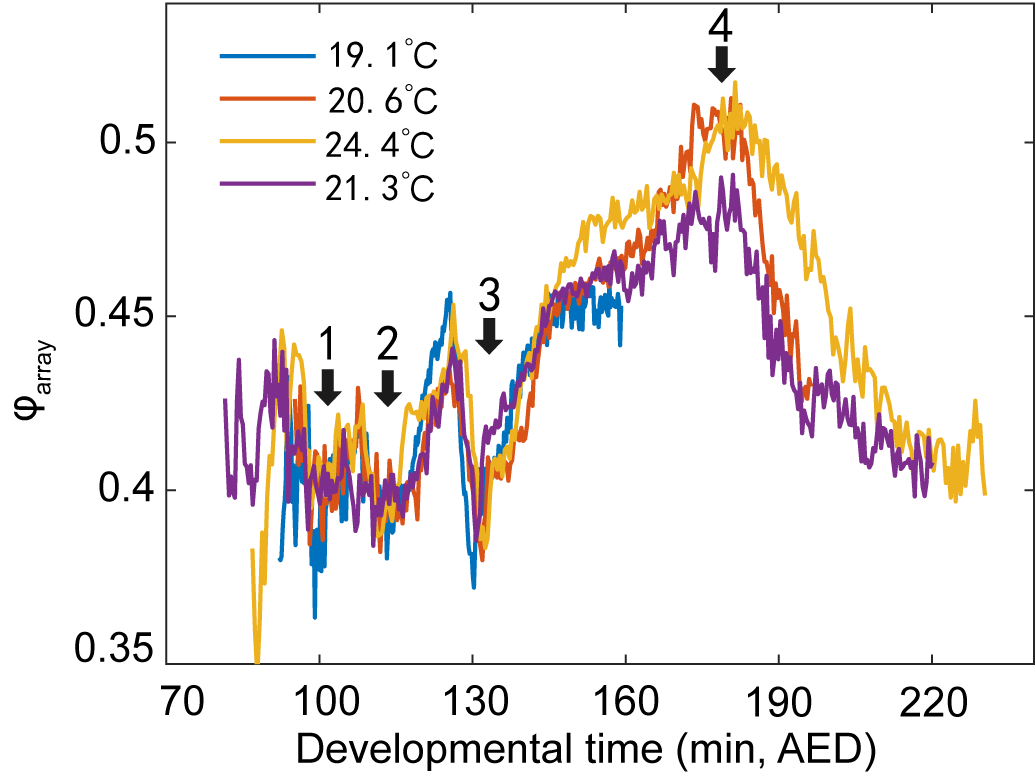

Supplement: S3 Fig — The black arrows 1, 2 and 3 label the time points of the minimum order around M phase 11, 12, and 13, respectively. The arrow 4 labels the starting time point of gastrulation, after which the nuclear array order drops dramatically. The imaging temperature of the four embryos is estimated to be 19.1°C, 20.6°C, 24.4°C, and 21.3°C respectively, and the AED time was rescaled at 25°C (see S1 Text). (TIF) [file pcbi.1009605.s015.tif]

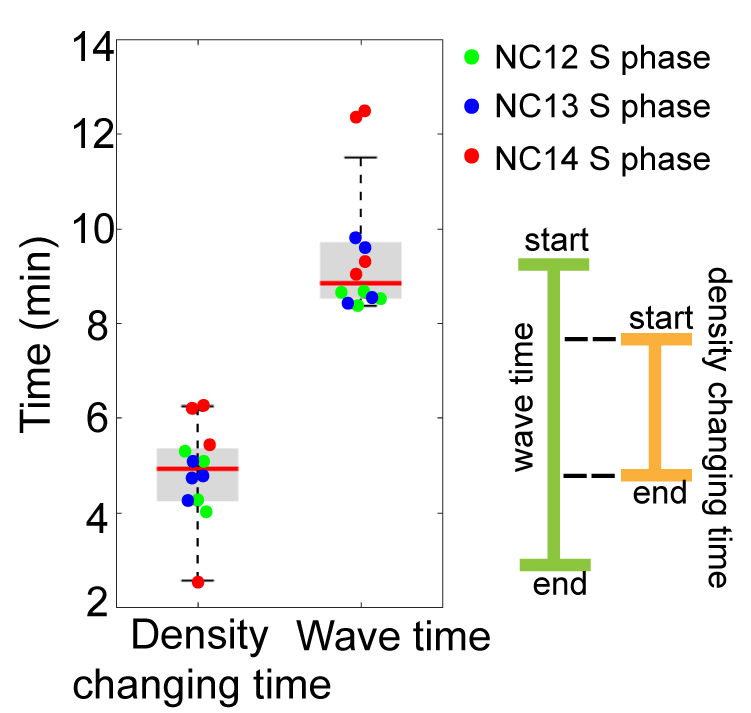

Supplement: S4 Fig — Boxplots (whisker, min/max values, boxes, 25/75 percentiles). The median of the density changing time and wave time is 4.9 min and 8.8 min, respectively, in four embryos. The density changing time is during the middle period of the wave time, in which the nuclear speed is relatively high. (TIF) [file pcbi.1009605.s016.tif]

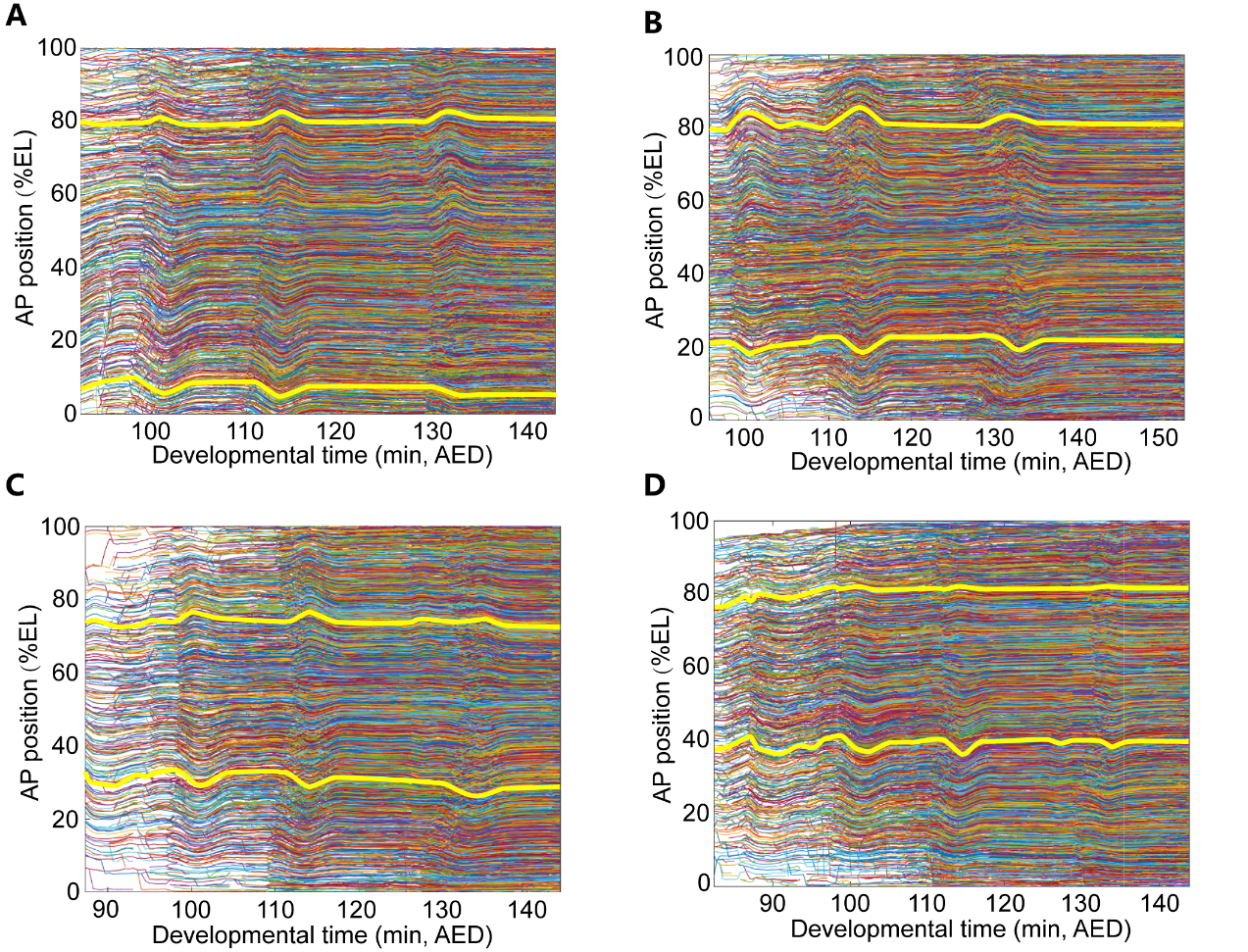

Supplement: S5 Fig — The nuclear trajectories were projected to the AP axis of the embryo. Yellow lines highlight the typical trajectories among all nuclear trajectories in each embryo. The imaging temperature of the four embryos was estimated to be 19.1°C, 20.6°C, 24.4°C, and 21.3°C, respectively. The AED time was rescaled at 25°C (see S1 Text). (TIF) [file pcbi.1009605.s017.tif]

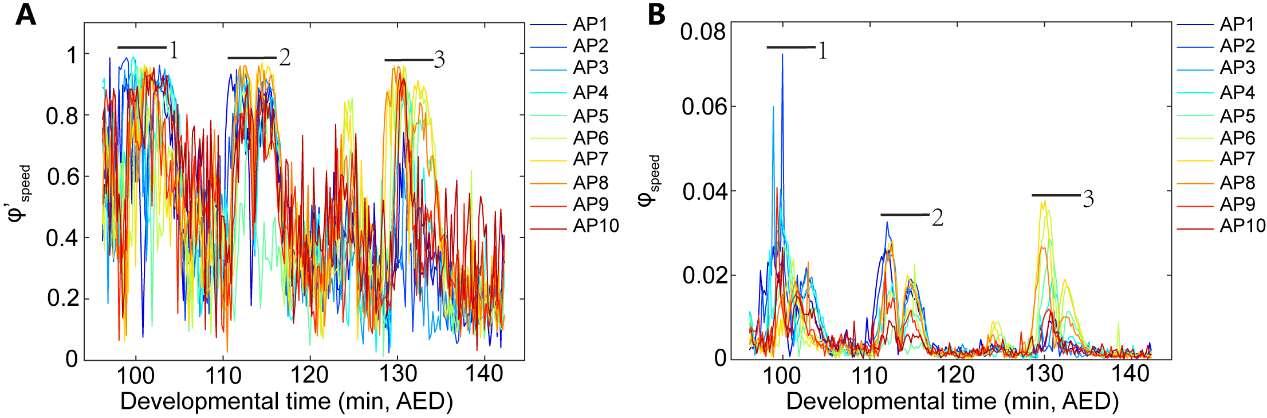

Supplement: S6 Fig — The order parameter of all the nuclei in each bin (for more details, see Materials and methods). APi corresponds to the ith bin with the width of 10% EL from the anterior pole. The markers 1, 2 and 3 label three time intervals that show high motion collectivity in (A) and (B). The AED time was rescaled at 25°C (see S1 Text). (TIF) [file pcbi.1009605.s018.tif]

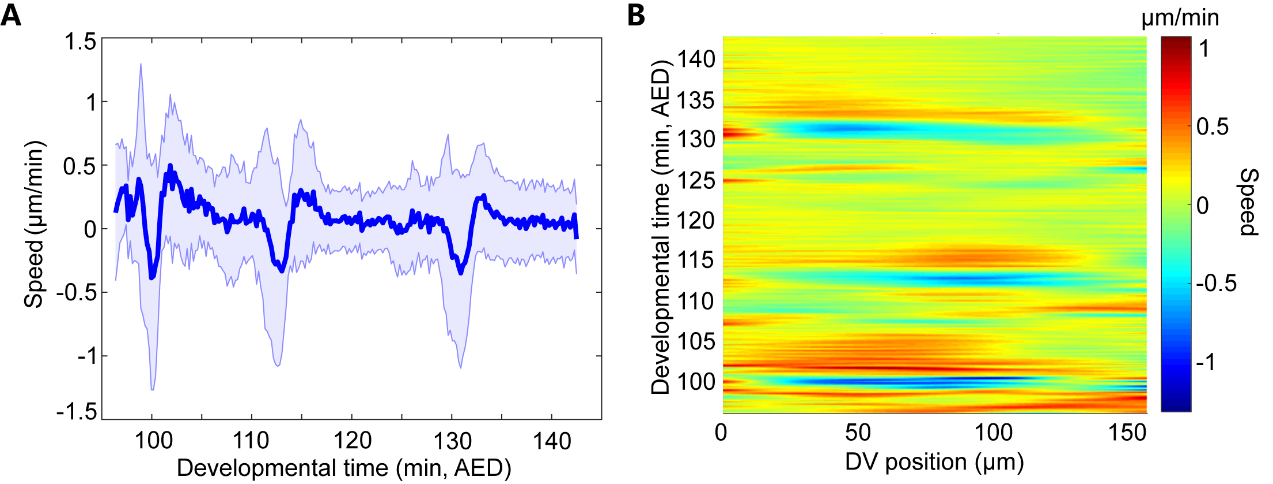

Supplement: S7 Fig — (A) The mean (line) and SD (shadow) of the nuclear speed projected along the DV axis as a function of the developmental time AED. (B) Heat map of the nuclear speed projected along the DV axis. The AED time was rescaled at 25°C (see S1 Text). (TIF) [file pcbi.1009605.s019.tif]

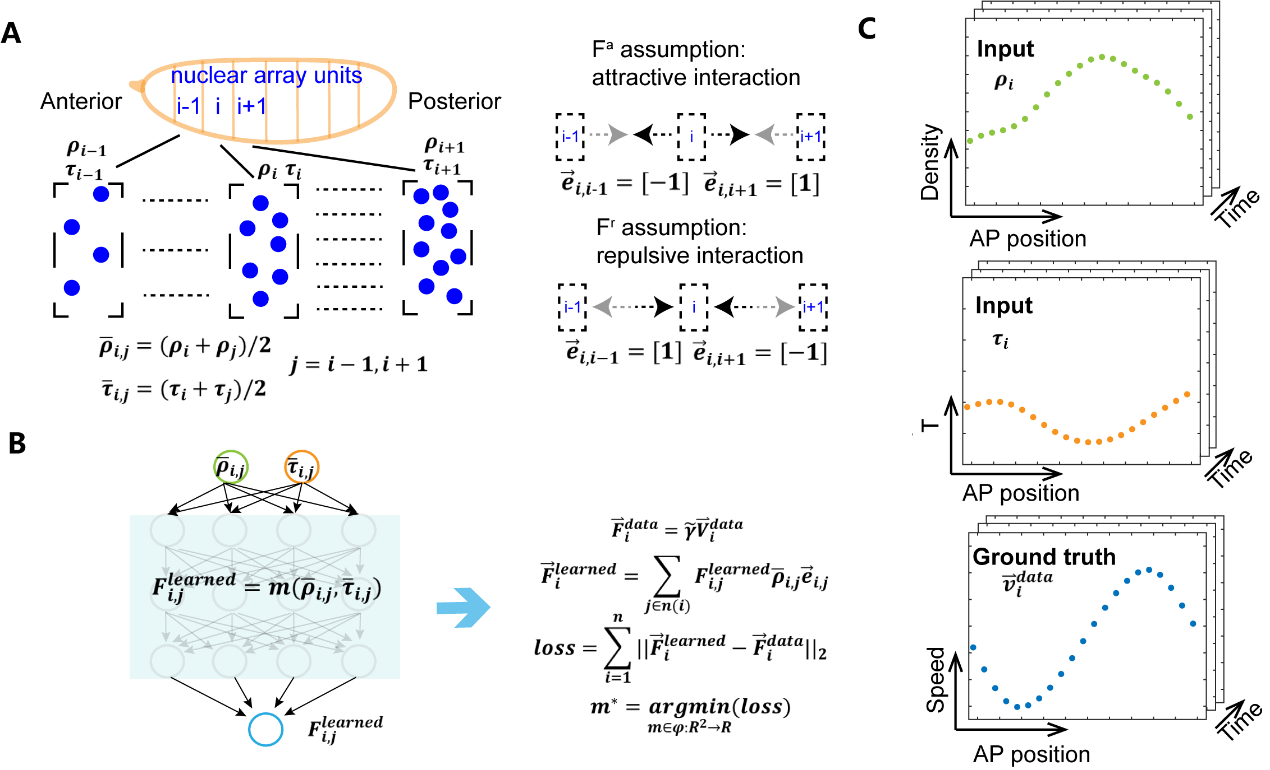

Supplement: S8 Fig — (A) The ith nuclear array unit only interacts with its nearest neighbors (the jth units, where j = i-1 and i+1). Based on Fa (or Fr) assumption, the orientation of the pairwise internuclear force e→i,j (black arrow) are different. The average nuclear density (ρ¯i,j) and average nuclear age (τ¯i,j) are the mean of the values of the adjacent nuclear array units. The pairwise internuclear interaction number (dashed line) is positively correlated with ρ¯i,j. (B) The pairwise internuclear force (Fi,jlearned) is the function of ρ¯i,j and τ¯i,j. And Fi,jlearned(ρ¯i,j,τ¯i,j) can be approximated by a three-layer MLFNN. The resultant force of a nuclear array unit F→ilearned is calculated by adding the pairwise internuclear force Fi,jlearnede→i,j from the adjacent units of the ith nuclear array unit (F→ilearned=∑j∈n(i)Fi,jlearnedρ¯i,je→i,j). Then the learned resultant force (F→ilearned) is compared to the ground truth (F⃑idata=γ˜V⃑idata) to define the loss function (loss=∑i=1n||F→ilearned−F→idata||2) for training. Here only the first item of the loss function is shown. For more details of the training, see S2 Text. (C) Snapshots of the training dataset. All the data are discretized along the AP axis (see Materials and methods). (TIF) [file pcbi.1009605.s020.tif]

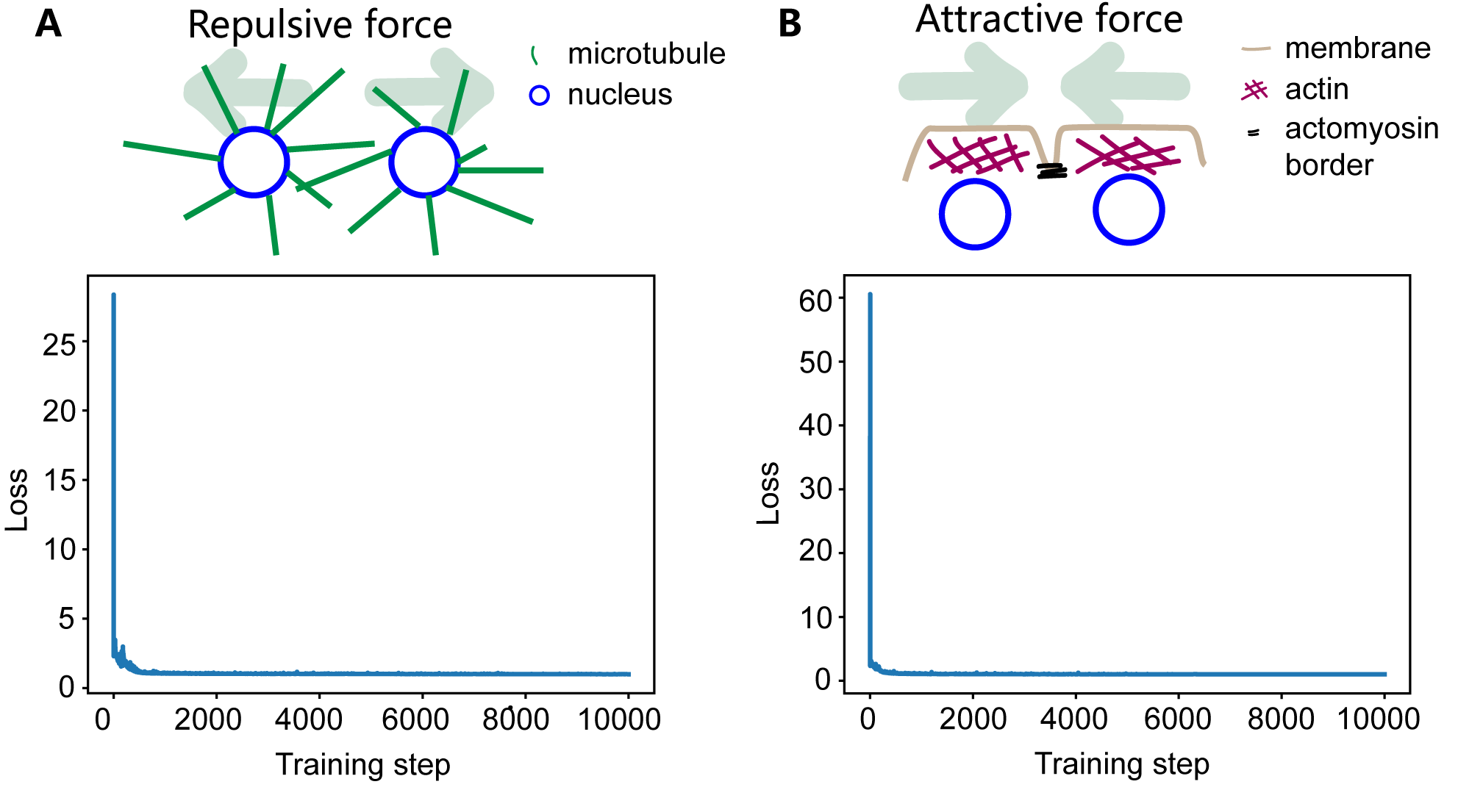

Supplement: S9 Fig — The loss functions while training the DNN. The data from M phase 13 to interphase 14 in one embryo (3078 data points in total) is used while training. The training is based on two interaction assumptions: net effective repulsive force (Fr) (A) and net effective attractive force (Fa) (B). (TIF) [file pcbi.1009605.s021.tif]

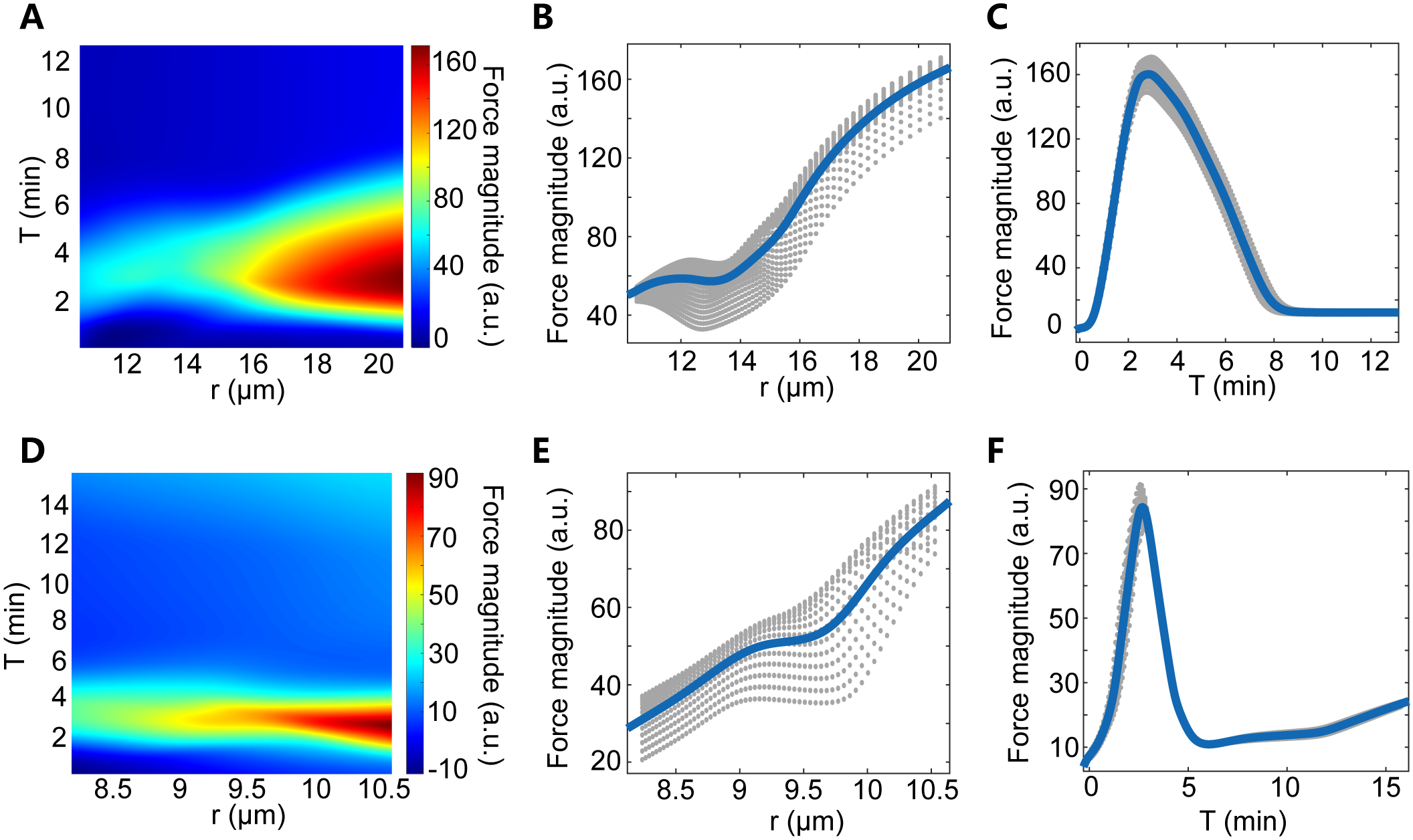

Supplement: S10 Fig — The DNN learning results are based on the Fa assumption. (A-C) The data from M phase 11 to interphase 12 in one embryo (2394 data points in total) is used while training the DNN. (A) A representative heat map of the function F(T,r). Here F is the magnitude of the internuclear force (Fi,jlearned), T is the nuclear age after the onset of anaphase and r is the internuclear distance. Note that, r=s and ρ=1s, here s is the Voronoi area of the nuclei. (B) F has a positive correlation with r as T = 1.9–3.8 min. (C) F has a conservative pulsatile relationship with T as r = 18.3–20.8 μm. (D-F) The DNN learning results as in A-C. The data from M phase 12 to interphase 13 in one embryo (3002 data points in total) is used while training the DNN. (E) T = 1.9–3.2 min. (F) r = 10–10.5 μm. (TIF) [file pcbi.1009605.s022.tif]

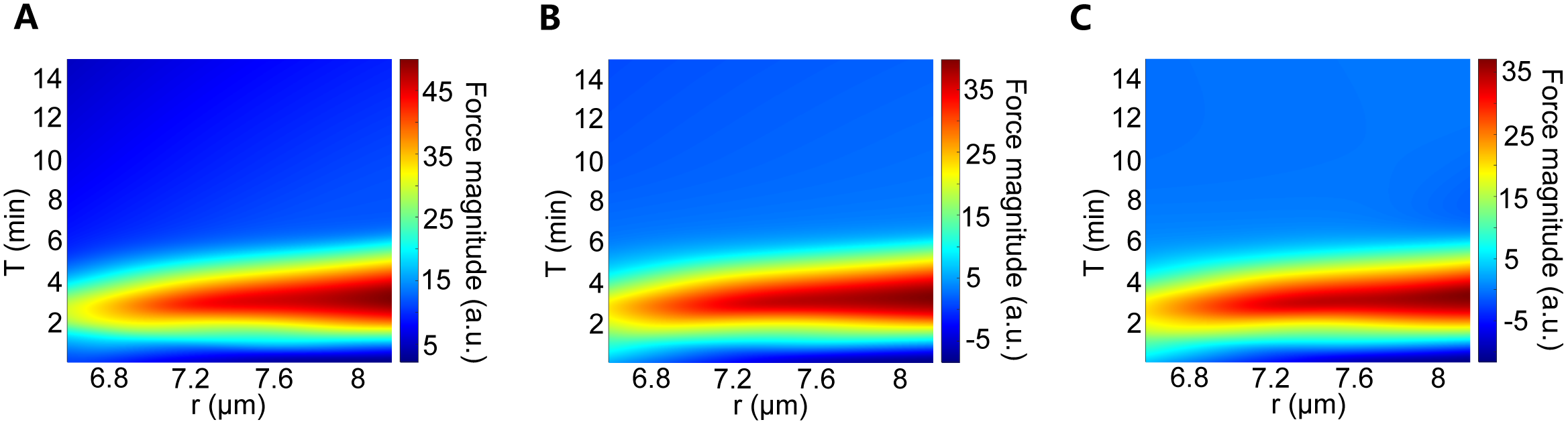

Supplement: S11 Fig — The data from M phase 13 to interphase 14 in one embryo (3078 data points in total) is used while training the DNN. Three randomly selected DNN learning results are shown in A-C. (TIF) [file pcbi.1009605.s023.tif]

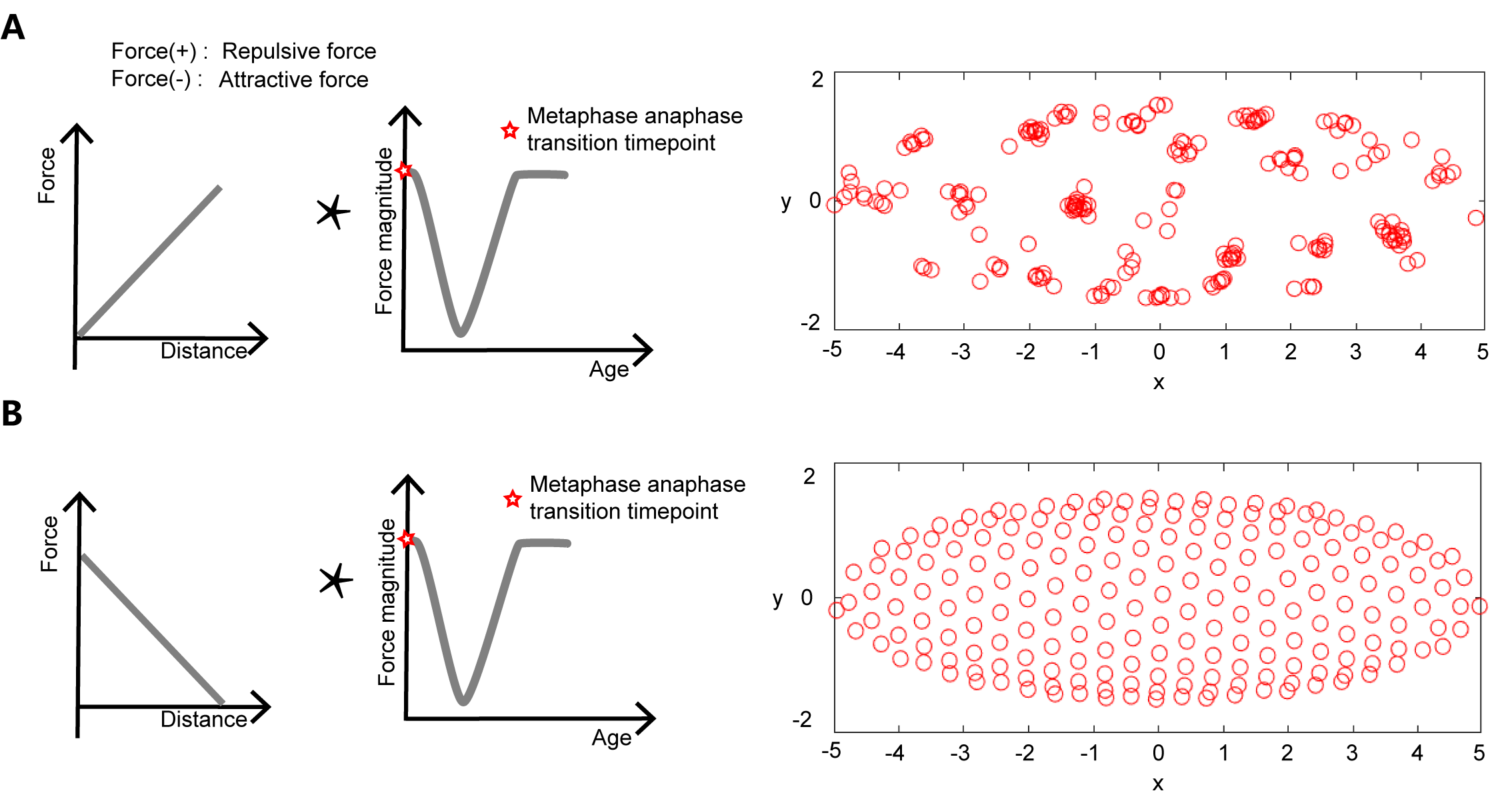

Supplement: S12 Fig — (A) If the magnitude of the repulsive internuclear force (F) linearly increases with the internuclear distance (r), no stable nuclear array can be generated during interphase. The nuclei form aggregations on the ellipsoid surface. (B) If the magnitude of the repulsive force F linearly decreases with r, a stable nuclear array can be generated during interphase. For simulation details, see Materials and methods. (TIF) [file pcbi.1009605.s024.tif]

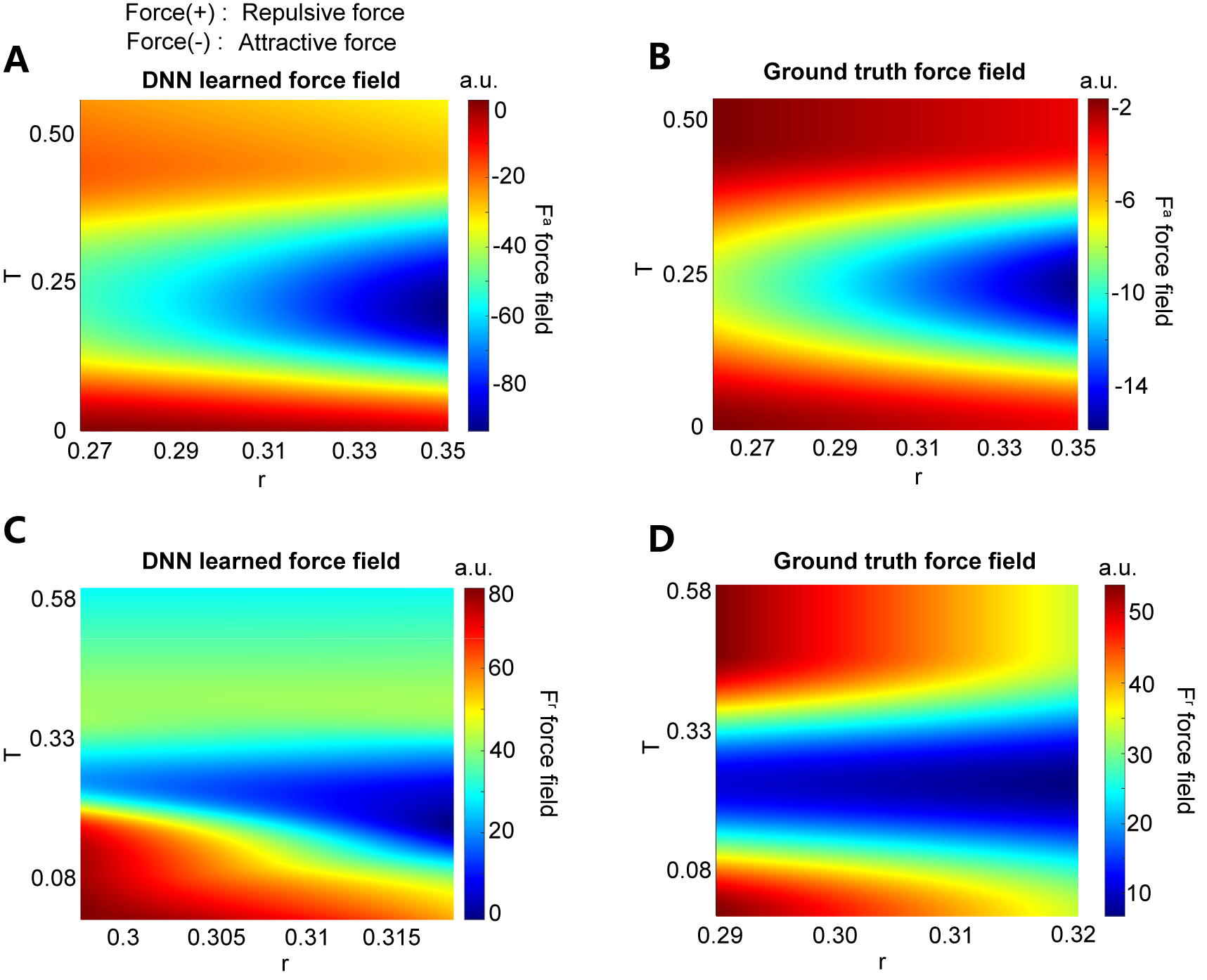

Supplement: S13 Fig — (A-B) Comparison between the learned force field (A) and the ground truth force field used in the simulation in Fig 4 (B) for the attractive force. (C-D) Comparison between the learned force field (C) and the ground truth force field used in the simulation in S20 Fig (D) for the repulsive force. (TIF) [file pcbi.1009605.s025.tif]

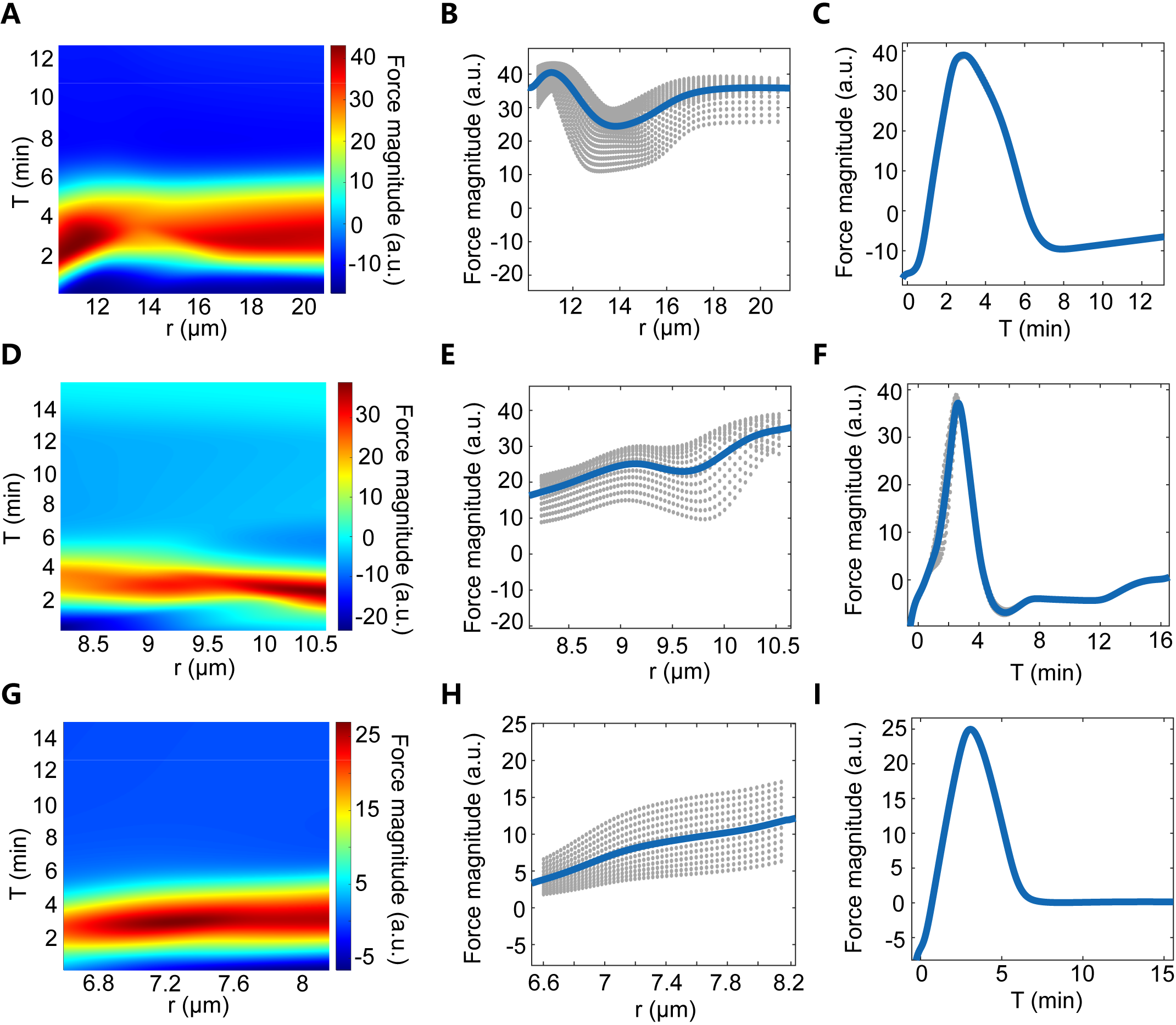

Supplement: S14 Fig — The DNN learning results are based on the Fa assumption. But the resultant force F→ilearned is calculated by adding the resultant force Fi,jlearnede→i,j from the neighboring units of the ith nuclear array unit (F→ilearned=∑j∈n(i)Fi,jlearnede→i,j). (A-C) The data from M phase 11 to interphase 12 in one embryo (2394 data points in total) is used while training the DNN. (A) A representative heat map of the function F(T,r). (B) F is independent on r as T = 1.9–3.8 min. (C) F has a conservative pulsatile relationship with T as r = 18.3–20.8 μm. (D-F) The DNN learning results as in A-C. The data from M phase 12 to interphase 13 in one embryo (3002 data points in total) is used while training the DNN. (E) T = 1.9–3.2 min. (F) r = 10–10.5 μm. (G-I) The data from M phase 13 to interphase 14 in one embryo (3078 data points in total) is used while training the DNN. (H) T = 4.4–5.6 min. (I) r = 7.9–8.2 μm. (TIF) [file pcbi.1009605.s026.tif]

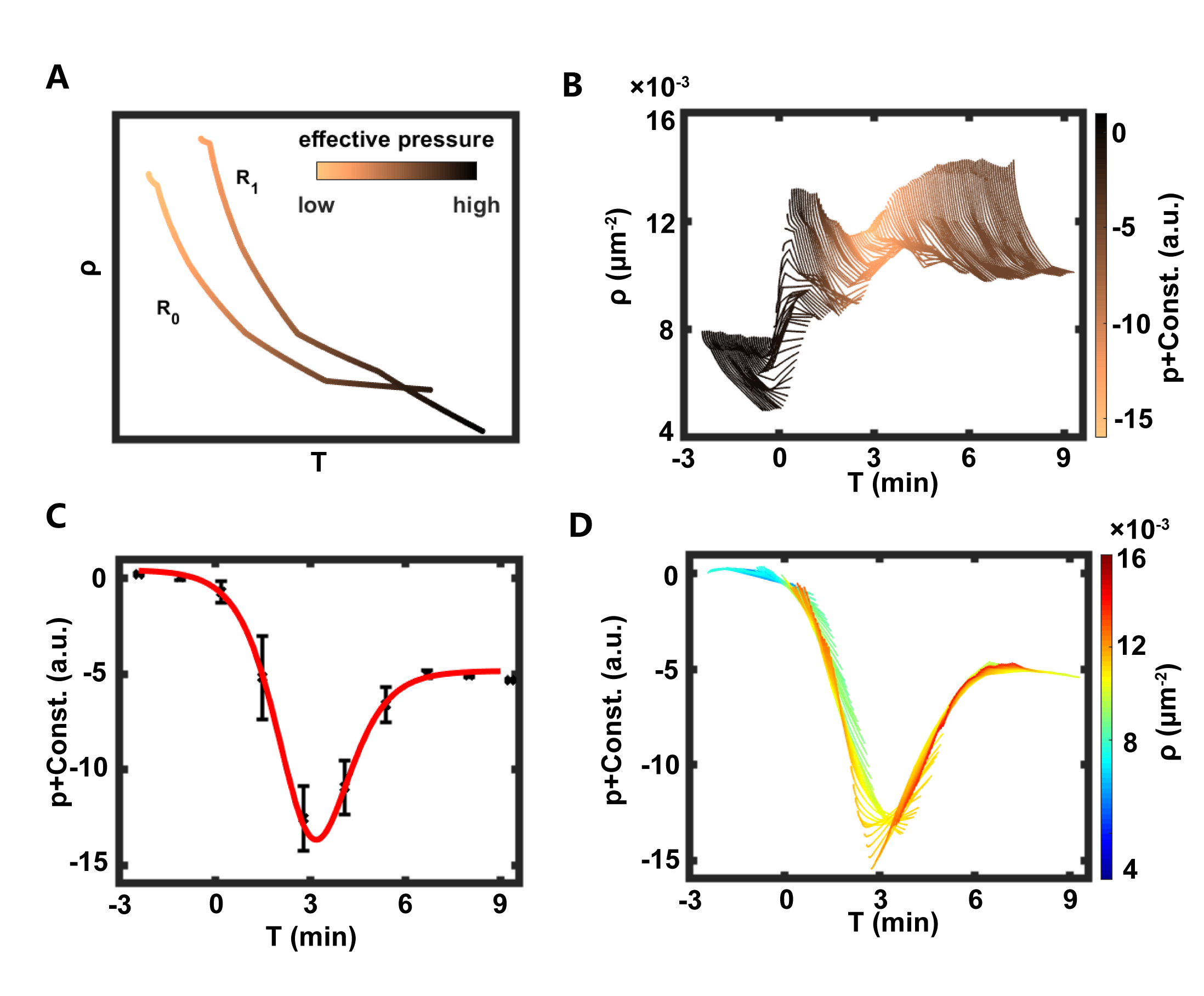

Supplement: S15 Fig — (A) Integration of the momentum equation γv(x,t)=−∂∂xp(x,t) in two different time points results in two range in the ρ−τ plane, R0 and R1. (B) By integrating γv(x,t)=−∂∂xp(x,t) at different time points, the equation of state emerged from the resulted range in the ρ−τ plane. (C) Formulating the “negative pulse shape” p−τ curve. Error bar is the binned standard deviation of the result. (D) The effective pressure is mainly determined by the age while the effect of density is subtle. (TIF) [file pcbi.1009605.s027.tif]

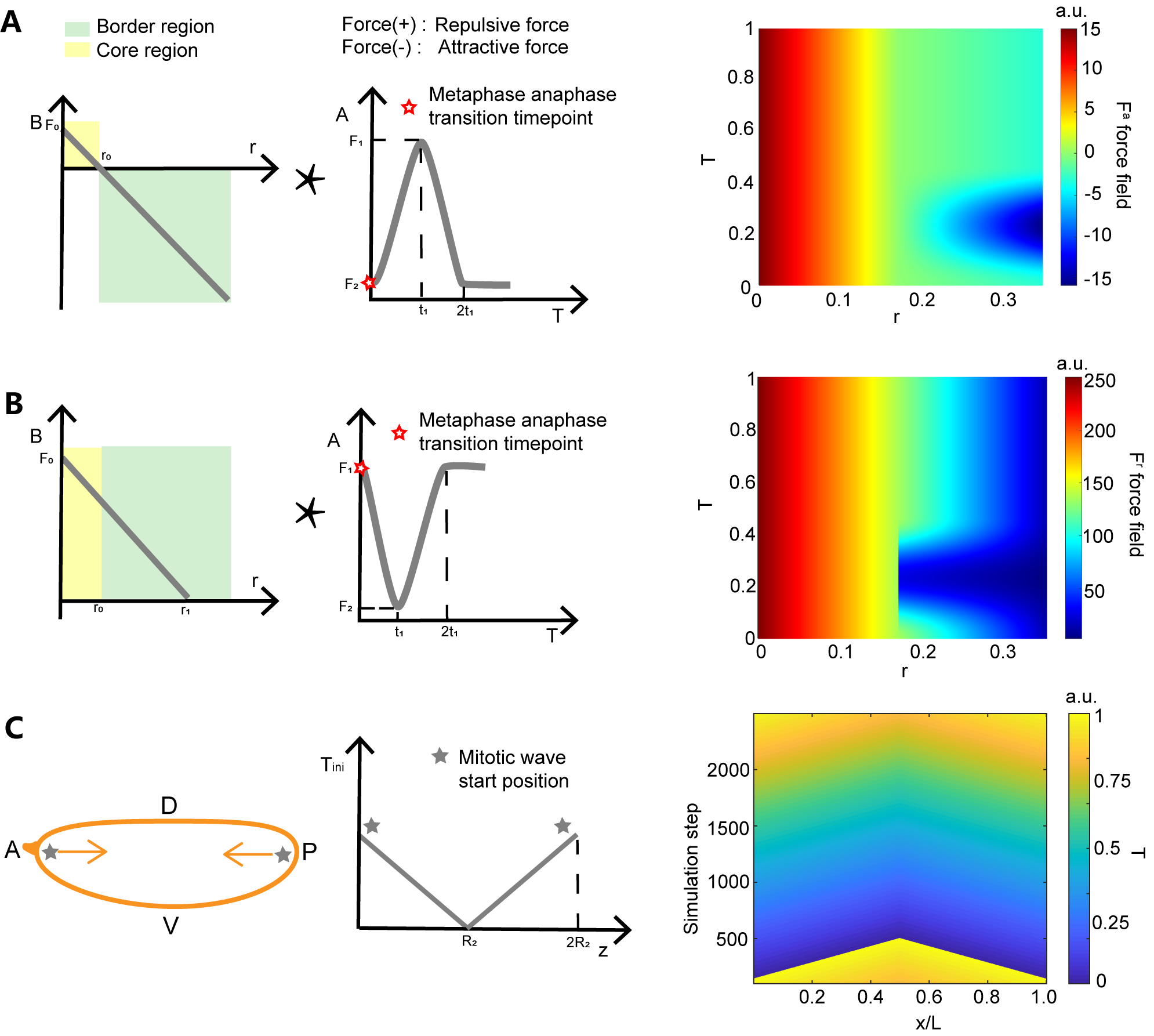

Supplement: S16 Fig — (A, B) The green squares show the border regions in which the distance dependent force function multiplies with the time dependent force function. The yellow squares show the core regions of the distance dependent force. The attractive force field and repulsive force field are shown in the heat maps. Note that, attractive direction is the positive direction and repulsive direction is the negative direction. (C) Mitotic waves start from the anterior pole and posterior pole of the embryo (gray stars indicate the mitotic wave start point), so that the nuclear age has phase difference along the AP axis. Heat map shows the average nuclear age along the AP axis in each simulation step. (TIF) [file pcbi.1009605.s028.tif]

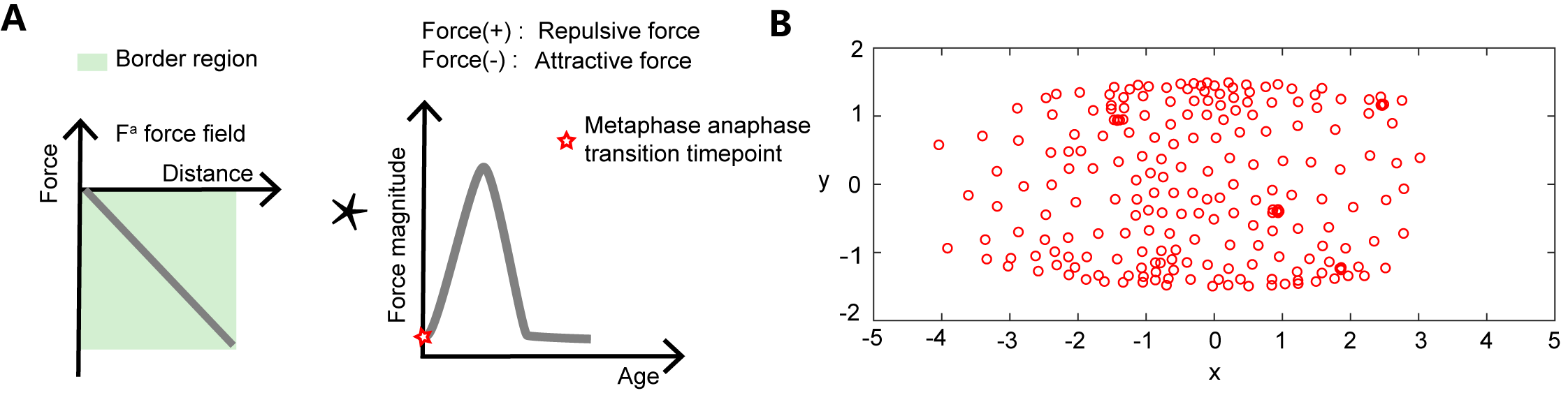

Supplement: S17 Fig — (A) The force field used in the simulation. (B) The nuclear array distribution during the interphase with a very high nuclear density in the middle and a very low nuclear density in the poles. (TIF) [file pcbi.1009605.s029.tif]

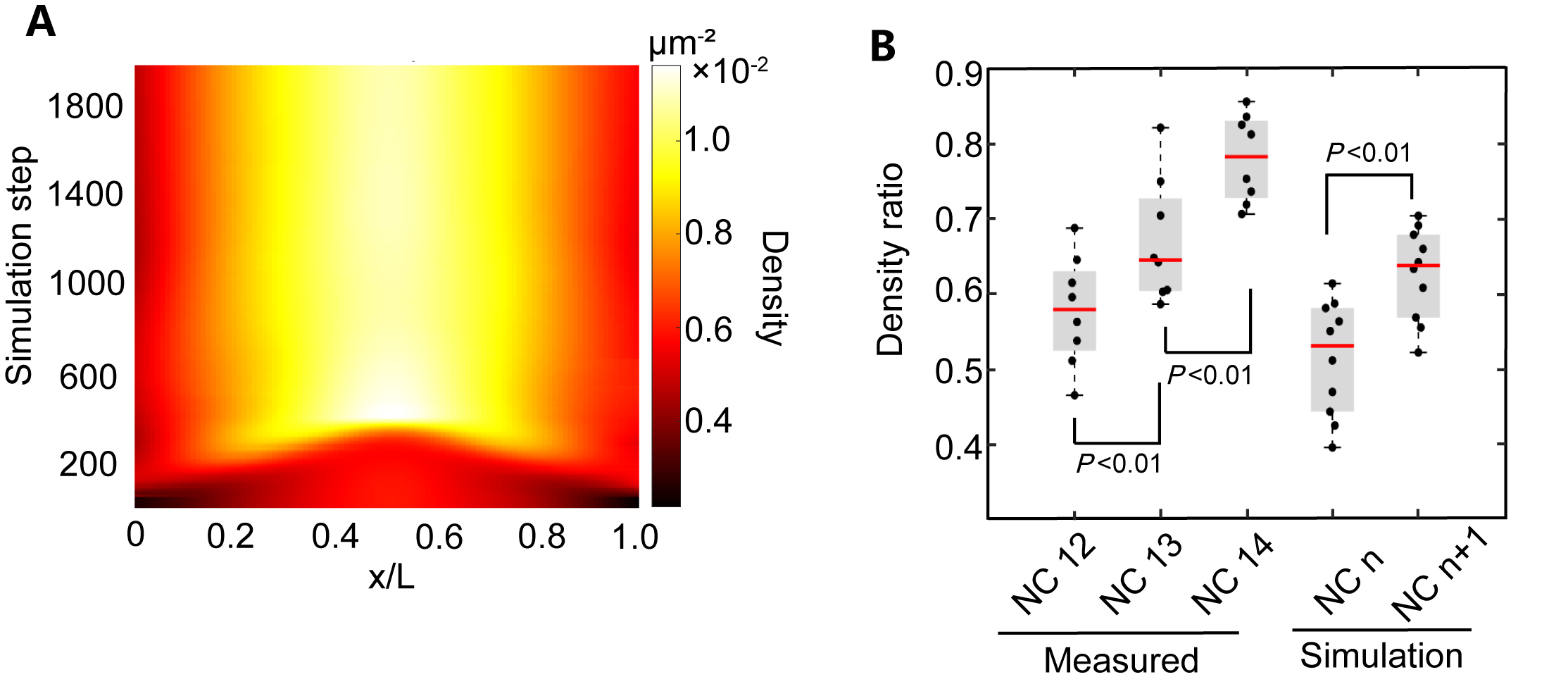

Supplement: S18 Fig — (A) Heat map of the nuclear density projected along the AP axis. (E) Boxplots (whisker, min/max values, boxes, 25/75 percentiles). The medians (red line) of measured and simulated density ratio are 0.58, 0.65, 0.78, 0.53 and 0.64, respectively. Density ratio is defined as the ratio between the anterior (~5–15% EL) or posterior (~85–95% EL) density and the maximal density in the middle of the embryo during interphase. The force field used in this simulation is shown in S16A Fig. (TIF) [file pcbi.1009605.s030.tif]

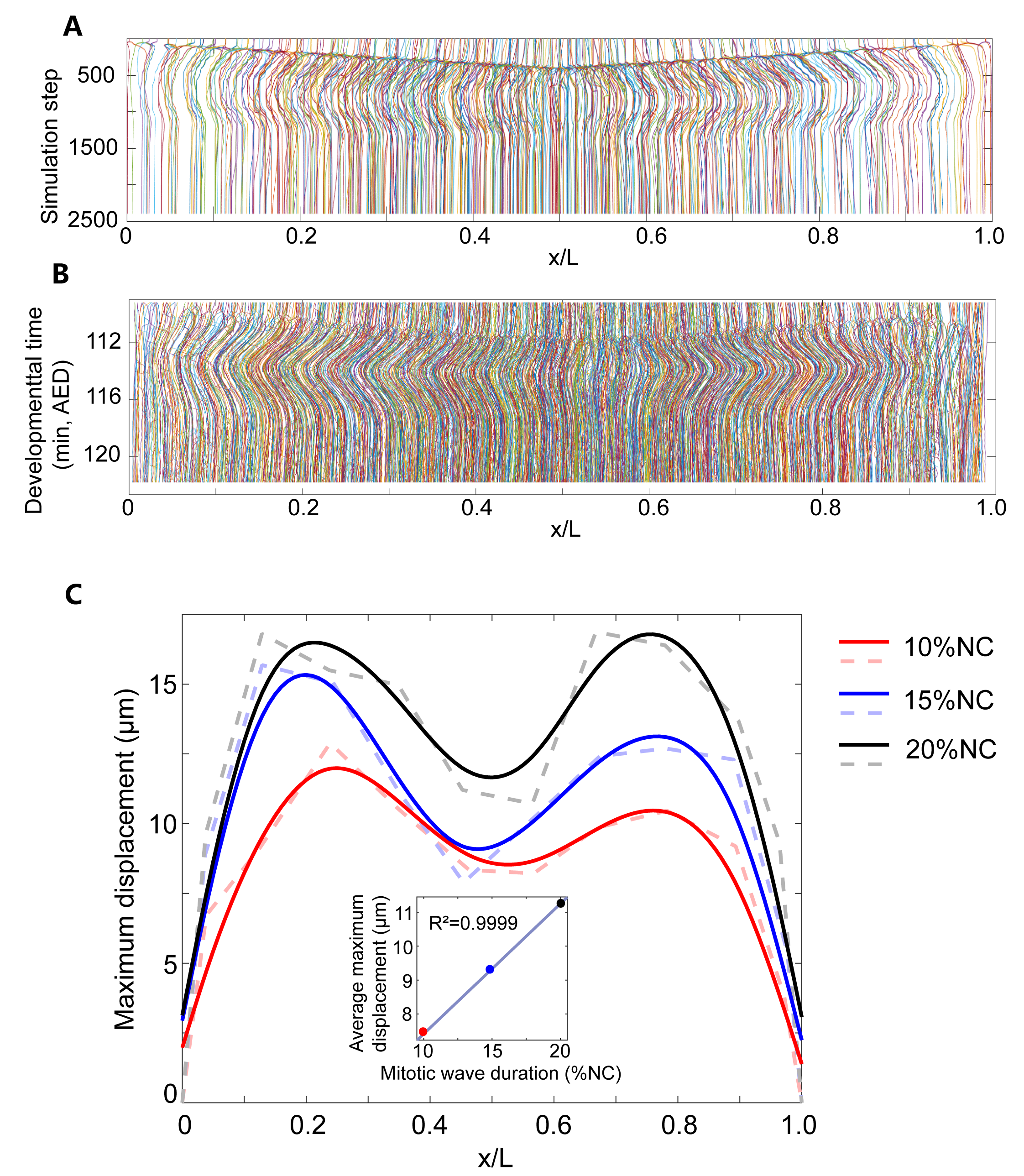

Supplement: S19 Fig — (A) Nuclear trajectories of 3D simulations in Fig 4. (B) Nuclear trajectories of experimental data in Fig 2 during interphase 13. (C) The maximum nuclear displacement along the AP axis in 3D simulations with different mitotic wave durations. The labels “*%NC” indicate the mitotic wave duration, e.g., 10%NC indicate the mitotic wave duration is 10% nuclear cycle time. The mitotic wave speed ratio of the three mitotic wave durations is 6(10%NC): 4(15%NC): 3(20%NC). Dashed lines are the original data and full lines are the fitted curves. The maximum displacement simulation data is consistent with the maximum displacement experimental data in ref [3]. The force field used in these simulations is shown in S16A Fig. (TIF) [file pcbi.1009605.s031.tif]

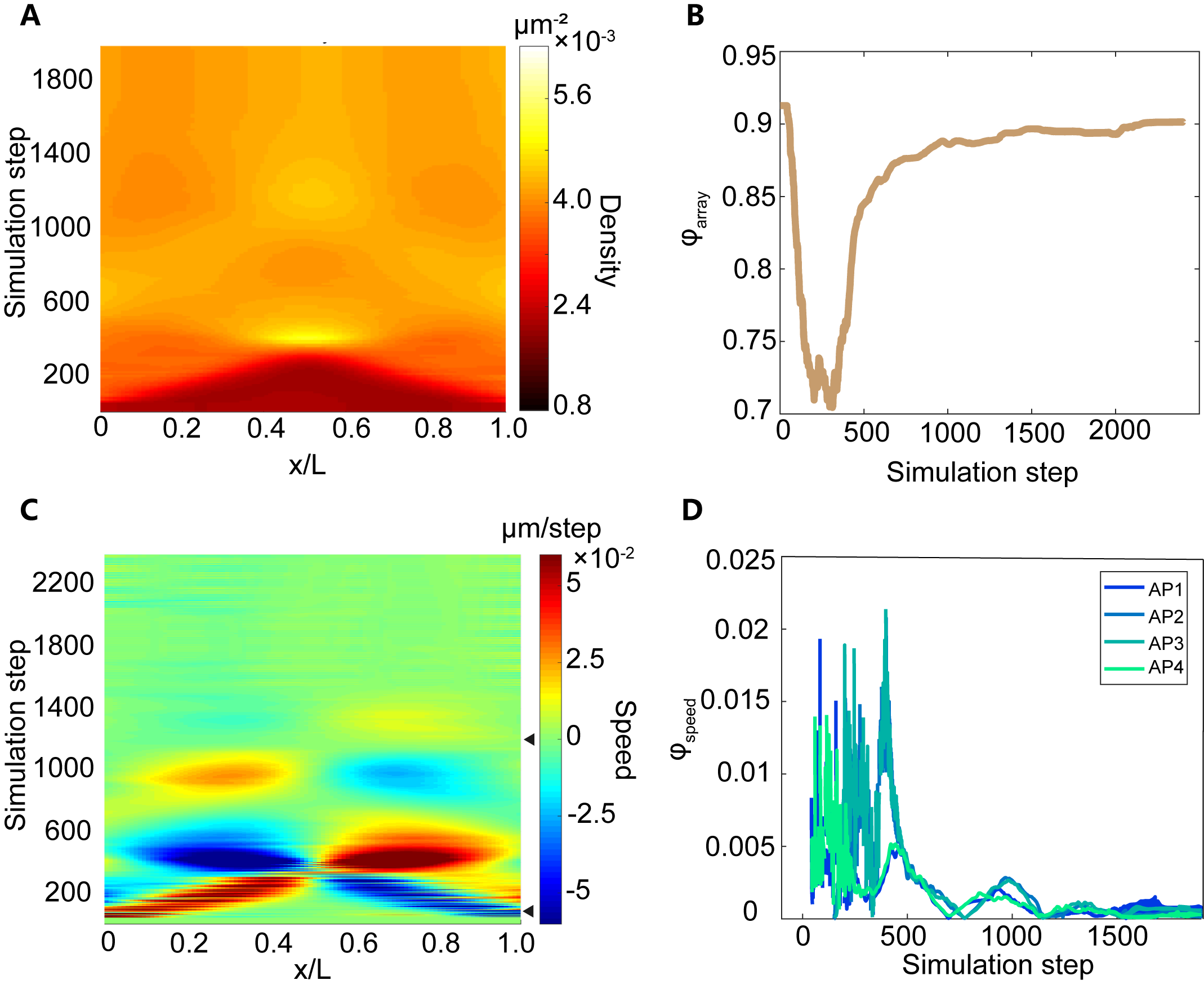

Supplement: S20 Fig — The characteristic features of the collective motion pattern and packing pattern of the nuclear array plots as in Fig 4A–4D. The force field used in this simulation is shown in S16B Fig. Black triangles in (C) mark two extra motion processes along the AP axis during simulation comparing to the experimental data. (TIF) [file pcbi.1009605.s032.tif]

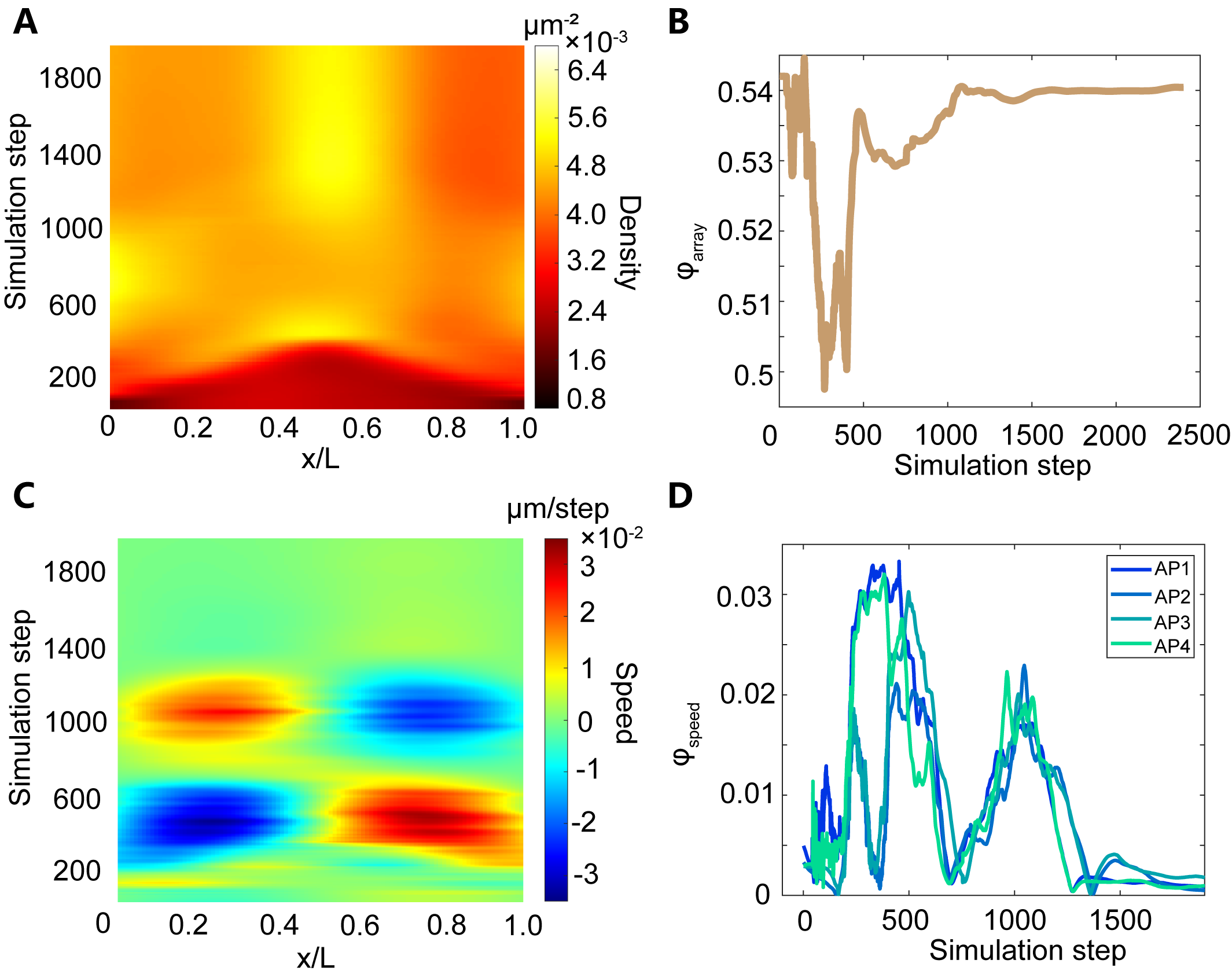

Supplement: S21 Fig — The characteristic features of the collective motion pattern and packing pattern of the nuclear array plots as in Fig 4A–4D. The internuclear force (F) has quadratic function relationship with the internuclear distance (r) instead of the linear relationship in Fig 4 (see S5 Text). (TIF) [file pcbi.1009605.s033.tif]

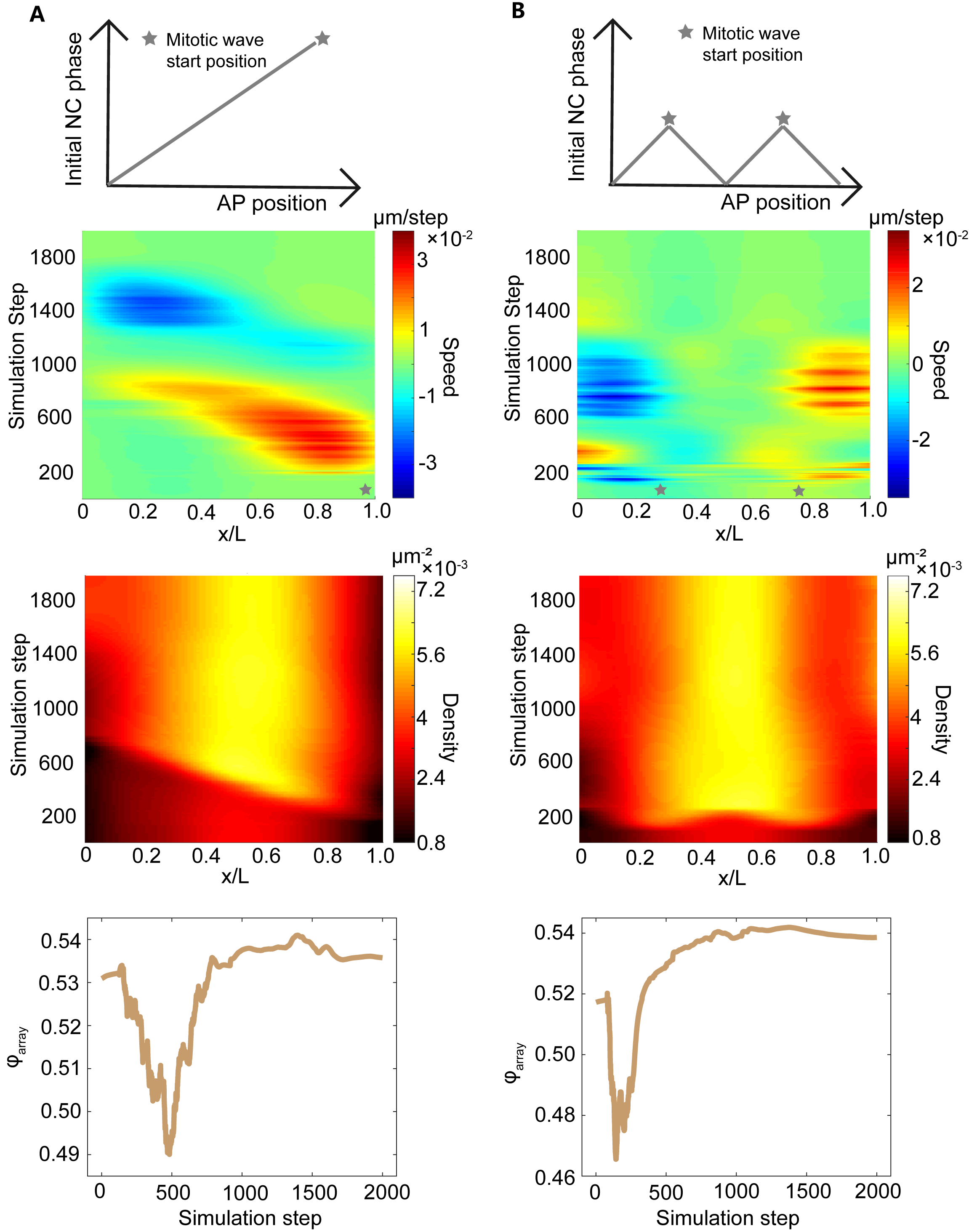

Supplement: S22 Fig — The force field used in this simulation is shown in S16A Fig. (A) The mitotic wave starts from one pole of the embryo. (B) The mitotic wave starts from a quarter point and three quarters point of the embryo. Gray stars indicate the mitotic wave start time point. The figures below are the corresponding heat maps of the AP speed, the AP nuclear density and the dynamics of hexatic bond-orientational order parameter. (TIF) [file pcbi.1009605.s034.tif]

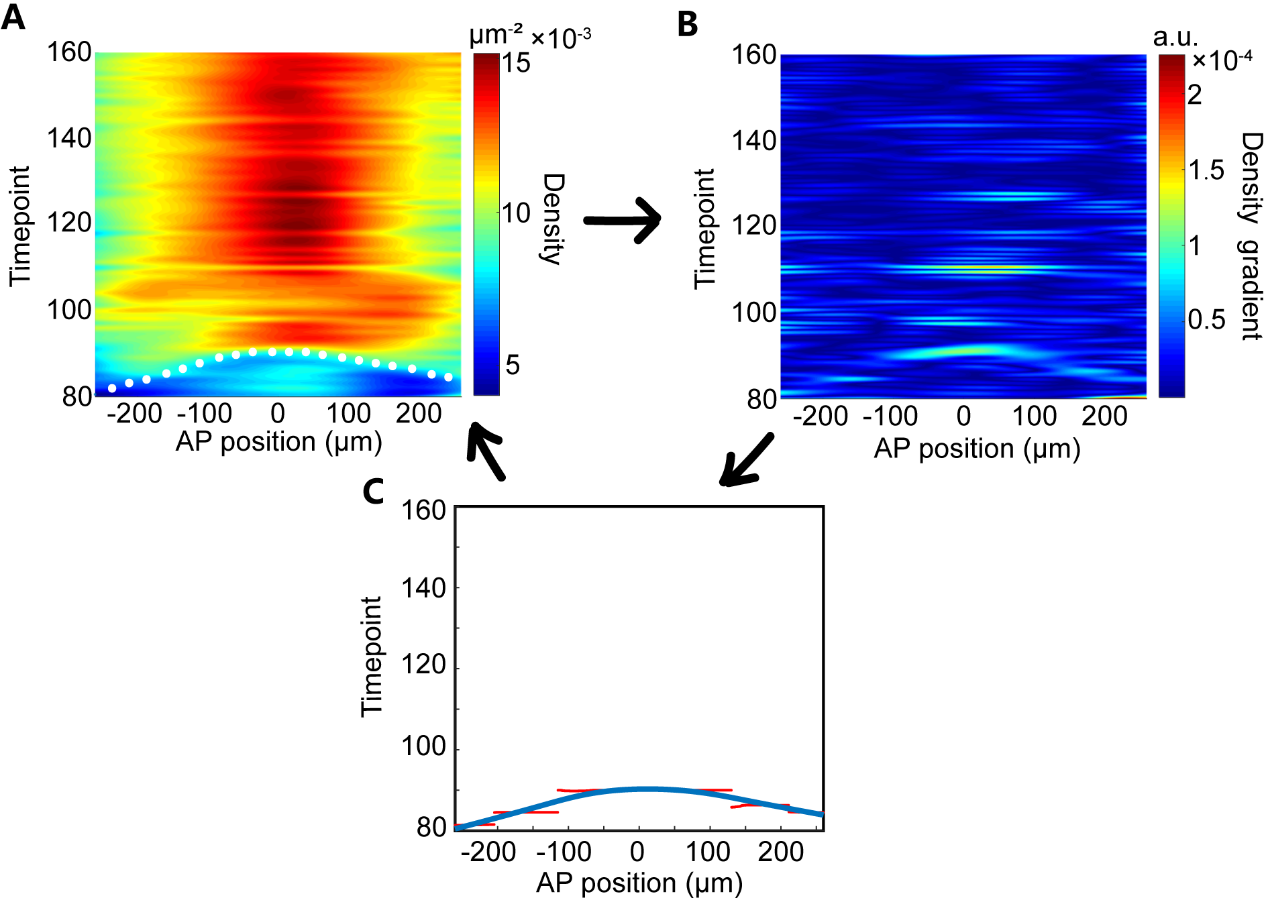

Supplement: S23 Fig — The density gradients (B) were calculated from one dimensional density data (A). The maximum density gradient values around the mitotic phase were identified (red lines in C) and fitted with a smooth spline (blue line in C). The corresponding time points indicating the metaphase anaphase transition time points in each nuclear cycle are labeled in A as white dots. (TIF) [file pcbi.1009605.s035.tif]
